# Robust helix photo-transforming in soft matter

**DOI:** 10.1093/nsr/nwaf572

**Published:** 2025-12-13

**Authors:** Mengqi Li, Honglong Hu, Zhi-Gang Zheng, Xueqian Niu, Conglong Yuan, Peizhi Sun, Xuan Liu, Xinrui Liu, Qi Zhang, He Tian, Wei-Hong Zhu, Ben L Feringa

**Affiliations:** Key Laboratory for Advanced Materials and Joint International Research Laboratory of Precision Chemistry and Molecular Engineering, Shanghai Key Laboratory of Functional Materials Chemistry, Feringa Nobel Prize Scientist Joint Research Center, Institute of Fine Chemicals, Frontiers Science Center for Materiobiology and Dynamic Chemistry, School of Chemistry and Molecular Engineering, East China University of Science and Technology, Shanghai 200237, China; Center of Photosensitive Chemicals Engineering, East China University of Science and Technology, Shanghai 200237, China; Key Laboratory for Advanced Materials and Joint International Research Laboratory of Precision Chemistry and Molecular Engineering, Shanghai Key Laboratory of Functional Materials Chemistry, Feringa Nobel Prize Scientist Joint Research Center, Institute of Fine Chemicals, Frontiers Science Center for Materiobiology and Dynamic Chemistry, School of Chemistry and Molecular Engineering, East China University of Science and Technology, Shanghai 200237, China; Center of Photosensitive Chemicals Engineering, East China University of Science and Technology, Shanghai 200237, China; School of Physics, East China University of Science and Technology, Shanghai 200237, China; Center of Photosensitive Chemicals Engineering, East China University of Science and Technology, Shanghai 200237, China; School of Physics, East China University of Science and Technology, Shanghai 200237, China; Key Laboratory for Advanced Materials and Joint International Research Laboratory of Precision Chemistry and Molecular Engineering, Shanghai Key Laboratory of Functional Materials Chemistry, Feringa Nobel Prize Scientist Joint Research Center, Institute of Fine Chemicals, Frontiers Science Center for Materiobiology and Dynamic Chemistry, School of Chemistry and Molecular Engineering, East China University of Science and Technology, Shanghai 200237, China; Key Laboratory for Advanced Materials and Joint International Research Laboratory of Precision Chemistry and Molecular Engineering, Shanghai Key Laboratory of Functional Materials Chemistry, Feringa Nobel Prize Scientist Joint Research Center, Institute of Fine Chemicals, Frontiers Science Center for Materiobiology and Dynamic Chemistry, School of Chemistry and Molecular Engineering, East China University of Science and Technology, Shanghai 200237, China; School of Physics, East China University of Science and Technology, Shanghai 200237, China; School of Physics, East China University of Science and Technology, Shanghai 200237, China; School of Physics, East China University of Science and Technology, Shanghai 200237, China; Key Laboratory for Advanced Materials and Joint International Research Laboratory of Precision Chemistry and Molecular Engineering, Shanghai Key Laboratory of Functional Materials Chemistry, Feringa Nobel Prize Scientist Joint Research Center, Institute of Fine Chemicals, Frontiers Science Center for Materiobiology and Dynamic Chemistry, School of Chemistry and Molecular Engineering, East China University of Science and Technology, Shanghai 200237, China; Key Laboratory for Advanced Materials and Joint International Research Laboratory of Precision Chemistry and Molecular Engineering, Shanghai Key Laboratory of Functional Materials Chemistry, Feringa Nobel Prize Scientist Joint Research Center, Institute of Fine Chemicals, Frontiers Science Center for Materiobiology and Dynamic Chemistry, School of Chemistry and Molecular Engineering, East China University of Science and Technology, Shanghai 200237, China; Key Laboratory for Advanced Materials and Joint International Research Laboratory of Precision Chemistry and Molecular Engineering, Shanghai Key Laboratory of Functional Materials Chemistry, Feringa Nobel Prize Scientist Joint Research Center, Institute of Fine Chemicals, Frontiers Science Center for Materiobiology and Dynamic Chemistry, School of Chemistry and Molecular Engineering, East China University of Science and Technology, Shanghai 200237, China; Key Laboratory for Advanced Materials and Joint International Research Laboratory of Precision Chemistry and Molecular Engineering, Shanghai Key Laboratory of Functional Materials Chemistry, Feringa Nobel Prize Scientist Joint Research Center, Institute of Fine Chemicals, Frontiers Science Center for Materiobiology and Dynamic Chemistry, School of Chemistry and Molecular Engineering, East China University of Science and Technology, Shanghai 200237, China; Center of Photosensitive Chemicals Engineering, East China University of Science and Technology, Shanghai 200237, China; Key Laboratory for Advanced Materials and Joint International Research Laboratory of Precision Chemistry and Molecular Engineering, Shanghai Key Laboratory of Functional Materials Chemistry, Feringa Nobel Prize Scientist Joint Research Center, Institute of Fine Chemicals, Frontiers Science Center for Materiobiology and Dynamic Chemistry, School of Chemistry and Molecular Engineering, East China University of Science and Technology, Shanghai 200237, China; Stratingh Institute for Chemistry and Zernike Institute for Advanced Materials, Faculty of Science and Engineering, University of Groningen, Groningen 9747 AG, The Netherlands

**Keywords:** photochromism, liquid crystals, helical twisting power, chiral photoswitch, circularly polarized luminescence

## Abstract

Helical organization in soft materials is omnipresent in systems ranging from DNA and peptides to liquid crystal displays. Dynamic transformation and reconfiguration of helicity triggered non-invasively by light are highly desirable, yet challenging to control in soft-condensed matter. Herein, we report the photo-transformation of helicity in soft matter with robust manipulation of the helical pitch and inversion of chirality. The key molecular design is based on the introduction of a multi-branched dendron-like chiral photoswitch, along with balancing long-range order and short-range disorder states, featuring ultra-large helical twisting power (HTP) and initiating an extremely broad dynamic spectral range (400–3000 nm). The resonance coupling between helixes and inherent luminescence of the chiral photoswitch enables stimulated circularly polarized luminescence (CPL), with a dissymmetric factor of 1.97 approaching the theoretical limit. The precise dynamic control allows for photo-tailorable infrared beams and high dimensional coding, offering a robust approach to dynamic soft matter, chiro-optics and information processing.

## Teaser

A chiral photoswitch with ultra-large helical twisting power promotes robust helix transforming in soft matter enabling information photonics.

## INTRODUCTION

Chirality is ubiquitous in nature and essential in the control of organization along length scales ranging from molecules to supramolecular, mesoscopic and macromolecular materials prominent in helical systems featuring molecular short-range interaction and mesoscale long-range order [1–3]. The dynamic photo-transformation of helicity covers several interdisciplinary challenges key to soft matter condensed physics [4–7], chemistry [8–10] and photonics [11–13] with prospects for both fundamental sciences and engineering. In particular liquid crystals (LCs) feature diverse programmable soft architectures with strong response upon weak external stimuli [14], which is an ideal platform to study the dynamic helix transforming at the molecular and mesoscopic scale using light [15–22]. Limited efforts have been made by introducing intrinsic chiral photoswitches to manipulate LC materials [23–26]. However, the design of systems with excellent thermal bistability and efficient helix transforming capability triggered by light, remains a fundamental challenge.

**Figure 1.**
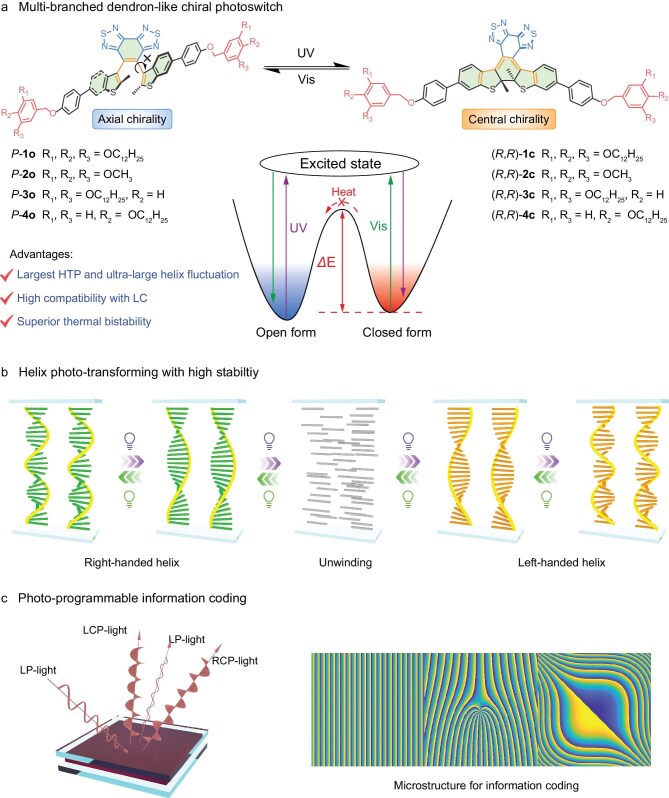
Multi-branched dendron-like chiral photoswitch with enantiospecific transformation enabling photo-transforming of soft helixes. (a) Enantiospecific isomerization of the chiral photoswitch between open form (**1o-4o**) and closed form (**1c-4c**) from axial chirality to central chirality. The inset figure shows a high energetic barrier between open and closed form, suggesting the high thermal stability of the chiral photoswitch, which is free of thermal relaxation. (b) The large helix transforming capability of *P*-**1o** realizing the photochemical manipulation over an extremely broad range of the helical pitch and light-switchable chiral invertible helical superstructure. (c) Photo-programmable information coding based on reflected structured infrared light. Note: ‘LP’, ‘LCP’ and ‘RCP’ denotes linear polarized, left-handed circularly polarized and right-handed circularly polarized, respectively.

Herein, we present a robust system for photo-transformation of helicity in soft matter by introducing a unique multi-branched dendron-based inherent chiral photoswitch which features enantiospecific transformation, superior thermal bistability and high compatibility in LCs (Fig. 1). This chiral photoswitch shows unprecedented helix transforming capabilities including the current highest helical twisting power (HTP) value with dynamic tunability and ultra-large fluctuation of the chiral dipole interaction, allowing efficient manipulation of the helical pitch as well as photochemical induced thermodynamic inversion of the helical twist (Fig. 1b). In addition, it allows the promotion of an extremely broad dynamic range in the reflection spectra, covering both the entire visible band and near infrared (NIR) regions (400–3000 nm). To comprehensively evaluate the helix transforming performance of these chiral photoswitches, we have defined for the first time a factor for photo-manipulating robustness (*R*_P_), manifesting an ultra-high value with one to three orders of magnitude higher than those of state-of-the-art chiral photoswitches. Taking advantage of the interdependence of the molecular organization in the soft helix material and inherent luminescence of the chiral photoswitch, we achieve photo-stimulating circularly polarized luminescence (CPL) with ultra-high dissymmetric factor (*g*_lum_) up to 1.97, approaching the theoretical value of 2.0. Furthermore, the robust helix photo-transforming facilitates dynamic photo-tailoring of the infrared optical field, establishing an all-photonic ternary spatiotemporal information coding system (Fig. 1c), and permitting understanding of the underlying logics of dynamic evolution, manipulation, reconfiguration and transformation in a LC soft materials system.

## RESULTS AND DISCUSSION

### Chiral switches allow robust helix photo-transforming

The prerequisite for achieving robust dynamic transformation of helixes is to design an inherent chiral photoswitch with large HTP value, fluctuation of the dipole interaction, superior thermal stability and fast response time. Here, we first defined the photoresponsive robustness (*R*_p_) in order to comprehensively evaluate the transforming capability upon irradiation of a chiral photoswitch in soft matter (Supplementary text 1.4):


(1) \begin{eqnarray*}
{R}_{\mathrm{p}}\ {\mathrm{ = \ }}{e}^t\ {\mathrm{\Delta HTP}}\ {c}^{ - 1}v,
\end{eqnarray*}

where *t* is thermal stability time of the chiral photoswitch in the soft helix, *v* is the average shift rate of the reflection band of the LC material, ΔHTP is the variation of HTP value before and after irradiation, and *c* is the molar ratio of photoswitch to mesogen.

To promote the *R*_P_ value, we designed a series of multi-branched dendron-like chiral photoswitches with a benzobis(thiadiazole) ethene bridge (**1–4**, with different alkyl chains number and length, Fig. 1a; for synthesis and characterization, see Schemes S1–S8 and Figs S1–S30; for photoresponsive performance, see Figs S31–S37), showing a unique enantiospecific transformation along with excellent thermal stability and fatigue resistance. We envision that the pending multi-branched polyalkyl groups likely enhance dopant–host compatibility with LC molecules and increase the stability of the mesophase, and that the large steric effect of multi-branched groups will result in ultra large HTP value and helix fluctuations.

**Figure 2.**
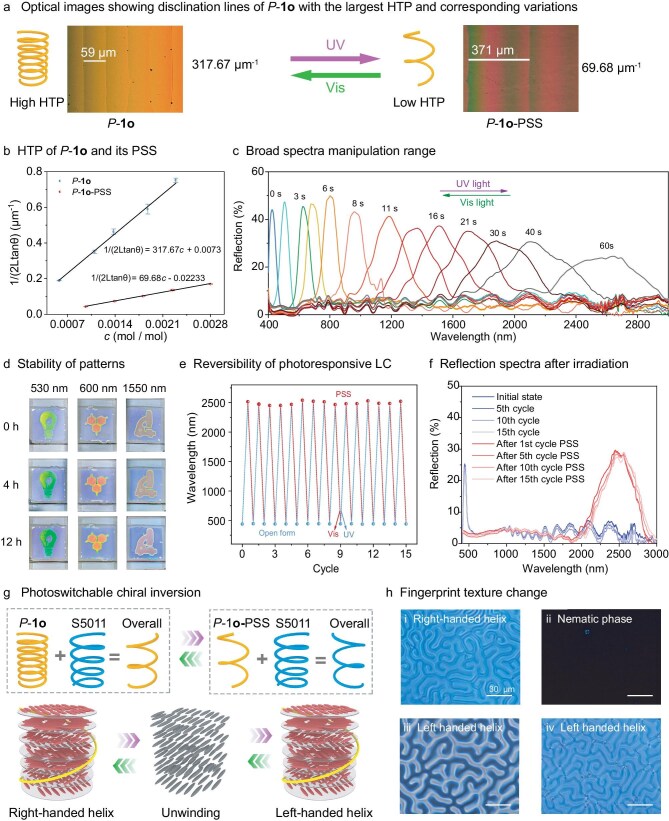
Photochemical helix transformation in soft helical LC material. (a) Optical images showing the change in disclination lines of the HTP of *P*-**1o** in LCs before and after 365-nm irradiation. (b) HTP of *P*-**1o** and corresponding photostationary state (PSS) in LCs. (c) Reflection wavelength of LC film containing 1.98 mol% *P*-**1o** in commercially available LCs in a 4-μm thick planar cell upon exposure to 365 nm light with different irradiation time. (d) Photowriting of a predefined pattern with 365-nm light passing through different photomasks. Three intermediate states reflecting green, red and dull red colors were preserved under dark ambient conditions for 4 h and 12 h after removing the light stimulus, presenting no distinct color migration (or blurring of the boundary). (e) Reversible change of the wavelength upon alternate irradiations with UV (*λ* = 365 nm) and visible light (*λ* >510 nm), respectively. (f) Reflection spectra of the photoresponsive helical LC in different cycles. (g) Illustration of the mechanism of the photoswitchable chirality upon an invertible helical superstructure by adding nonresponsive chiral dopants with opposite handedness. (h) Polarized optical microscopy (POM) images of the photoresponsive helical LCs in E7 in a 4-μm thick homeotropic cell upon exposure to 365 nm light.

Upon incorporation of long alkyl chains, the HTP value of *P*-**1o** (with six dodecyl chains) is as large as 317.67 μm^−1^, which is the largest HTP value among current chiral photoresponsive dopants (Fig. 2a and b, Fig. S38) [15,27–31]. In contrast, *P*-**2o** and *P*-**3o** with the corresponding short chain methoxy groups and four dodecyl chains only showed medium HTP value ∼214.21 and 263.71 μm^−1^, respectively (Figs S39–S42, Table S1). After UV irradiation, the HTP value of *P*-**1o** decreased dramatically to 69.68 μm^−1^, showing ultra-large fluctuation on the chiral dipole moment, i.e. the manipulation range of the HTP value is ∼247.99 μm^−1^ (Fig. 2a and b, Fig. S38). This large variation in HTP enables a huge range to dynamically control the helical superstructure of the LC material by irradiation. According to Bragg’s Law (*λ* = *nP*), the reflection band (photonic band gap, PBG) of the helical LC is determined by helical pitch (*P*) in the LC film. Notably, the photoresponsive LC film exhibited an extremely broad manipulation range of the reflection spectra from 400 to 3000 nm and fast response speed of 60 s upon UV light irradiation, along with a color change from dark blue, green and red to almost invisible color, covering the entire visible and NIR region (Fig. 2c, Fig. S43 and Video S1). A similar performance on reflection spectra was achieved in the system containing *P*-**3o** with higher concentration and more defects, compared to *P*-**1o** (Fig. S45). *P*-**2o** with poor solubility in LCs, only exhibited a narrow manipulation range from 400 to 650 nm (Fig. S44). Compared with azobenzene-based LC systems featuring inherent thermal relaxation [32,33], such a broad dynamic range ∼2600 nm with remarkable photo-reversibility and high multi-stability is, to the best of our knowledge, unprecedented in previous reported photo-responsive LC systems (Fig. 2e and f), benefiting from the superior high bistability and reversibility of the inherent chiral photoswitch (Fig. S37). Thus, the photoresponsive LCs exhibited excellent multi-stability, as illustrated by the unchanged reflection band and a series of robust well-defined patterns at different intermediate states after removing the light stimulus for a long period of time (Fig. 2d and Fig. S46). A photothermal effect by photoirradiation is also excluded (Fig. S47).

Chirality is an intrinsic topological attribute usually fixed in materials processing. The photo-induced chirality in a non-chiral material [34–36] inspired us to design the high stability chiral photoswitch, enabling chirality inversion in a LC with light modulation from right-handed, via nonchiral, to left-handed helicity, and featuring thermodynamic stability at any intermediate state. Such ultra-large HTP and helix fluctuation of the given photoswitch facilitates the design of photo-invertible LC materials, by precisely controlling the constituent ratio between photoresponsive and nonresponsive dopants, to further extend current helical pitch modulation to control helical chirality (Fig. 2g). The chirality inversion and helical pitch can be clearly characterized by the space between the stripes of the fingerprint texture in a homeotropic cell, which shows elongation of the helical pitch of the LCs during UV irradiation (Fig. 2h). Continuous irradiation will result in a gradual disappearance of the fingerprint texture until reaching a transient nematic phase, due to the unwinding of the soft helixes. Further illumination leads to a re-emerging fingerprint texture, indicating the chirality inversion from right- to left-handed helix accompanied by a decrease of helical pitch (Fig. 2h, Fig. S48, Videos S2 and S3). These results highlight the essential role of incorporating branched groups with increased number and extended chain lengths into these photoswitches, enhancing the miscibility of hybrid LCs and larger intermolecular interactions with LC molecules resulting in unmatched ability to transfer the intrinsic axial chirality of the molecule to the mesoscale of LC helixes.

Therefore, we accomplished a robust helix photo-transformation by taking advantage of such a multi-branched dendron-like chiral photoswitch (*P*-**1o**), which possesses an ultra-high *R*_P_ value, showing orders of magnitude higher than other reported chiral photoswitches thus far (Fig. S49, Fig. S50 and Table S2) [32]. Such robust helix photo-transformation originates from the unique intrinsic chirality of sterically hindered diarylethene building blocks with enantiospecific photoreaction, which yield the largest HTP value, ultra-large variations and extremely wide manipulation range. Furthermore, the introduction of multi-branched groups enhances the strong and anisotropic shape of intrinsic chiral photoswitches, which promote precise alignment and high compatibility with the LC host, facilitating robust helix-transformation. This chiral photoswitch in LCs further facilitates the control of photo-modulation of circularly polarized luminescence, multi-dimensional light tailoring and information processing.

### Photo-stimulating CPL with an approximately theoretical dissymmetric factor of 1.97

**Figure 3.**
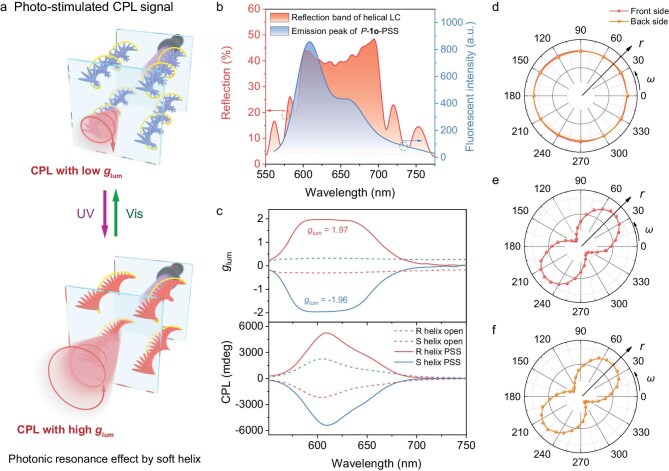
Helix photo-transforming enabling photo-modulated circularly polarized luminescence. (a) Graphical representation of the photomodulation on the reflection band to achieve high *g*_lum_ based on internal emission of the AIE effect of *P*-**1o**. (b) Reflection spectra of the helical superstructure and emission spectrum showing complete overlap with the AIE peak of *P*-**1o** at PSS in the LCs. (c) The *g*_lum_ values and CPL spectra upon excitation at 326 nm when the reflection spectra totally (solid lines) and marginally (dash lines) overlap with luminescence, respectively. (d–f) Emission intensity in HTD-200 of circularly polarized luminescence at 610 nm as a function of the polarization angle in the polar coordinate system: (d) light intensity of CPL of photoresponsive LC (0.39 mol% *P*-**1o** and 1.6 mol% R5011) at front side and back side of the LC cell after passing through the polarizer; light intensity of CPL of photoresponsive LC (0.39 mol% *P*-**1o** and 1.6 mol% R5011) at (e) front side and (f) back side of the LC cell after passing through a quarter waveplate and the polarizer.

Photo-stimulation to generate and regulate CPL is of great significance particularly for 3D display and information encoding systems, requiring both tunability and a high dissymmetric factor [37–40]. The photonic resonance effect in helical organized materials enables coupling with the inherent luminescence of the chiral photoswitch (Figs S51 and S52) [41]; we anticipated that this new responsive chiral dopant with remote photo-stimulation could lead to the generation and regulation of CPL with an almost theoretical dissymmetric factor (Fig. 3a) [42].

First it is established that there is complete overlap between the PBG and the aggregation-induced emission (AIE) spectra in helical LC systems (Fig. 3b and Fig. S53). The luminescent LCs present a strong positive CPL signal with ultra-high *g*_lum_ value up to 1.97, which is the current highest *g*_lum_ value to date (Fig. 3c and Table S1) [43]. CPL polarization is further evaluated by analyzing the azimuthal angle-dependent transmission, thereby confirming circular polarization (Fig. 3d, Fig. S54 and Fig. S55c). On the contrary, the CPL signal in the case of incomplete spectral overlap with small birefringence LC only results in a medium *g*_lum_ value of 1.37 (Figs S56 and S57). When the reflection band of the photoresponsive LC system is shifted to show marginal overlap with the AIE peak by visible light stimulation (Fig. S53), it only generates a negative CPL signal with low *g*_lum_ factor about −0.3 (Fig. 3c and Fig. S56, red dash line). Therefore, the CPL shows exceptional high *g*_lum_ induced by photo-stimulation, which can be modulated by irradiation. In addition, the extremely high *g*_lum_ factor of the CPL systems remains unaffected by differences in sample batches or illumination conditions, and displays robust reversibility by UV (365-nm light) and visible light (*λ* >510 nm) irradiation, with high *g*_lum_ factor in the range of 1.94–1.98 (Fig. S58). Specifically, the handedness of the CPL at different sides of the LC cell manifested the same sign and the circular dichroism spectra showed strong signals at the reflection band (Fig. 3e and f, Figs S54–S60) [44], supporting a mechanism involving precise coupling between the inherent luminescence and photonic resonance in the LC helical structure [42,45]. Such photo-stimulating CPL with the highest *g*_lum_ value so far provides major prospects for future optical field modulation, optical information processing and related applications in other fields.

### Multi-dimensional photo-programming infrared beam

**Figure 4.**
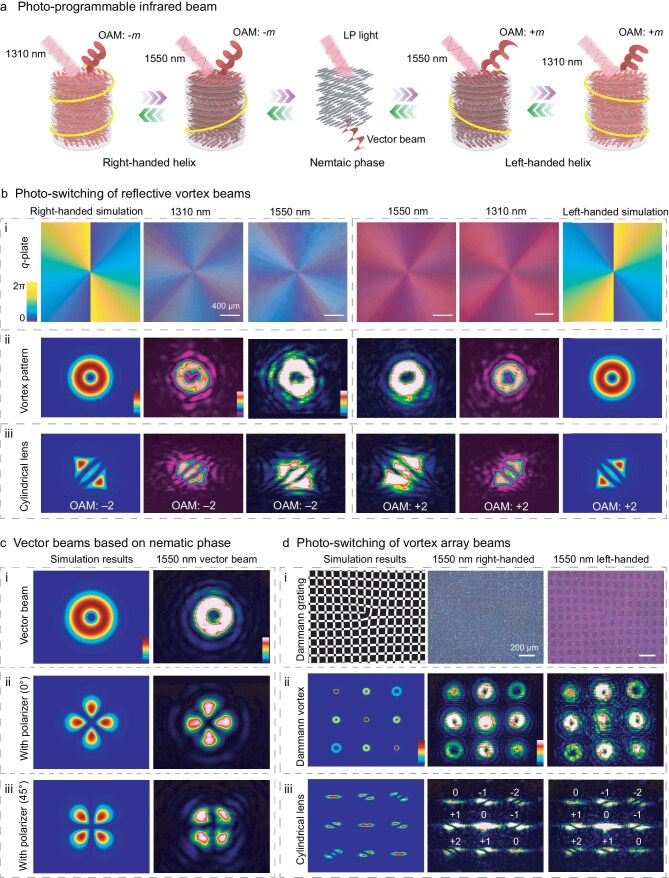
Helix photo-transforming enabling a multi-dimensional photo-programmable infrared beam. (a) Schematic illustration of the multi-dimensional photo-programmable infrared beam, showing symmetry switching of the OAM between ±*m* and the switching between the scalar beam and the vector beam. (b) i: Theoretical phase profiles, micrographs of the Q-plate (*m* = ±2) with right- and left-handed helical LC at 1310 and 1550 nm; ii: simulated and measured reflected diffraction vortex beam; iii: corresponding phase topological charge detection by cylindrical lens at 1310 and 1550 nm. The scale bar is 400 μm. (c) i: Simulated and generated vector beam at 1550 nm; ii–iii: corresponding transmission patterns using a polarizer with different angles (0° or 45°). The vector beam can be obtained when LP light is transmitted through a nematic phase Q-plate during the chiral inversion process. (d) i: Binary phase structure of Dammann vortex grating (DVG) with alternate 0 and π phase of *m_x_* = *m_y_* = 1, where white indicates 0 and black indicates π, and corresponding micrographs with right- and left-handed helical LC at 1550 nm; ii: simulated and measured reflected diffraction patterns; iii: corresponding phase topological charge detection by cylindrical lens at 1550 nm. The scale bar is 200 μm.

Photo-transforming helixes in LC can realize the generation and dynamic programming of an optical field on demand. Inspired by our photo-invertible soft helix, we adjusted the reflection spectral range to the infrared region (Figs S61 and S62), and further studied the possibility of multi-dimensional photo-programming of the infrared beam (Fig. 4a), such as wavefront, orbital-angular momentum (OAM), polarization and wavelength [46–49], and as a result provide a framework for practical applications in high-capacity and parallel optical communication [50–52].

Initially, we designed a Q-plate with geometric phase distribution in a photo-invertible chiral LC (see Supplementary text, S1.5, Part 1), presenting the infrared vortex optical field (1550 and 1310 nm) at the reflection side (Fig. 4b–i, Fig. S63a and Supplementary text, S1.5, Part 2). Along with light irradiation, when the shifted reflection band matches the corresponding laser wavelength, a series of vortex diffractions with donut-like patterns are received at the reflection side, corresponding to the simulation results (Fig. 4b–i and 4b–ii, Videos S4 and S5) [53]. The phase topological charge (*m*) of the vortex beam was detected by a cylindrical lens, indicative of *m* = –2 and +2, corresponding to the right- and left-handed helix, respectively. Such phenomenon clearly manifests symmetry switching of the topological charge between ±*m* induced by the photo-invertible helix in the LC (Fig. 4b–iii, Videos S4 and S5) [54].

In addition, we achieved dynamic switching from the scalar optical field to the vector one at the transmitted side, due to the chiral inversion process (Fig. 4c, Fig. S63b, Fig. S48, Videos S6 and S7), which showed a spatially varying polarization state as supported by theoretical calculations (Fig. 4c–ii, Fig. S64–ii, Supplementary text, S1.5, Part 3 and Supplementary text, S1.6) [55]. Considering the dynamic tailoring of a vortex beam by helix photo-transforming, we can further realize more complex designs. In particular modulating the infrared optical field, through a specifically designed two-dimensional Damman vortex grating with geometric phase distribution (Fig. 4d–i), enables the photo-reversible manipulation of the infrared vortex array beam (Fig. 4d–ii, 4d–iii, Supplementary text, S1.5, Part 4 and Supplementary text, S1.6) [46]. As a result of this unprecedented large helix photo-transforming, we have realized multi-dimensional photo-programmable infrared beams, not limited to the aforementioned polarization, phase and spectrum. The symmetry switching of the topological charge between ±*m* and the switching between the scalar beam and the unique vector beam was also possible, thereby forming a basis for highly promising multi-dimensional information processing toward next generation all-photonic information systems [56]. Such multi-dimensional photo-programmable infrared beams can also be applied to optical manipulation and sorting, which is inherently suited for enantioselective sorting, facilitating separation of chiral nano- or microparticles with high specificity, and addressing the growing demand in pharmaceutical and biochemical applications [57].

### Establishing all-photonic ternary spatiotemporal coding

Considering the all-photonic regulation of the infrared optical field as shown here, it provides a unique material toward the realization of an all-optical information regulation system. Ternary coding possesses a closer logic to human thinking, thereby, compared to traditional binary coding, it features the advantage of higher information capacity and lower coding complexity [58,59].

**Figure 5.**
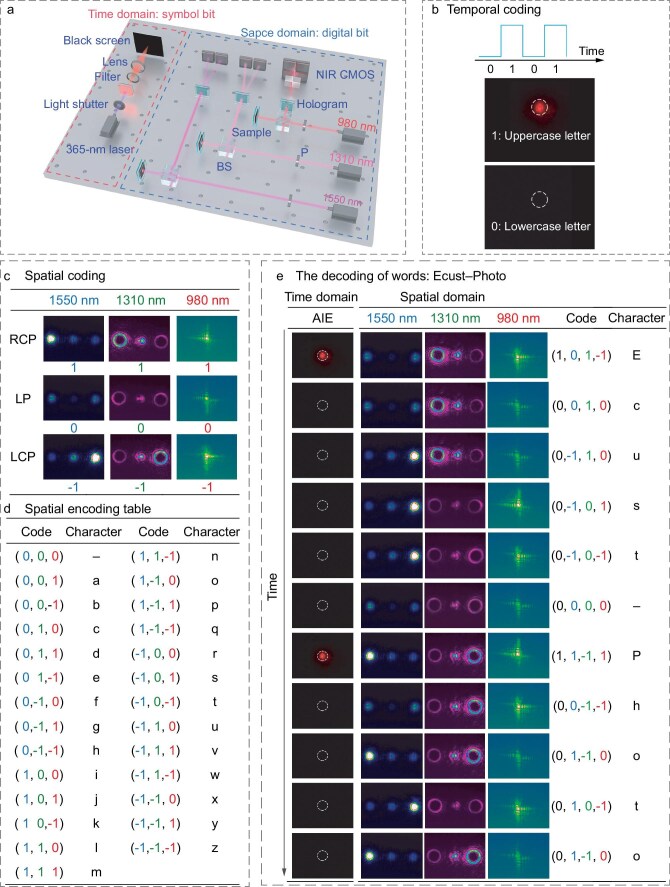
Helix photo-transforming establishing ternary spatiotemporal coding. (a) Optical setup of information coding. The 980-nm laser light passed through the polarizer (P) and beamsplitter (BS), and impinged on the sample. The reflected light was turned by the BS and transmitted through the Airy beam phase plate to generate corresponding diffraction patterns, which was measured by four NIR CMOS detectors. For other laser light a similar setup was applied. Three NIR lasers were used to illustrate the spatial encoding. A 365-nm laser was employed to excite the AIE luminescence of the luminescent chiral photoswitch to illustrate the temporal coding. (b) Illustration of temporal encoding by AIE fluorescence, the signal of which changed with time. (c) Illustration of spatial coding, showing the photoswitchable diffraction pattern among RCP light, LP light and LCP light of polarization grating detected by 1550-nm light, fork grating detected by 1310-nm light and Airy beams detected by 980-nm light to the corresponding code. (d) Spatial encoding table describing the one-to-one correspondence between the code and the characters. (e) Photoswitchable diffraction patterns of three different laser lights decoded into a series of words ‘Ecust–Photo’ by the spatiotemporal encoding system.

Taking advantage of helix photo-transforming using the chiral photoswitch with an ultra-high *R*_P_ value, we demonstrated a spatial encoding scheme [60] (Fig. 5a) by using different infrared wavelengths (980, 1310 and 1550 nm, typical communication band) and specific optical field (Fig. 1c, Figs S61, S62 and S65). Introducing the wavelength of the laser, the distribution of optical field and the relevant polarization state facilitated three-dimensional spatial coding, describing the one-to-one correspondence between the code and the 26 characters in the English alphabet (Fig. 5c and d, Videos S8-S10 and Supplementary text, S1.7). To further enhance the information density and coding efficiency, time evolution was introduced by leveraging the AIE fluorescence change of the chiral photoswitch to distinguish between the upper and lower case characters, thereby establishing an all-photonic spatiotemporal coding (Fig. 5a and b). Here, taking advantage of opportunities of the temporal and spatial encoding, we achieved the output of a series of characters ‘Ecust–Photo’ as shown in Fig. 5e and Video S11. We further integrated the sample using three holograms and beam-splitters to construct a demo device, presenting a ternary spatial information coding system manipulated by UV and visible light (Fig. S66 and Video S12). Such a photo-modulating ternary spatiotemporal encoding system affords a systematic and feasible strategy for large-capacity and multi-degree-of-freedom optical communications, distinctly enhancing the efficiency and safety of the communication process.

## CONCLUSIONS

Addressing the long-standing challenge to design inherent chiral photoswitches for LC materials that allow reversible and external control of helical organization covering the entire visible and infrared spectral range, we present a multi-branched dendron-based chiral switchable dopant featuring the current highest HTP and ultra-large helix fluctuations. The primary design feature comprises multi-branched groups attached to an intrinsic chiroptical diarylethene-type photoswitch which efficiently improves dopant–host compatibility and intermolecular interactions with LC molecules. As a result, it greatly enhances the helix transforming capability of the helical arrangement in soft LC materials and exhibits excellent modulation of photoinduced structural configuration, i.e. reversible elongation and contraction of the soft helical pitch and efficient thermodynamic switching of helical handedness. This robust helix photo-transforming enables the manipulation of reflection spectra over an exceptionally broad range, from 400 to 3000 nm, spanning the entire visible, NIR and even mid-infrared region and completely free of thermal relaxation. The unique coupling of helix photo-transforming with inherent luminescence facilitates the generation of photo-stimulating CPL emissions with a dissymmetric factor of 1.97, approaching the theoretical limit. The robust photo-modulated chiral inversion, with reversible multi-thermodynamic stable states, allows for photo-programmable infrared beams, laying the groundwork for an all-photonic ternary spatiotemporal information coding system. Overall, the fundamental study and discoveries presented here establishes a practical platform for dynamic soft matter and will accelerate the development of optical communications, biomimetic soft machines and future smart materials.

## METHODS

The detailed experimental methods can be found in the online Supplementary file.

## Supplementary Material

nwaf572_Supplemental_Files
