# Supplementary material for: Robust helix photo-transforming in soft matter
Source: Natl Sci Rev. 2025 Dec 13;13(3):nwaf572. doi: 10.1093/nsr/nwaf572 (PMC12866665; doi:10.1093/nsr/nwaf572)
Supplement: nwaf572_Supplemental_Files [file nwaf572_supplemental_files.zip › Supplementary data-r.pdf]

Supplementary Information

Robust helix photo-transforming in soft matter

Mengqi Li[1,2], Honglong Hu[1,2,3], Zhi-Gang Zheng[2,3,*], Xueqian Niu[1], Conglong Yuan[1,3], Peizhi Sun[3], Xuan Liu[3], Xinrui Liu[1], Qi Zhang[1], He Tian[1,*], Wei-Hong Zhu[1,2,*] and Ben L. Feringa[1,4,*]

[1]Key Laboratory for Advanced Materials and Joint International Research Laboratory of Precision Chemistry and Molecular Engineering, Shanghai Key Laboratory of Functional Materials Chemistry, Feringa Nobel Prize Scientist Joint Research Center, Institute of Fine Chemicals, Frontiers Science Center for Materiobiology and Dynamic Chemistry, School of Chemistry and Molecular Engineering, East China University of Science and Technology, Shanghai 200237, China;

[2]Center of Photosensitive Chemicals Engineering, East China University of Science and Technology, Shanghai 200237, China;

[3]School of Physics, East China University of Science and Technology, Shanghai 200237, China.

[4]Stratingh Institute for Chemistry and Zernike Institute for Advanced Materials, Faculty of Science and Engineering, University of Groningen, Groningen 9747 AG, The Netherlands.

***Correspondence authors**. E-mail: b.l.feringa@rug.nl, whzhu@ecust.edu.cn; tianhe@ecust.edu.cn; zgzheng@ecust.edu.cn;

This PDF file includes:

Supplementary Text
Figs. S1 to S66
Tables S1 to S3
References (1-16)

Other Supplementary Materials for this manuscript include the following:

Videos S1 to S12

1. Supplementary text

1.1 General

All commercially available starting reagents and solvents were used directly without further treatment unless otherwise specified. (4-Methoxyphenyl)boronic acid was purchased from Adamas Reagent Co., Ltd. BBTE-Br, compound **5-8** were prepared according to the established method[1-4]. All NMR spectra were recorded on Bruker AM-400 spectrometers with tetramethylsilane as an internal reference, $CDCl_3$ as solvents. High resolution mass spectra (HRMS) were recorded on a Waters LCT Premier XE spectrometer with methanol as solvent. Chiral HPLC analyses of **1o** were performed by using an Agilent 1200 instrument equipped with CHIRALCEL® Chiralpak ID (4.6 mm × 250 mm, eluent: methylene chloride/n-hexane = 45/55, v/v), and detecting at 290 nm. Chiral HPLC analyses of **2o** were performed by using an Agilent 1200 instrument equipped with CHIRALCEL® Chiralpak IK (4.6 mm × 250 mm, eluent: methylene chloride/n-hexane = 70/30, v/v), and detecting at 297 nm. Chiral HPLC analyses of **3o** were performed by using an Agilent 1200 instrument equipped with CHIRALCEL® Chiralpak IK (4.6 mm × 250 mm, eluent: methylene chloride/n-hexane = 60/40, v/v), and detecting at 254 nm. Absorption spectra were recorded on Agilent Cary 60 (1 cm quartz cell). Fluorescence spectra were recorded using HORIBA Fluoromax 4. The photochromic reaction was induced in situ by continuous irradiation using an Hg/Xe lamp (Hamamatsu, LC8 Lightningcure, 200 W) equipped with a narrow band interference filter (Shenyang HB optical Technology) for λ_{irr} = 313 ± 10 nm, a hand-held UV lamp (λ = 365 ± 20 nm, 1.0 mW cm^{-2}) or irradiation with a white LED equipped with a broad band interference filters λ_{irr} > 510 nm (2.0 mW cm^{-2}). Fluorescence quantum yields in LCs were measured by using an integrating sphere on a HAMAMATSU Quantaurus-QY C11347-11. CD spectra were recorded using JASCO J-819 spectropolarimeter (1 cm quartz cell) at 25 ºC. CPL spectra were acquired using the JASCO CPL-300 spectrofluoropolarimeter.

1.2 Liquid crystal systems

The nematic LC (TEB300, E7 and HTD-200) was supported by PhiChem, Shanghai, and chiral dopant R5011 and S5011 was supplied by HCCH, China. The LC samples were observed by a polarized optical microscopy (POM, Nikon LVPOL 100) with crossed polarizers under reflection mode, the optical textures were recorded by a charge-coupled device (CCD) camera, and a fiber coupled spectrometer and NIR near-infrared spectrometer (Avaspec-ULS2048, 200-1100 nm Avantes, Netherlands; NIR17s, 900-1700 nm, ideaoptics, China) were used to detect the reflection spectra of visible and NIR region from the sample. Reflection spectra during 1700-3000 nm were characterized by UV-3600Plus, Shimadzu. For reginal photoalignment technique, an optically sulfonic azo-dye SD1 (0.5 wt% in *N*,*N*-dimethylformamide) is spin-coated onto two indium tin oxide-coated glass substrates, and a Digital Micromirror Device based microlithography system was used to carry out the photoalignment process. The laser sources with output wavelengths of 980-nm, 1310-nm and 1550-nm were used to construct information coding. The diffraction pattern was received by a NIR CMOS camera (SWF640C-ACME-C2-G) behind optical grating. A quarter waveplate at the range from 350-850 nm and a polarizer were used to determine polarization properties of circularly polarized luminescence. Three quarter waveplates at 980, 1310 and 1550 nm and a polarizer at the range of 900-1700 nm were used to determined polarization properties of reflected laser beams from samples. The beamsplitters of 700-1100 and 1100-1700 nm were used to split and reflect the infrared laser beams. The temperature of LC cell during photoirradiation was detected by a thermal imager (TIS60, FLUKE, USA).

1.3 Synthesis and chiral separation of chiral photoswitch

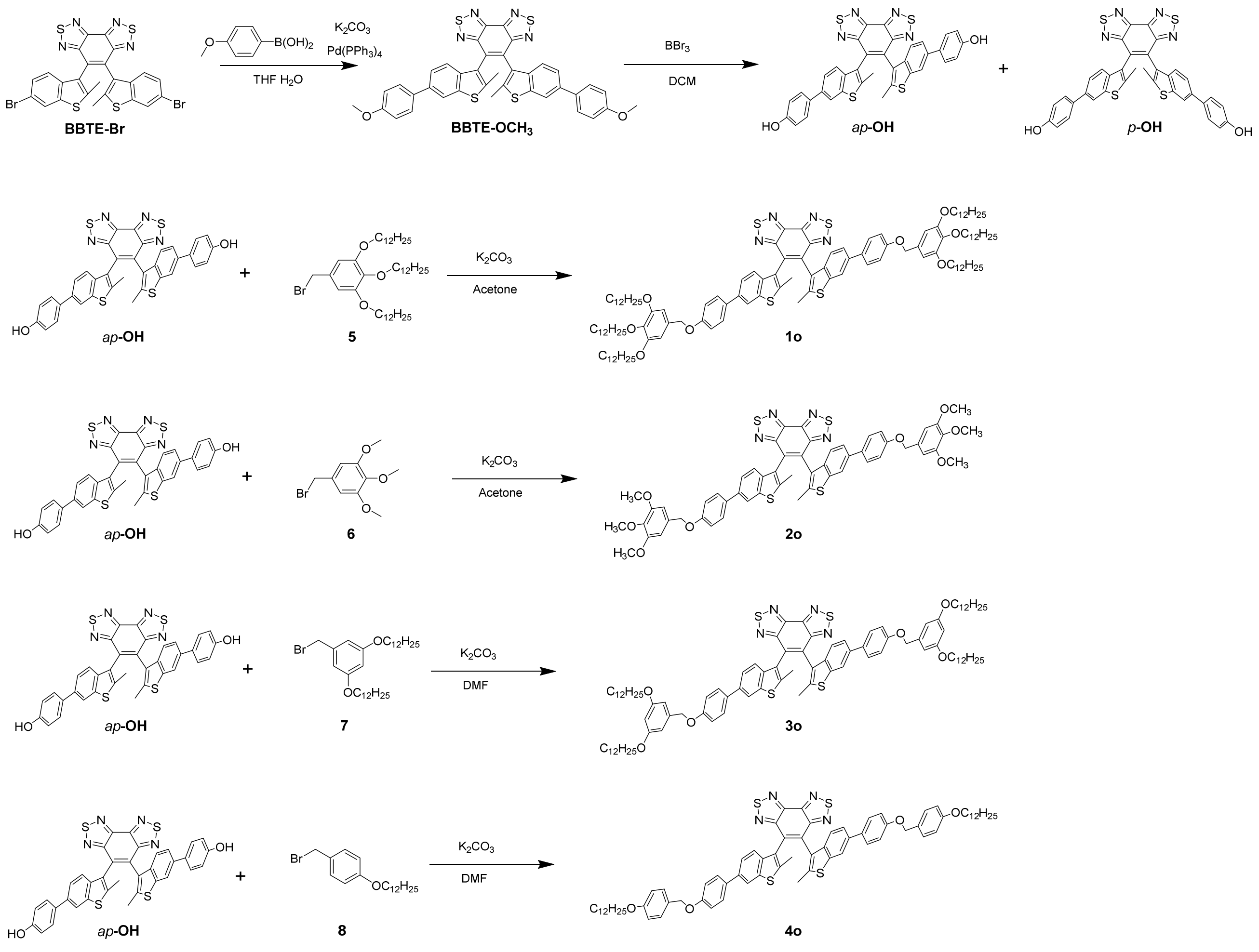

Scheme S1. Synthetic route of **1o-4o**

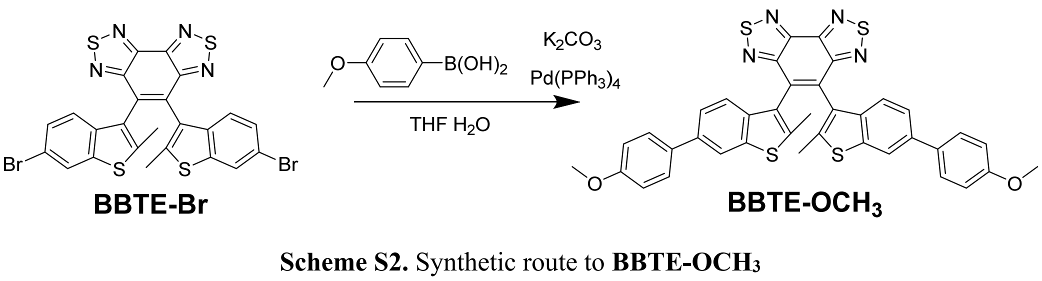

Scheme S2. Synthetic route to **BBTE-OCH**$_3$

Synthesis of BBTE-OCH$_3$: To a 250 mL single necked bottle, **BBTE-Br** (1.6 g, 2.49 mmol), (4-methoxyphenyl)boronic acid (2.84 g, 18.7 mmol), THF (145 mL), K_2CO_3 aqueous solution (2 M, 72 mL) and $Pd(PPh_3)_4$ (300 mg, 0.26 mmol) were added. The mixture was heated to 85°C under nitrogen atmosphere for 12 h in the dark, then the organic layer was separated and concentrated in vacuum. The residue and the water layer were extracted with methylene chloride (100 mL × 3). The organic layer was separated and dried by anhydrous Na_2SO_4. After concentrated in vacuum, the residue was purified by column chromatography on silica gel (methylene chloride: petrol ether = 2: 3) to get yellow solid in 73.4% (1.6 g).

BBTE-OCH$_3$: ^{1}H NMR (400 MHz, CDCl$_3$, ppm) δ: 1.90 (s, 6 H, -CH$_3$, *ap*-conformer), 2.26 (s, 6 H, -CH$_3$, *p*-conformer), 3.75 (s, 6 H, -OCH$_3$, *p*-conformer), 3.79 (s, 6 H, -OCH$_3$, *ap*-conformer), 6.85 (dd, J_1 = 8.0 Hz, J_2 = 2.0 Hz, 4 H, phenyl-H, *p*-conformer), 6.91 (dd, J_1 = 8.0 Hz, J_2 = 2.0 Hz, 4 H, phenyl-H, *ap*-conformer), 7.06-7.10 (m, 6 H, benzothiophene-H), 7.34 (dd, J_1 = 8.4 Hz, J_2 = 1.6 Hz, 2 H, benzothiophene-H, *ap*-conformer), 7.39 (dd, J_1 = 8.8 Hz, J_2 = 2.0 Hz, 4 H, phenyl-H, *p*-conformer), 7.50 (dd, J_1 = 8.0 Hz, J_2 = 2.0 Hz, 4 H, phenyl-H, *ap*-conformer), 7.73 (s, 2H, benzothiophene-H, *p*-conformer), 7.85 (d, J = 1.2 Hz, 2 H, benzothiophene-H, *ap*-conformer).

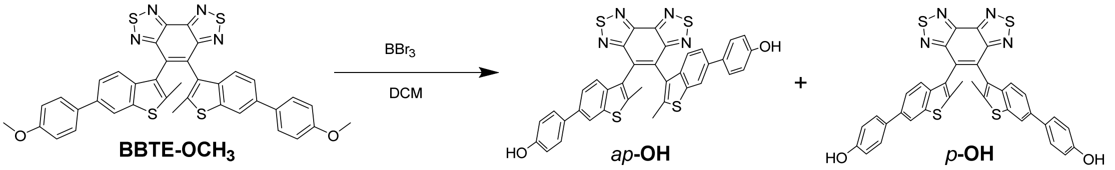

Scheme S3. Synthetic route to *ap*-**OH** and *p*-**OH**

Synthesis of *ap*-OH and *p*-OH: To a 250 mL double necked bottle, **BBTE-OCH$_3$** (1.6 g, 2.29 mmol) and anhydrous methylene chloride 160 mL were added. An anhydrous methylene chloride solution (13.7 mL) of BBr$_3$ (3.43 g, 13.7 mmol) was added dropwise into above solution in ice bath under nitrogen atmosphere. The mixture was stirred in ice bath for 1.5 h and then it was stirred in room temperature for 6 h. 10 mL water was added into above mixture to stop the reaction, and then extracted by mix solvents of methylene chloride and ethyl acetate (150 mL × 3, methylene chloride: ethyl acetate = 4: 1). The organic layer was separated and dried by anhydrous Na$_2$SO$_4$. After concentrated in vacuum, the residue was purified by column chromatography on silica gel (methylene chloride: ethyl acetate = 10: 1) to get yellow solid of anti-parallel and parallel conformers in 44.3% (680 mg) and 45.8% (703 mg), respectively.
ap-**OH:** ^{1}H NMR (400 MHz, DMSO-d_6, ppm) δ: 2.06 (s, 6 H, -CH$_3$), 6.83 (d, J = 8.4 Hz, 4 H, phenyl-H), 7.39-7.45 (m, 4 H, benzothiophene-H), 7.54 (d, J = 8.4 Hz, 4 H, phenyl-H), 8.03 (d, J = 1.2 Hz, 2 H, benzothiophene-H), 9.54 (s, 2 H, -OH). ^{13}C NMR (100 MHz, DMSO-d_6, ppm) δ: 15.50, 115.63, 118.80, 122.35, 122.98, 126.81, 127.67, 130.61, 130.84, 136.11, 137.81, 138.09, 139.37, 147.70, 156.79, 156.97.
p-**OH:** ^{1}H NMR (400 MHz, DMSO-d_6, ppm) δ: 2.33 (s, 6 H, -CH$_3$), 6.77 (d, J = 8.8 Hz, 4 H, phenyl-H), 7.15 (dd, J_1 = 8.4 Hz, J_2 = 1.6 Hz, 2 H, phenyl-H), 7.38 (d, J = 8.4 Hz, 2 H, benzothiophene-H), 7.42 (d, J = 8.8 Hz, 4 H, phenyl-H), 7.93 (d, J = 1.6 Hz, 2 H, benzothiophene-H), 9.50 (s, 2 H, -OH).

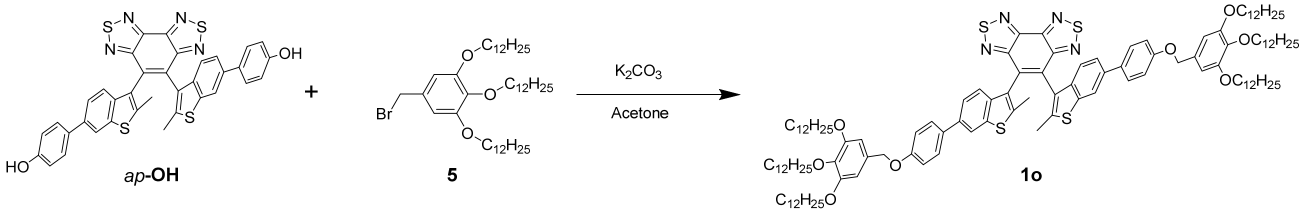

Scheme S4. Synthetic route to **1o**

Synthesis of 1o: To a 250 mL single necked bottle, *ap*-**OH** (680 mg, 1.01 mmol), **5** (3.7 g, 5.05 mmol), K$_2$CO$_3$ (1.1 g, 8 mmol) and acetone (180 mL) were added. The mixture was heated to 70°C for 9 h in the dark, then it was cooled to room temperature and concentrated in vacuum. The residue was poured into 200 mL water and extracted with methylene chloride (100 mL × 3). The organic layer was separated and washed by brine solution, dried by anhydrous Na$_2$SO$_4$. After concentrated in vacuum, the residue was purified by column chromatography on silica gel (methylene

chloride: petrol ether = 3: 1) to afford yellow solid in 76.1% (1.5 g).

1o: ^{1}H NMR (400 MHz, THF-d_8, ppm) δ: 0.76-0.80 (m, 18 H, -CH_3), 1.19-1.24 (m, 96 H, -CH_2-), 1.37-1.42 (m, 12 H), 1.65-1.71 (m, 12 H), 1.96 (s, 6 H, -CH_3), 3.80 (t, J = 6.4 Hz, 4 H, -CH_2O-), 3.86 (t, J = 6.4 Hz, 8 H, -CH_2O-), 4.89 (s, 4 H, -OCH_2-), 6.59 (s, 4 H, phenyl-H), 6.93 (d, J = 8.4 Hz, 4 H, phenyl-H), 7.19 (d, J = 8.4 Hz, 2 H, benzothiophene-H), 7.32 (dd, J_1 = 8.4 Hz, J_2 = 1.2 Hz, 2 H, benzothiophene-H), 7.49 (d, J = 8.4 Hz, 4 H, phenyl-H), 7.87 (d, J = 1.2 Hz, 2 H, benzothiophene-H). ^{13}C NMR (100 MHz, $CDCl_3$, ppm) δ: 1.02, 14.14, 15.93, 22.71, 26.11, 26.15, 29.38, 29.41, 29.43, 29.67, 29.71, 29.77, 30.35, 31.94, 69.12, 70.54, 73.44, 106.14, 115.20, 119.99, 122.71, 123.35, 126.45, 128.25, 131.48, 131.77, 133.65, 136.73, 137.95, 138.26, 138.84, 140.66, 147.68, 153.32, 156.92, 158.37. MALDI TOF (m/z) $[M+Na]^+$ Calcd. for $C_{122}H_{178}N_4O_8S_4Na$, 1978.2420; found, 1978.3240, $[M+K]^+$ Calcd. for $C_{122}H_{178}N_4O_8S_4K$, 1994.2159; found, 1994.3180.

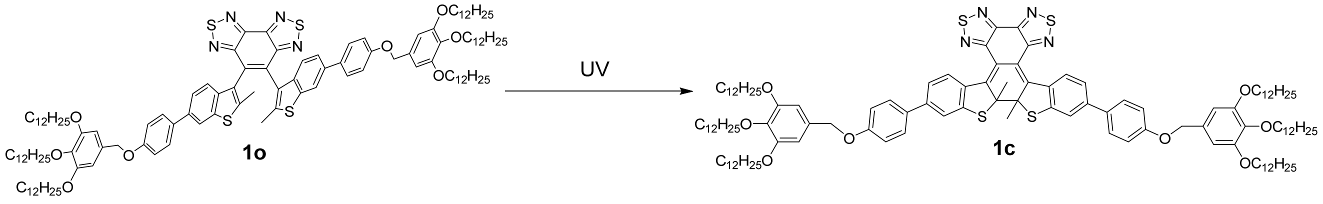

Scheme S5. Synthetic route of **1c**

Synthesis of 1c: 1o (200 mg, 0.10 mmol) was dissolved in THF (250 mL), and then irradiated by UV light (365 nm) until reach PSS. After concentration in vacuum, the residue was purified by column chromatography on silica gel (methylene chloride: petrol ether = 3: 1) to get pure **1c** of red powder in 70% (140 mg) yield and few **1o**.

^{1}H NMR (400 MHz, $CDCl_3$, ppm) δ: 0.86-0.89 (m, 18 H, -CH_3), 1.26-1.30 (m, 96 H, -CH_2-), 1.43-1.47 (m, 12 H), 1.70-1.84 (m, 12 H), 2.07 (s, 6 H, -CH_3), 3.94-4.00 (m, 12 H, -CH_2O-), 5.00 (s, 4 H, -OCH_2-), 6.64 (s, 4 H, phenyl-H), 7.06 (d, J = 8.8 Hz, 4 H, phenyl-H), 7.24 (d, J = 1.6 Hz, 2 H, benzothiophene-H), 7.48 (d, J = 1.6 Hz, 2 H, benzothiophene-H), 7.57 (d, J = 8.8 Hz, 4 H, phenyl-H), 8.08 (d, J = 8.8 Hz, 2 H, benzothiophene-H). ^{13}C NMR (100 MHz, $CDCl_3$, ppm) δ: 14.14, 14.20, 21.08, 22.71, 24.75, 26.11, 29.38, 29.43, 29.67, 29.72, 29.77, 30.34, 31.93, 53.44, 60.41, 67.16, 69.12, 70.54, 73.44, 106.13, 115.25, 118.63, 121.27, 122.47, 128.15, 130.24, 131.58, 132.35, 137.98, 144.25, 148.14, 148.47, 150.78, 153.33, 156.17, 159.12, 171.18. MALDI TOF (m/z) $[M+H]^+$ Calcd. for $C_{122}H_{179}N_4O_8S_4$, 1956.2600; found, 1956.1720, $[M+Na]^+$ Calcd. for $C_{122}H_{178}N_4O_8S_4Na$, 1978.2420, found, 1978.2317; $[M+K]^+$ Calcd. for $C_{122}H_{178}N_4O_8S_4K$, 1994.2159, found, 1994.1429.

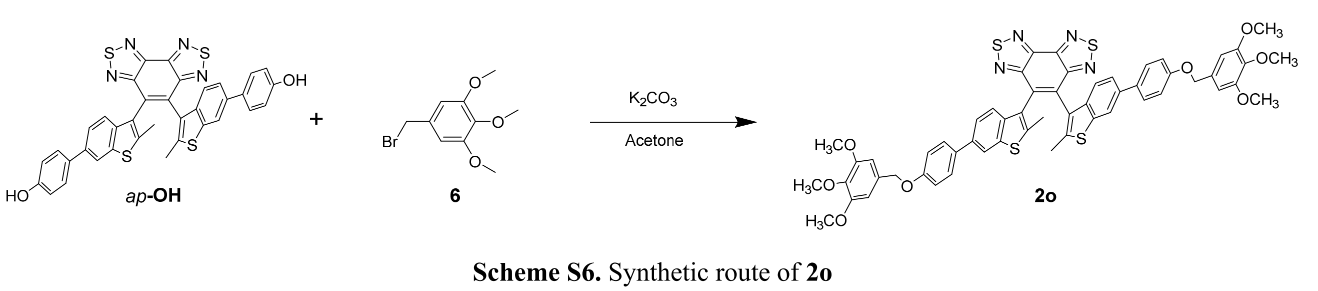

Scheme S6. Synthetic route of **2o**

Synthesis of 2o: To a 250 mL single necked bottle, *ap*-**OH** (335 mg, 0.50 mmol), **6** (783 mg, 3.0 mmol), K_2CO_3 (690 mg, 5 mmol) and acetone (90 mL) were added. The mixture was heated to 70°C for 10 h in the dark, then it was cooled to room temperature and concentrated in vacuum. The residue was poured into 200 mL water and extracted with methylene chloride (100 mL × 3). The organic layer was separated and washed by brine solution, dried by anhydrous Na_2SO_4. After concentrated in vacuum, the residue was purified by column chromatography on silica gel

(methylene chloride: petrol ether = 3: 1) to afford yellow solid in 72.5% (373 mg).

2o: ^{1}H NMR (400 MHz, $CDCl_3$, ppm) δ: 1.98 (s, 6 H, $-CH_3$), 3.86 (s, 6 H, $-CH_3$), 3.89 (s, 12 H, $-CH_3$), 5.04 (s, 4 H, $-OCH_2-$), 6.69 (s, 4 H, phenyl-H), 7.07 (d, J = 9.2 Hz, 4 H, phenyl-H), 7.16 (d, J = 8.4 Hz, 2 H, benzothiophene-H), 7.42 (dd, J_1 = 8.4 Hz, J_2 = 1.6 Hz, 2 H, benzothiophene-H), 7.58 (d, J = 9.2 Hz, 4 H, phenyl-H), 7.93 (d, J = 1.6 Hz, 2 H, benzothiophene-H). ^{13}C NMR (100 MHz, $CDCl_3$, ppm) δ: 15.92, 56.10, 60.83, 70.44, 104.63, 115.17, 115.24, 120.05, 122.70, 123.32, 126.49, 128.27, 128.35, 131.48, 132.47, 133.79, 136.68, 137.71, 138.30, 138.85, 140.71, 147.70, 153.47, 156.93, 158.28. ESI TOF (*m/z*) $[M+Na]^+$ Calcd. for $C_{56}H_{46}N_4O_8S_4Na$, 1053.2091; found, 1053.2073.

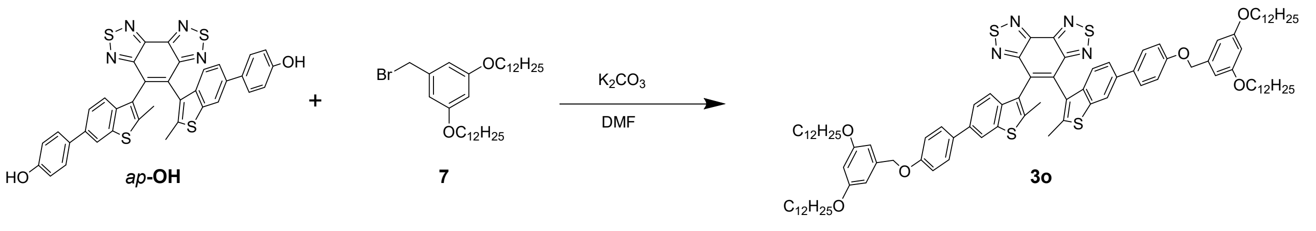

Scheme S7. Synthetic route of **3o**

Synthesis of 3o: To a 250 mL single necked bottle, *ap*-**OH** (335 mg, 0.50 mmol), **7** (1.62 g, 3.0 mmol), K_2CO_3 (690 mg, 5 mmol) and *N*,*N*-dimethylformamide (90 mL) were added. The mixture was heated to 90°C for 10 h in the dark, then it was cooled to room temperature and concentrated in vacuum. The residue was poured into 200 mL water and extracted with methylene chloride (100 mL × 3). The organic layer was separated and washed by brine solution, dried by anhydrous Na_2SO_4. After concentrated in vacuum, the residue was purified by column chromatography on silica gel (methylene chloride: petrol ether = 2: 1) to afford yellow solid in 68.5% (544 mg).

3o: ^{1}H NMR (400 MHz, $CDCl_3$, ppm) δ: 0.87 (t, J = 6.8 Hz, 12H, $-CH_3$), 1.26 (m, 64 H), 1.42-1.44 (m, 8 H), 1.73-1.80 (m, 8 H), 1.96 (s, 6 H, $-CH_3$), 3.94 (t, J = 6.4 Hz, 8 H, $-OCH_2-$), 5.03 (s, 4 H, $-OCH_2-$), 6.41 (s, 2 H, phenyl-H), 6.58 (d, J = 2.0 Hz, 4 H, phenyl-H), 7.04 (d, J = 8.4 Hz, 4 H, phenyl-H), 7.15 (d, J = 8.4 Hz, 2 H, benzothiophene-H), 7.41 (d, J = 8.8 Hz, 2 H, benzothiophene-H), 7.55 (d, J = 8.4 Hz, 4 H, phenyl-H), 7.92 (s, 2 H, benzothiophene-H). ^{13}C NMR (100 MHz, $CDCl_3$, ppm):δ 14.16, 15.93, 22.72, 26.08, 29.28, 29.38, 29.43, 29.61, 29.63, 29.66, 29.69, 31.94, 68.10, 70.15, 100.76, 105.66, 115.24, 120.00, 122.71, 123.38, 126.46, 128.27, 131.51, 133.64, 136.77, 138.27, 138.85, 139.14, 140.66, 147.70, 156.94, 158.34, 160.56. (ESI positive ion mode for $[M+K]^+$): Calcd for $C_{98}H_{130}O_6N_4S_4K$, 1625.8505; found, 1625.8534

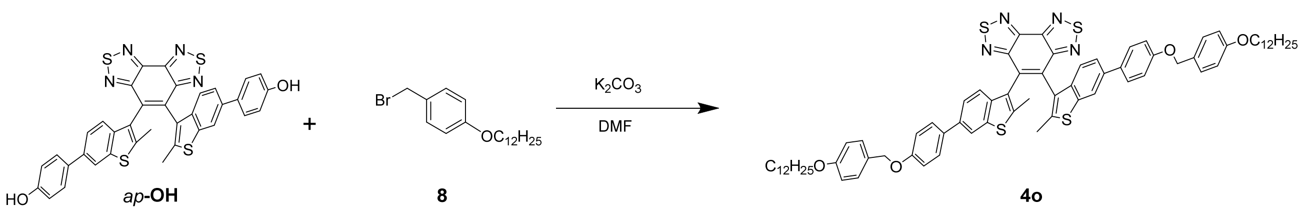

Scheme S8. Synthetic route of **4o**

Synthesis of 4o: To a 250 mL single necked bottle, *ap*-**OH** (335 mg, 0.50 mmol), **8** (1.07 g, 3.0 mmol), K_2CO_3 (690 mg, 5 mmol) and *N*,*N*-dimethylformamide (90 mL) were added. The mixture was heated to 90°C for 10 h in the dark, then it was cooled to room temperature and concentrated in vacuum. The residue was poured into 200 mL water and extracted with methylene chloride (150 mL × 3). The organic layer was separated and washed by brine solution, dried by anhydrous Na_2SO_4. After concentrated in vacuum, the residue was recrystallized in tetrahydrofuran to afford yellow solid in 50.2% (306 mg).

4o: ^{1}H NMR (400 MHz, $CDCl_3$, ppm) δ: 0.86-0.88 (m, 6 H, $-CH_3$), 1.27 (m, 32 H), 1.45 (m, 4 H), 1.75-1.80 (m, 4 H), 1.97 (s, 6 H, $-CH_3$), 3.96 (t, J = 6.4 Hz, 4 H, $-OCH_2-$), 5.03 (s, 4 H, $-OCH_2-$), 6.92 (t, J = 7.6 Hz, 4 H, phenyl-H), 7.05 (d, J = 7.6 Hz, 4 H, phenyl-H), 7.15 (d, J = 8.0 Hz, 2 H, benzothiophene-H), 7.37 (d, J = 8.0 Hz, 4 H, phenyl-H), 7.42 (d, J = 8.4 Hz, 2 H, benzothiophene-H), 7.56 (d, J = 7.6 Hz, 4 H, phenyl-H), 7.92 (s, 2 H, benzothiophene-H). (ESI positive ion mode for $[M+K]^+$): Calcd for $C_{74}H_{82}O_4N_4S_4K$, 1257.4850; found, 1257.4811.

Full Enantiomer Separation of 1o-3o

A pair of open-form enantiomers (*P*-**1o** and *M*-**1o**) were separated from the racemic **1o** by a preparative HPLC equipped with CHIRALCEL® Chiralpak ID (50 diameter × 250 mm, eluent: methylene chloride/n-hexane = 45/55, v/v). Another pair of closed-form enantiomers [(*R*,*R*)-**1c** and (*S*,*S*)-**1c**] were obtained by irradiating *P*-**1o** and *M*-**1o** with UV light (365 nm) in THF until reaching PSS. After concentration in vacuum, the residue was purified by column chromatography on silica gel (methylene chloride/n-hexane = 45/55, v/v) to get pure (*R*,*R*)-**1c** and (*S*,*S*)-**1c**, respectively. Similarly, *P*-**2o** and *M*-**2o** were separated by CHIRALCEL® Chiralpak IK (50 diameter × 250 mm, eluent: methylene chloride: n-hexane = 70/30, v/v). *P*-**3o** and *M*-**3o** were separated by CHIRALCEL® Chiralpak IK (50 diameter × 250 mm, eluent: methylene chloride/n-hexane = 60/40, v/v). However, due to very poor solubility of **4o**, it failed to be separated by preparative HPLC.

P-**1o** : ^{1}H NMR (400 MHz, $CDCl_3$, ppm) δ: 0.86-0.89 (m, 18 H, $-CH_3$), 1.26 (m, 96 H, $-CH_2-$), 1.43-1.47 (m, 12 H), 1.71-1.83 (m, 12 H), 1.97 (s, 6 H, $-CH_3$), 3.93-4.00 (m, 12 H, $-CH_2O-$), 4.99 (s, 4 H, $-OCH_2-$), 6.64 (s, 4 H, phenyl-H), 7.05 (d, J = 8.8 Hz, 4 H, phenyl-H), 7.16 (d, J = 8.4 Hz, 2 H, benzothiophene-H), 7.42 (dd, J_1 = 8.4 Hz, J_2 = 1.2 Hz, 2 H, benzothiophene-H), 7.57 (d, J = 8.4 Hz, 4 H, phenyl-H), 7.92 (d, J = 1.2 Hz, 2 H, benzothiophene-H).

M-**1o:** ^{1}H NMR (400 MHz, $CDCl_3$, ppm) δ: 0.86-0.89 (m, 18 H, $-CH_3$), 1.26 (m, 96 H, $-CH_2-$), 1.43-1.47 (m, 12 H), 1.71-1.83 (m, 12 H), 1.97 (s, 6 H, $-CH_3$), 3.93-4.00 (m, 12 H, $-CH_2O-$), 4.99 (s, 4 H, $-OCH_2-$), 6.64 (s, 4 H, phenyl-H), 7.05 (d, J = 8.8 Hz, 4 H, phenyl-H), 7.16 (d, J = 8.4 Hz, 2 H, benzothiophene-H), 7.42 (dd, J_1 = 8.4 Hz, J_2 = 1.2 Hz, 2 H, benzothiophene-H), 7.57 (d, J = 8.4 Hz, 4 H, phenyl-H), 7.92 (d, J = 1.2 Hz, 2 H, benzothiophene-H).

(*R*,*R*)-**1c:** ^{1}H NMR (400 MHz, $CDCl_3$, ppm) δ: 0.86-0.89 (m, 18 H, $-CH_3$), 1.26 (s, 96 H, $-CH_2-$), 1.47 (s, 12 H), 1.71-1.81 (m, 12 H), 2.07 (s, 6 H, $-CH_3$), 3.94-4.00 (m, 12 H, $-CH_2O-$), 5.00 (s, 4 H, $-OCH_2-$), 6.64 (s, 4 H, phenyl-H), 7.06 (d, J = 8.8 Hz, 4 H, phenyl-H), 7.24 (s, 2 H, benzothiophene-H), 7.48 (d, J = 1.6 Hz, 2 H, benzothiophene-H), 7.57 (d, J = 8.8 Hz, 4 H, phenyl-H), 8.09 (d, J = 8.8 Hz, 2 H, benzothiophene-H).

(*S*,*S*)-**1c:** ^{1}H NMR (400 MHz, $CDCl_3$, ppm) δ: 0.86-0.89 (m, 18 H, $-CH_3$), 1.26 (s, 96 H, $-CH_2-$), 1.43-1.47 (m, 12 H), 1.71-1.84 (m, 12 H), 2.07 (s, 6 H, $-CH_3$), 3.94-4.00 (m, 12 H, $-CH_2O-$), 5.00 (s, 4 H, $-OCH_2-$), 6.64 (s, 4 H, phenyl-H), 7.06 (d, J = 8.8 Hz, 4 H, phenyl-H), 7.24 (d, J = 1.6 Hz, 2 H, benzothiophene-H), 7.48 (s, 2 H, benzothiophene-H), 7.57 (d, J = 8.4 Hz, 4 H, phenyl-H), 8.09 (d, J = 8.8 Hz, 2 H, benzothiophene-H).

P-**2o** : ^{1}H NMR (400 MHz, $CDCl_3$, ppm) δ: 1.98 (s, 6 H, $-CH_3$), 3.86 (s, 6 H, $-CH_3$), 3.89 (s, 12 H, $-CH_3$), 5.04 (s, 4 H, $-OCH_2-$), 6.69 (s, 4 H, phenyl-H), 7.07 (d, J = 8.8 Hz, 4 H, phenyl-H), 7.16 (d, J = 8.4 Hz, 2 H, benzothiophene-H), 7.42 (dd, J_1 = 8.4 Hz, J_2 = 1.6 Hz, 2 H, benzothiophene-H), 7.58 (d, J = 8.8 Hz, 4 H, phenyl-H), 7.93 (d, J = 1.6 Hz, 2 H, benzothiophene-H). ESI TOF (*m/z*) $[M+Na]^+$ Calcd. for $C_{56}H_{46}N_4O_8S_4Na$, 1053.2091; found, 1053.2074.

M-**2o** : ^{1}H NMR (400 MHz, $CDCl_3$, ppm) δ: 1.98 (s, 6 H, $-CH_3$), 3.86 (s, 6 H, $-CH_3$), 3.89 (s, 12 H, $-CH_3$), 5.04 (s, 4 H, $-OCH_2-$), 6.69 (s, 4 H, phenyl-H), 7.07 (d, J = 8.8 Hz, 4 H, phenyl-H), 7.16 (d, J = 8.4 Hz, 2 H, benzothiophene-H), 7.42 (dd, J_1 = 8.4 Hz, J_2 = 1.6 Hz, 2 H, benzothiophene-H), 7.58 (d, J = 8.8 Hz, 4 H, phenyl-H), 7.93 (d, J = 1.6 Hz, 2 H, benzothiophene-H). ESI TOF (*m/z*) $[M+Na]^+$ Calcd. for $C_{56}H_{46}N_4O_8S_4Na$, 1053.2091; found, 1053.2103.

P-**3o:** ^{1}H NMR (400 MHz, $CDCl_3$, ppm) δ: 0.87 (t, J = 6.8 Hz, 12 H, $-CH_3$), 1.26 (m, 64 H), 1.42-1.44 (m, 8 H), 1.73-

1.80 (m, 8 H), 1.96 (s, 6 H, -CH_3), 3.94 (t, *J* = 6.4 Hz, 8 H, -OCH_2-), 5.03 (s, 4 H, -OCH_2-), 6.41 (s, 2 H, phenyl-H), 6.58 (d, *J* = 1.6 Hz, 4 H, phenyl-H), 7.04 (d, *J* = 8.8 Hz, 4 H, phenyl-H), 7.15 (d, *J* = 8.4 Hz, 2 H, benzothiophene-H), 7.41 (d, *J* = 8.4 Hz, 2 H, benzothiophene-H), 7.55 (d, *J* = 8.4 Hz, 4 H, phenyl-H), 7.91 (s, 2 H, benzothiophene-H). (ESI positive ion mode for $[M+K]^+$): Calcd for $C_{98}H_{130}O_6N_4S_4K$, 1625.8505; found, 1625.8509.
***M*-3o:** ^1H NMR (400 MHz, $CDCl_3$, ppm) *δ*: 0.87 (t, *J* = 6.8 Hz, 12 H, -CH_3), 1.26 (m, 64 H), 1.43-1.44 (m, 8 H), 1.73-1.80 (m, 8 H), 1.96 (s, 6 H, -CH_3), 3.94 (t, *J* = 6.4 Hz, 8 H, -OCH_2-), 5.03 (s, 4 H, -OCH_2-), 6.41 (s, 2 H, phenyl-H), 6.58 (d, *J* = 2.0 Hz, 4 H, phenyl-H), 7.04 (d, *J* = 8.8 Hz, 4 H, phenyl-H), 7.15 (d, *J* = 8.4 Hz, 2 H, benzothiophene-H), 7.40-7.42 (m, 2 H, benzothiophene-H), 7.55 (d, *J* = 8.8 Hz, 4 H, phenyl-H), 7.91 (s, 2 H, benzothiophene-H). (ESI positive ion mode for $[M+K]^+$): Calcd for $C_{98}H_{130}O_6N_4S_4K$, 1625.8505; found, 1625.8509.

1.4 Calculation formula of Manipulation Robustness (R_P)

The chiral photoswitch with a large ΔHTP indicates that the photoswitch can induce large manipulation range of reflection spectra. Lower concentration of photoswitch means higher HTP value, which will efficiently prevent large number of defects in LC texture. The long thermal stability time of the chiral photoswitch illustrates the high stability of the soft helix at any intermediate state. Fast average shift rate of the reflection spectra shows the fast-transforming response to light. The calculation is written as:

$$R_{\mathrm{p}} = e^{t}\ \Delta\,\mathrm{HTP}\ c^{-1} v$$

where *t* is thermal stability time of the chiral photoswitch in soft helix, *v* is the average shift rate of the reflection band, *ΔHTP* is the variation of HTP value before and after irradiation, and *c* is the molar ratio of photoswitch to mesogen.

We constructed the formula by multiplying several parameters. The reasons are as following: (1) The above four parameters represent different performance of chiral photoswitch in liquid crystal, which are highly interrelated. So, we express the formula through multiplication of its terms. (2) To eliminate interference from units, we construct the photoresponsive Robustness as a dimensionless value by multiplying several parameters, which is easy to be compared and understand without the influence of units.

The explanation for the adoption of e^t and c^{-1}: Generally, some chiral photoswitches are not thermal stable, so the stability time denote as 0. To prevent the final equation result from being 0, we express it in the form of an exponent. We adopted the Natural Index because it aligns with the mathematical structure used to model the thermal attenuation dynamics of photoswitches: $e^{k\tau} = (A_0 - A_\infty)/(A - A_\infty)$. In addition, the concentration of chiral photoswitch is inversely proportional to the helical twisting power (HTP) by the formula of $\beta = (\mathrm{P}\, c)^{-1}$, in which β is the helical twisting power, P is the helical pitch and *c* is the concentration of chiral photoswitch. So, we adopt the reciprocal of concentration to present HTP, and lower concentration of photoswitch means higher HTP value, which will efficiently prevent the defects in LC textures.

S1.5 Principle of the liquid crystal Q-plate and Damman vortex grating

Part 1: Liquid Crystal Q-plate

The Q-plate is an optical diffraction element (ODE) made of LC material. The Q-plate introduces a spatially varying birefringence, with the birefringence axis orientation:

$$\alpha(r,\varphi) = q\varphi + \alpha_0$$

Where *r* is the radius, *q* is the topological charge of the Q-plate, φ is the azimuthal angle, and α_0 is the initial angle when φ=0. Here, the $\alpha_0 = 0$. The Q-plate can convert circularly polarized light into an optical vortex (topological charge *m*=2*q*) and convert linearly polarized light into a vector beam (polarization order *P*=2*q*). The principle can be described by Jones matrix method.

The action of the Q-plate on the polarization state can be represented by a Jones matrix:

$$J\_Q = e^{i\Gamma}\begin{pmatrix} \cos 2\alpha & \sin 2\alpha \\ \sin 2\alpha & -\cos 2\alpha \end{pmatrix}$$

Where Γ is the optical retardation induced by the Q-plate. When the retardation of the Q-plate meets the half-wave condition, the Q-plate can be normalized by:

$$J\_Q = \begin{pmatrix} \cos 2\alpha & \sin 2\alpha \\ \sin 2\alpha & -\cos 2\alpha \end{pmatrix}$$

Part 2: Generation of a Vortex Beam

When an ordinary right-hand circular polarized light beam (RCP) is reflected by a Q-plate with a topological charge *q*, it converts into a vortex beam with a topological charge *m* = 2*q*, meaning the phase of the beam undergoes two complete rotations (2π phase change) around its center as it propagates. The polarization conversion and the corresponding topological charge change can be described as follows:

The Jones vector for the incident RCP light can be expressed as:

$$E_{in} = \frac{1}{\sqrt{2}}\begin{pmatrix} 1 \\ i \end{pmatrix}$$

The output light can be calculated by:

$$E_{out} = J\_Q \cdot E_{in}$$

$$E_{out} = \frac{1}{\sqrt{2}}\begin{pmatrix} e^{i2q\varphi} \\ e^{i2q\varphi+\pi} \end{pmatrix}$$

After passing through the Q-plate, the resulting beam has a spatially varying phase structure. This results in a vortex beam with a helical wavefront and an azimuthal phase dependence of $e^{i2q\varphi}$.

Part 3: Generation of a Vector Beam

When linearly polarized (LP) light beam is incident on the same Q-plate, the spatially varying birefringence leads to the generation of a vector beam, characterized by spatially varying polarization across the beam profile. Unlike scalar beams, the polarization in a vector beam changes as a function of the azimuthal angle φ, creating polarization patterns

such as radial or azimuthal polarization.

The Jones vector for the linearly light along the x-axis can be expressed as:

$$E_{in} = \begin{pmatrix} 1 \\ 0 \end{pmatrix}$$

The output light can be calculated by:

$$E_{out} = J\_Q \cdot E_{in}$$

$$E_{out} = \frac{1}{\sqrt{2}} \begin{pmatrix} \cos 2q\varphi \\ \sin 2q\varphi \end{pmatrix}$$

The resulting beam exhibits a polarization distribution that depends on the azimuthal angle φ. Specifically, if q = 1, the resulting polarization is either radially or azimuthally polarized. The beam does not carry a phase singularity like the vortex beam, but instead shows a spatially varying polarization state, making it a vector beam.

Part 4: Principle of designing the 3×3 CLC DVG

We used the approach inherited from the geometric phase structure of Dammann vortex grating (DVG) configured with cholesteric liquid crystals (CLCs). Generally, DVG induces a diffraction array with equal-intensity orders, with each order imposed a certain OAM. In principle, the spatial phase distribution $\psi(x,y)$ of a two-dimensional DVG can be expressed as:

$$\begin{aligned} \exp[i\psi(x,y)] &= \sum_{N_x}\sum_{N_y} A_{N_xN_y} \exp\left[iN_x\left(k_x x + \Delta l_x \phi\right) + iN_y\left(k_y y + \Delta l_y \phi\right)\right] \\ &= \sum_{N_x}\sum_{N_y} A_{N_xN_y} \exp\left[i\left(N_x k_x x + N_y k_y y\right)\right]\exp\left[i\left(N_x \Delta l_x + N_y \Delta l_y\right)\phi\right] \\ &= \sum_{N_x}\sum_{N_y} A_{N_xN_y} \exp\left[i\left(N_x k_x x + N_y k_y y\right)\right]\exp\left[i l_{xy}\phi\right] \end{aligned} \quad (1)$$

where N_x and N_y are the diffraction ordinals in the x and y dimensions; $\left|A_{N_xN_y}\right|^2$ is the energy of the labelled (N_x, N_y) order normalized to the total intensity; angle ϕ is defined as $\phi = \arctan(y/x)$. Equation (1) indicates a $n_x \times n_y$ diffraction array (n_x and n_y are the count of N_x and N_y respectively) where each diffraction order propagates along the wavevector $\vec{k}$ of (N_xk_x, N_yk_y), and l_{xy} is the imposed topological charge (TC) expressed as $l_{xy} = N_x\Delta l_x + N_y\Delta l_y$, where Δl_x and Δl_y are the interval of TCs between two neighboring diffraction orders corresponding to x and y dimensions. In our experiment, the 3×3 CLC DVG with the parameters as $\Delta l_x = \Delta l_y = 1$ and N_x' & $N_y \in \{-1, 0, +1\}$ is fabricated so that the imposed TC (l_{xy}) at the (N_x, N_y) diffraction order conforms to $l_{xy}' = N_x + N_y$. To configure exp [$i\psi(x, y)$] in the CLC element, the ideal phase distributions as exp [$i\psi(x, y)$] are judiciously approximated from the typical binary DVG with the alternate phase distribution between 0 and π. As the expected phase profile is mapped into the azimuthal angle distribution of CLCs following the principle of geometric phase, a particular dynamic photopatterning process based on the programmable digital mirror device (DMD) is implemented to fabricate the tailored CLCs.

Supplementary S1.6 Supplementary information for photo-programmable infrared beam

When the 1550- or 1310-nm laser is impinged on the Q-plate at nematic phase during the photo-inversion process, we can generate the vector beam, showing a distinct donut-like beam spot with a centered polarization singularity

(Fig. 4c-i, Supplementary Fig. S61, Supplementary Video S6 and Video S7). Besides, the cross-like intensity profiles after transmission through the analyzer further confirms the inhomogeneous polarization distribution of vector beam with polarization topological charge of 2 (Fig. 4c-ii and 4c-iii, Supplementary Video S6 and Video S7). Such a vector beam can be manipulated by photo irradiation, exhibiting the switching between simple Gaussian beam and vector beam at the transmitted end (Supplementary Video S6 and Video S7).

Given the dynamic modulating on vortex beam, we can further realize the complex programming on infrared optical field, such as through Dammann vortex grating (DVG) to generate the vortex array. Figure 4d-i depicts the photoresponsive chiral invertible LC system encoded with DVG pattern, which is consistent with the DVG pattern in Fig. 4d-i. When the PBG located at corresponding wavelength of 1550 nm, the vortex array with 3 × 3 donut-like reflected diffraction orders (n_x, n_y) carry topological charge $m = n_x + n_y$ (corresponding to orbital angular moment range of $-2 - +2$) were observed, whose radius was proportional to topological charge $|m|$ (Fig. 4d-ii). The numbers and tilt directions of dark stripes after a cylindrical lens further prove the properties (Fig. 4d-iii). The vortex array beams also showed reversible photo-manipulation properties by UV and visible light (Fig. 4d-ii and 4d-iii).

S1.7 Encoding rule based on the change of diffraction pattern

The encoding rule of the spatiotemporal coding system is demonstrated as follows. In Fig. 5c, as exampled by 1550-nm laser, when the reflection band of photoresponsive LCs with right-handed helix at 1550 nm, it reflected right-handed CP-light and showed a polarization splitting at left side, which was denoted as “1”. Upon irradiation by UV light, the reflection band shifted to other wavelength, only weak light was reflected and showed a weak central symmetric polarization splitting, which was denoted as “0”. After chiral inversion by further irradiation, when the reflection band located at 1550 nm again, left-handed CP-light was reflected and showed a polarization splitting at right side, denoted as “-1” (Supplementary Video S10). Similarly, vortex light of 1310 nm and Airy beam of 980 nm also showed analogue phenomena and encoding rule. Subsequently, we defined the 980-, 1310- and 1550-nm laser as the first, second and third digit, respectively (Supplementary Video S8-S10). Thus, we built up a ternary spatial coding system, corresponded to 26 English letters based on the diffraction pattern switch of three laser beams (Fig. 5c).

To further enhance the information density and coding efficiency, time evolution was introduced. We employed a 365-nm laser to excite the fluorescence of the chiral photoswitch and a light shutter to control the on and off of the fluorescence (Fig 5a). When the fluorescence is on, it was denoted as “1”, and if the fluorescence is off, it was denoted as “0”. The fluorescence signals change with time showing the time domain encoding to distinguish the upper and lower case of the 26 English characters (Fig 5b).

2. Supplementary Videos

Video S1 The reflection color change of the oily-streak texture in extremely broad range

Upon exposure to a UV light (365 nm), the photoresponsive helical LC exhibited a continuous and fast change of reflection color from initial dark blue, passing through blue, green, red up to almost black during the process of reflection band shift from 400 to 3000 nm.

Video S2 The reflection color change of the oily-streak texture during the process of the chirality inversion

Upon exposure to a UV light (365 nm), the photoresponsive helical LC exhibited reflection color of brown with rare change at right-handedness helix. When the helical superstructure changed into nematic phase, it clearly shows distinct birefringent color of nematic phase. Further irradiation by UV light, the oily-streak texture appears again with dark red color, showing chiral inversion by photo irraidation.

Video S3 The POM images change of chirality inversion in homeotropic cell upon irradiated by UV light

The photoresponsive helical LC in homeotropic cell was in a lying helix and showed distinct fingerprint optical texture in homeotropic cell. Upon irradiated by UV light, the helix finger of chiral LC changed from being border, disappearance and reappearance of the fingerprint texture, suggesting the chirality inversion of the helix of the LC.

Video S4 The change of reflected vortex beam and transmission pattern passing through cylindrical lens detected by 1550 nm laser after reflected by geometric phase of Q-plate based on photoswitchable chiral invertible LCs

A vortex diffraction showing donut-like pattern was generated when the reflection band located at 1550 nm in a right-handed helix. The topological charge was confirmed by a cylindrical lens. The vortex beam and topological charge can be modulated by UV and visible light reversibly.

Video S5 The change of reflected vortex beam and transmission pattern passing through cylindrical lens detected by 1310 nm laser after reflected by geometric phase of Q-plate based on photoswitchable chiral invertible LCs

A vortex diffraction detected by 1310-nm laser showed similar change as detected by 1550-nm laser.

Video S6 The change of vector beam and transmission pattern passing through polarizer detected by 1550 nm laser after transmission through Q-plate based on photoswitchable chiral invertible LCs

When the photoreponsive LCs was in the nematic phase, the vector beam can be easily obtained detected by 1550-nm laser. The cross-like intensity profiles after transmission through the polarizer can also be observed. The vector vortex beam can be modulated by UV and visible light reversibly.

Video S7 The change of vector beam and transmission pattern passing through polarizer detected by 1310 nm laser after transmission through Q-plate based on photoswitchable chiral invertible LCs

When the photoreponsive LCs was in the nematic phase, the vector beam can be easily obtained detected by 1310-nm laser. The cross-like intensity profiles after transmission through the polarizer can also be observed. The vector vortex beam can be modulated by UV and visible light reversibly.

Video S8 The change of Airy beam detected by 980 nm laser after reflected by the photoswitchable chiral invertible LCs

At initial, the photoresponsive helical LC was in right-handed helix and the reflection band covered the wavelength of 980 nm. Half of the orthogonally Airy beams with high intensity can be seen which are composed of a main lobe and a family of satellite beamlets whose intensity decays exponentially. Upon exposure to UV light (365 nm), the reflection band left the wavelength of 980 nm, and the Airy beam displayed dual orthogonally polarized pattern with weak intensity. Further irradiation of UV light, the reflection band covered the wavelength of 980 nm again in left-handed helix. Thus, the airy beam showed opposite orthogonally polarized pattern with high intensity. Upon irradiated by visible light, it displayed opposite process of the change of Airy beams.

Video S9 The change of diffraction pattern of fork grating detected by 1310 nm laser after reflected by the photoswitchable chiral invertible LCs

At initial, the photoresponsive helical LC was in right-handed helix and the reflection band covered the wavelength of 1310 nm. It showed the high intensity of diffraction pattern of zeroth order beam spot with one donut-like optic vortex at left. Upon exposure to UV light (365 nm), the reflection band left the wavelength of 1310 nm, and the diffraction pattern displayed weak intensity of zeroth order beam spot with two donut-like optic vortex. Further irradiation of UV light, the reflection band covered the wavelength of 1310 nm again in left-handed helix. Thus, the diffraction pattern showed high intensity of zeroth order beam spot with one donut-like optic vortex at right. Upon irradiated by visible light, it displayed opposite process of the change of diffraction pattern of fork grating.

Video S10 The change of diffraction pattern of polarized grating detected by 1550 nm laser after reflected by the photoswitchable chiral invertible LCs

At initial, the photoresponsive helical LC was in right-handed helix and the reflection band covered the wavelength of 1310 nm. It showed the high intensity of diffraction pattern of two beam spots at left. Upon exposure to UV light (365 nm), the reflection band left the wavelength of 1550 nm, and the diffraction pattern displayed weak intensity of three beam spots with the zeroth order and ± 1st order diffraction. Further irradiation of UV light, the reflection band covered the wavelength of 1550 nm again in left-handed helix. Thus, the diffraction pattern showed high intensity of diffraction pattern of two beam spots at right. Upon irradiated by visible light, it displayed opposite process of the change of diffraction pattern of polarized grating.

Video S11 The information encoding based on the diffraction pattern change of 980, 1310 and 1550 nm and corresponding encoding words

The encoding process of a series of words of "Ecust–Photo" based on the different diffraction pattern changes of 980, 1310 and 1550 nm.

Video S12 The exhibition of the demo devices and reversible change of the pattern induced by UV and visible light.

We construct a demo device which can achieve the information encoding based on the reversible diffraction pattern change. We also exhibit the reversible change of the pattern on the screen by irradiation of UV and visible light.

3. Supplementary Figures

Characterization

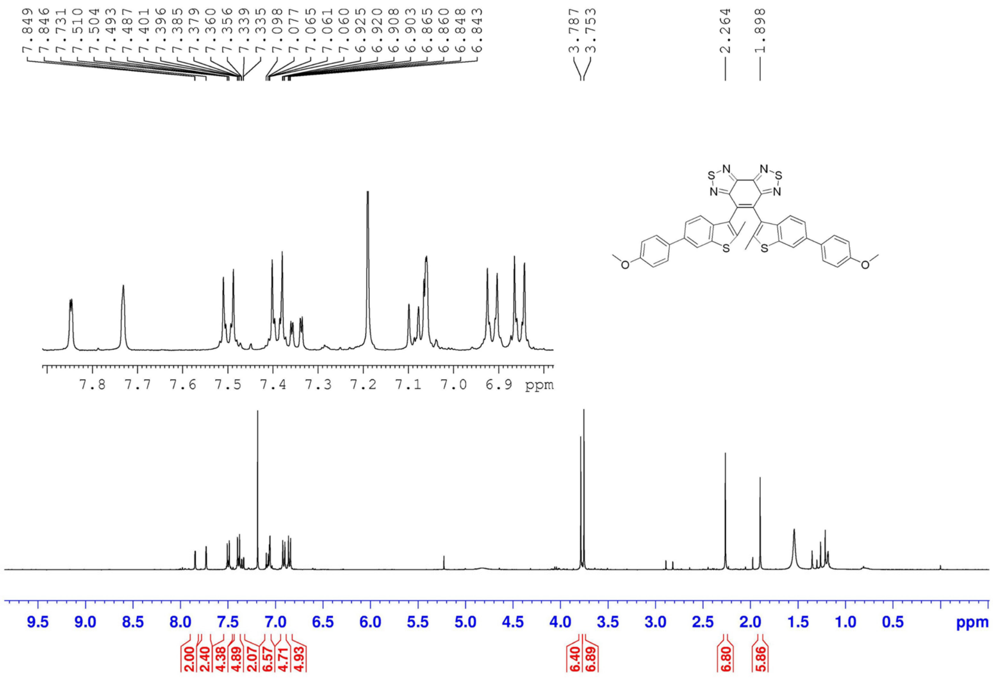

Figure S1. ^{1}H NMR spectrum of compound **BBTE-OCH$_3$** in $CDCl_3$.

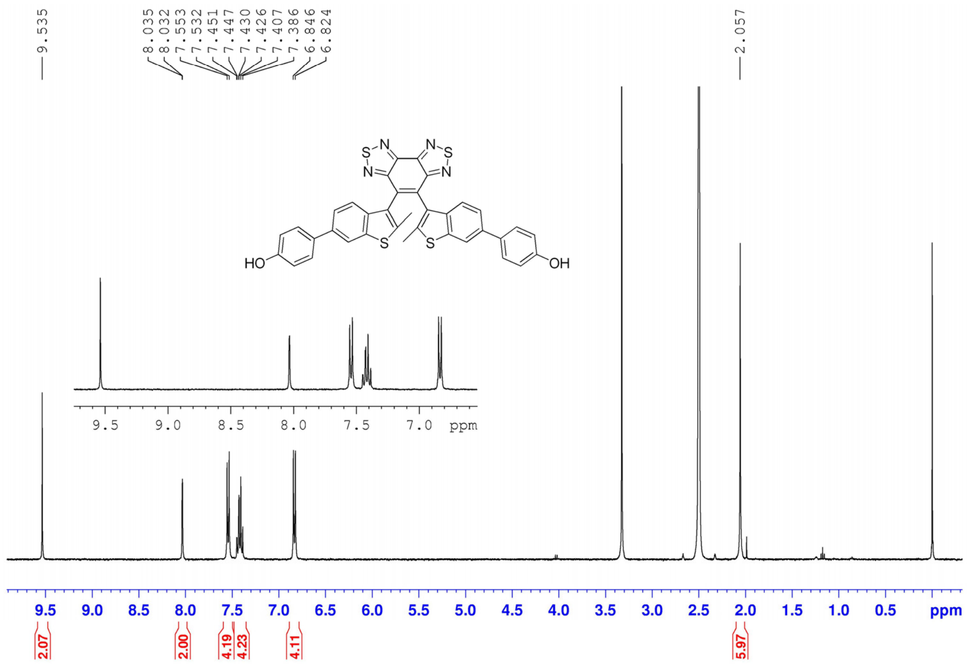

Figure S2. ^{1}H NMR spectrum of compound ***ap*-OH** in DMSO-d_6.

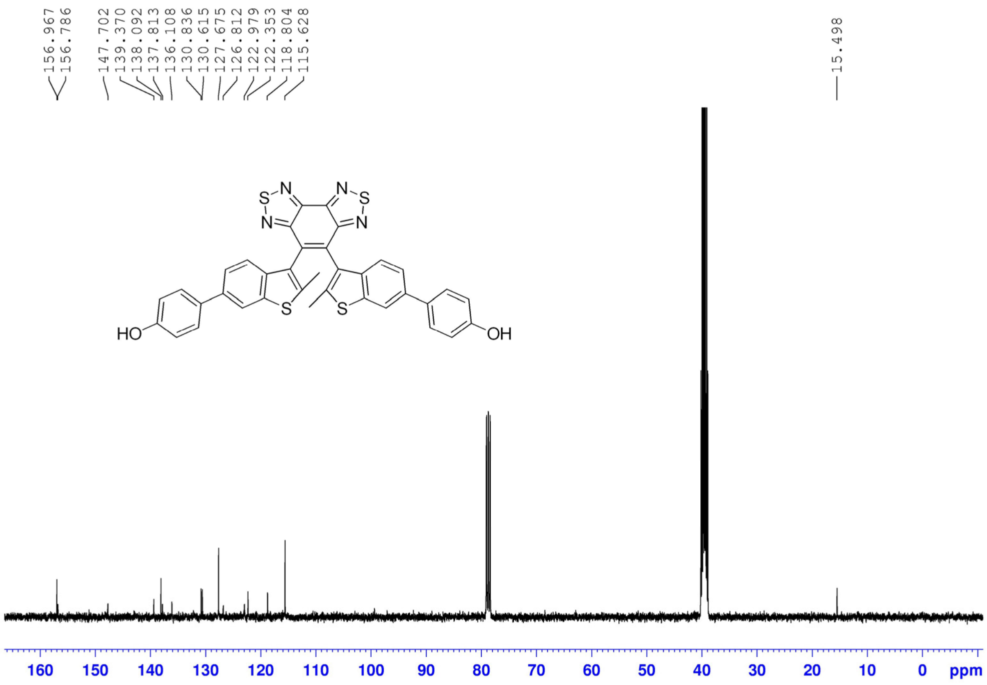

Figure S3. ^{13}C NMR spectrum of ***ap*-OH** in DMSO-d_6.

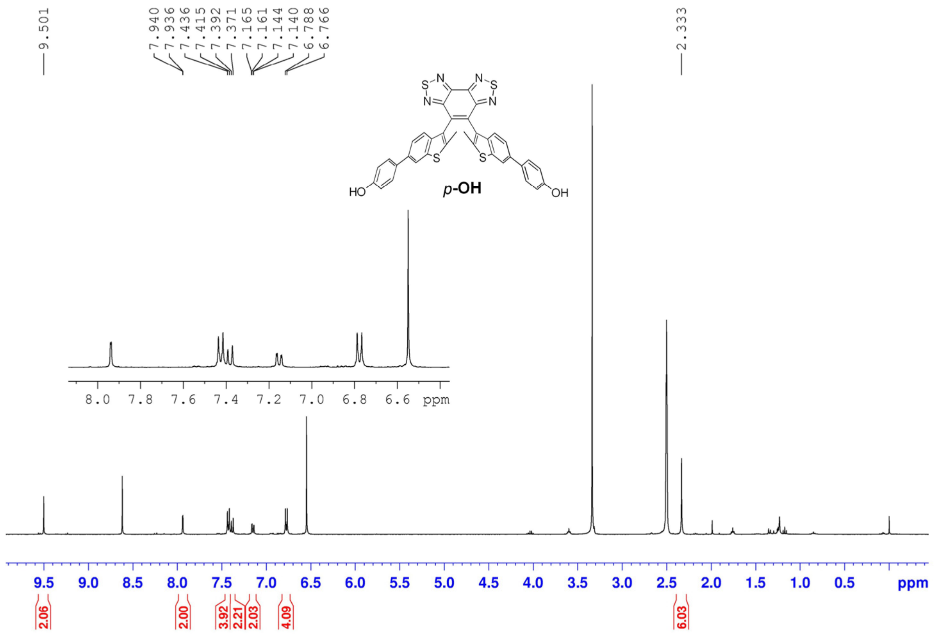

Figure S4. ^{1}H NMR spectrum of compound ***p*-OH** in DMSO-d_6**.**

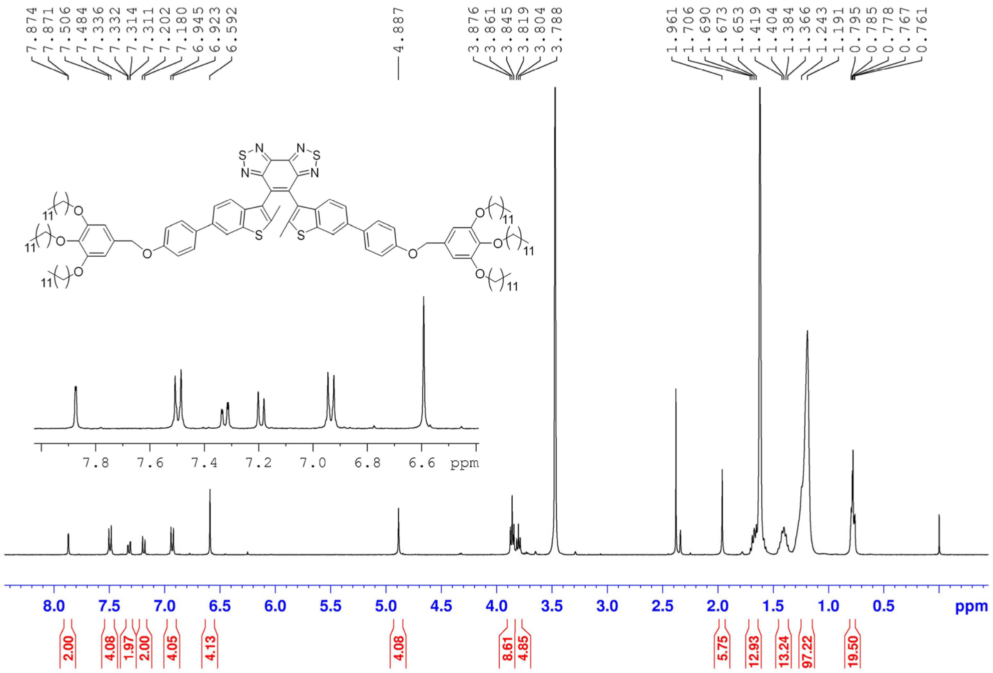

Figure S5. ^{1}H NMR spectrum of compound **1o** in THF-d_8.

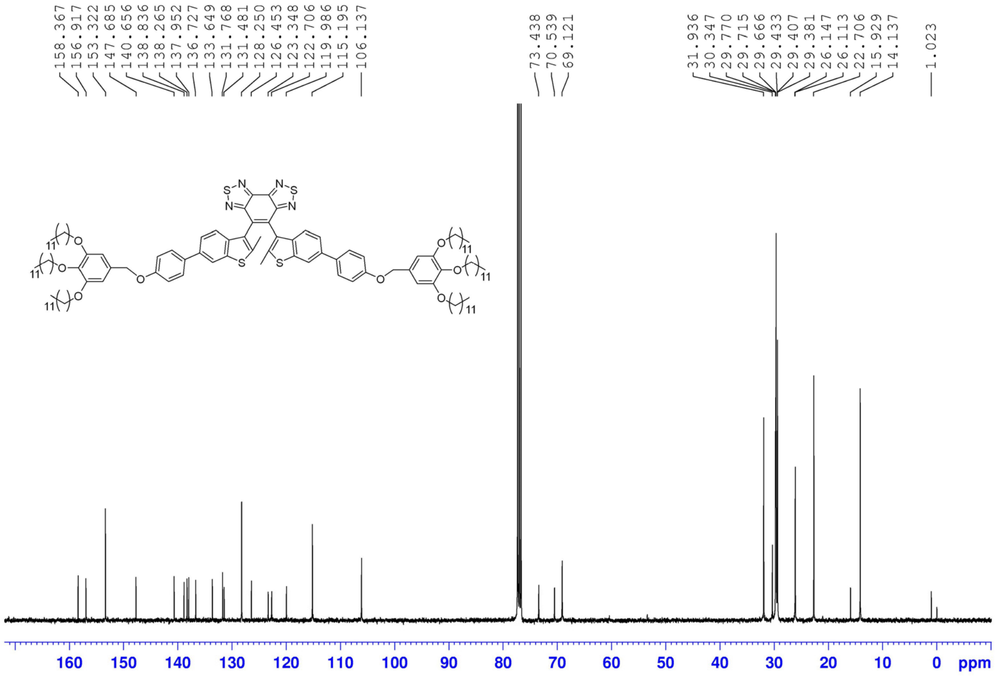

Figure S6. ^{13}C NMR spectrum of **1o** in $CDCl_3$.

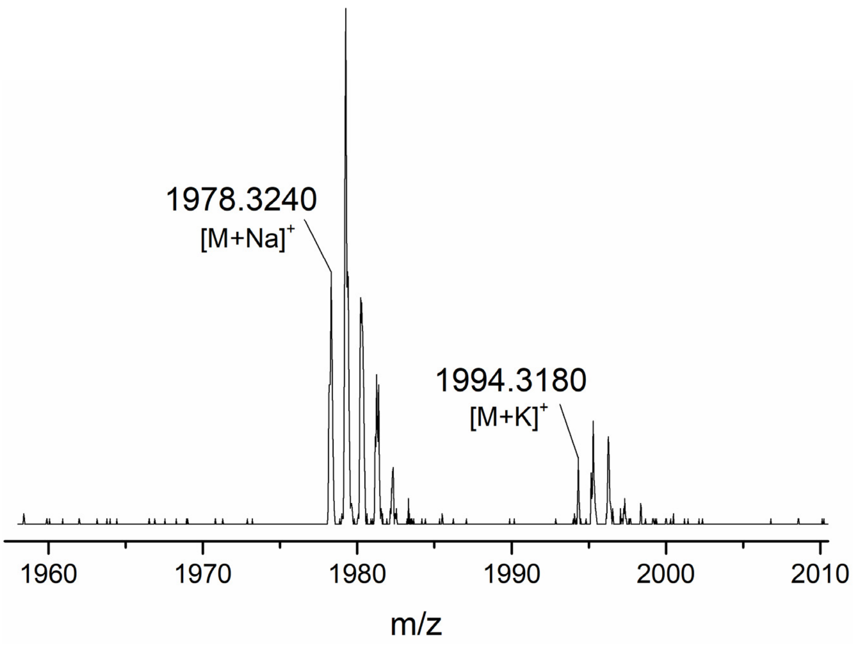

Figure S7. Mass spectrum of **1o**.

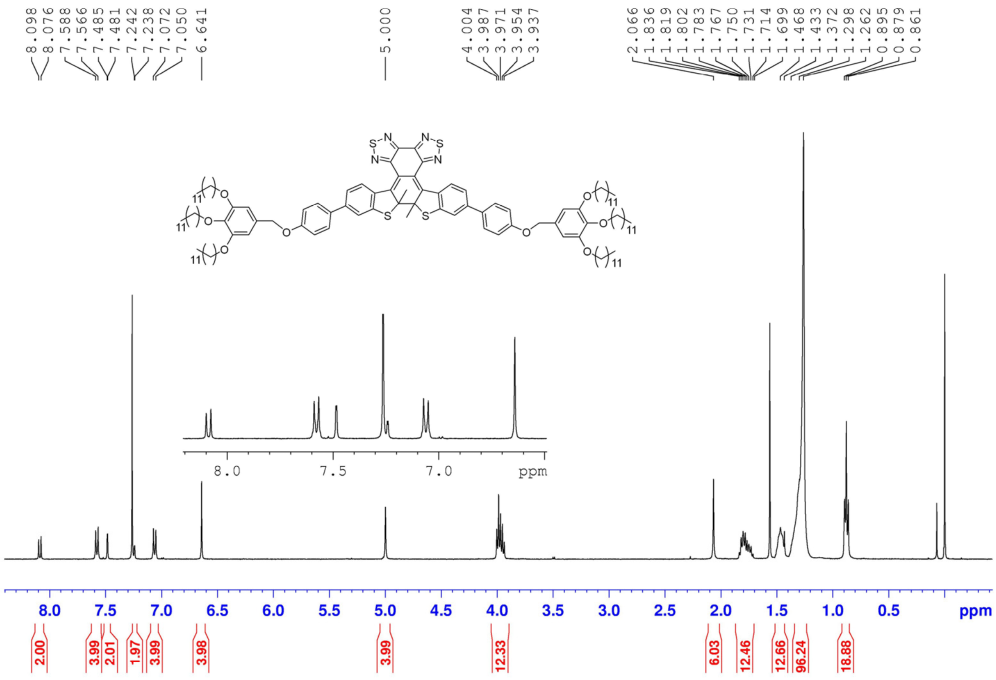

Figure S8. ^{1}H NMR spectrum of compound **1c** in $CDCl_3$.

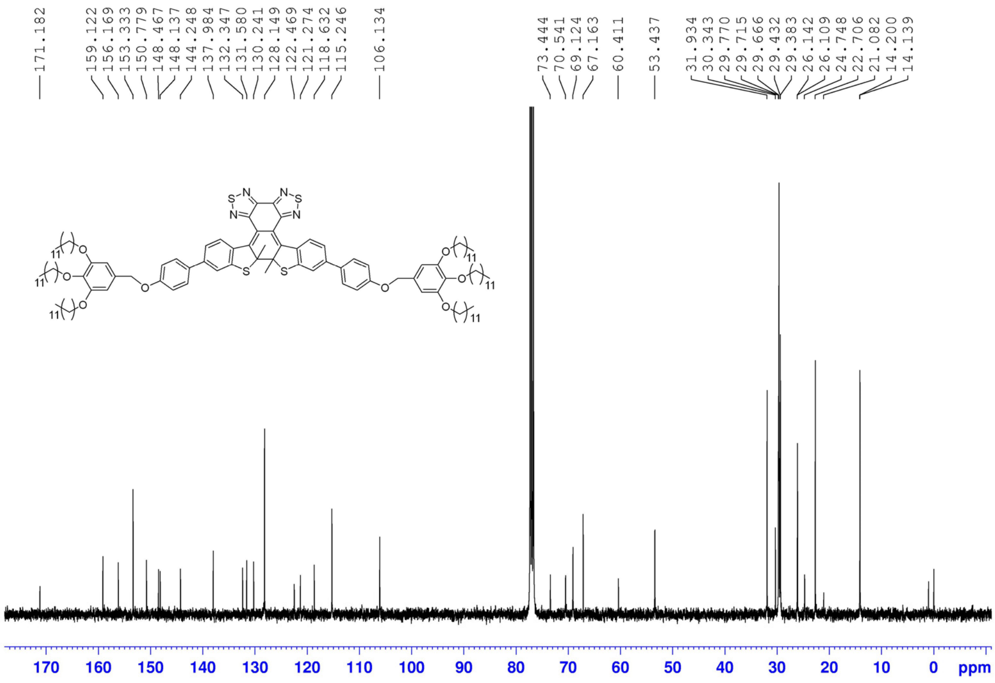

Figure S9. ^{13}C NMR spectrum of **1c** in $CDCl_3$.

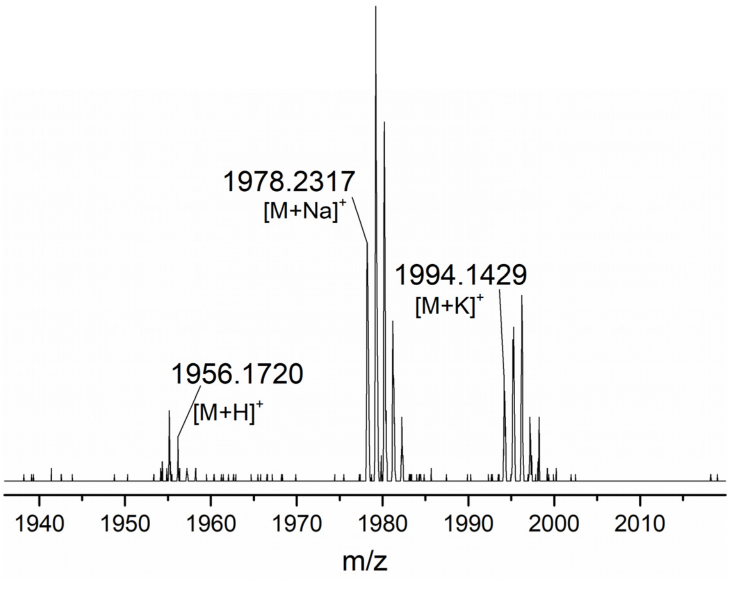

Figure S10. Mass spectrum of **1c**.

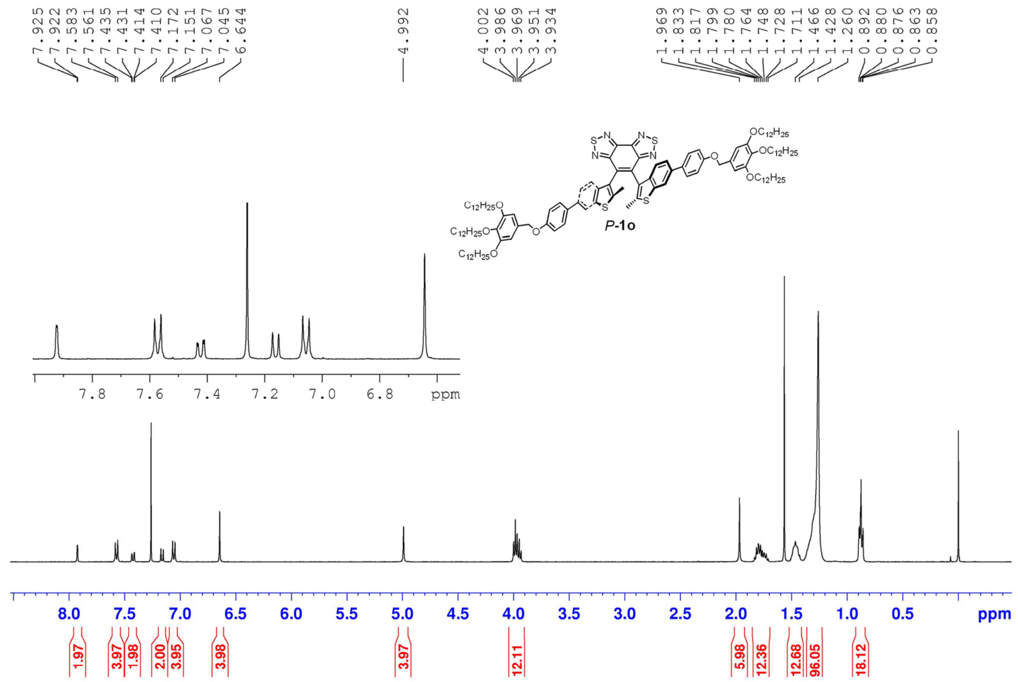

Figure S11. ^{1}H NMR spectrum of compound *P*-**1o** in $CDCl_3$.

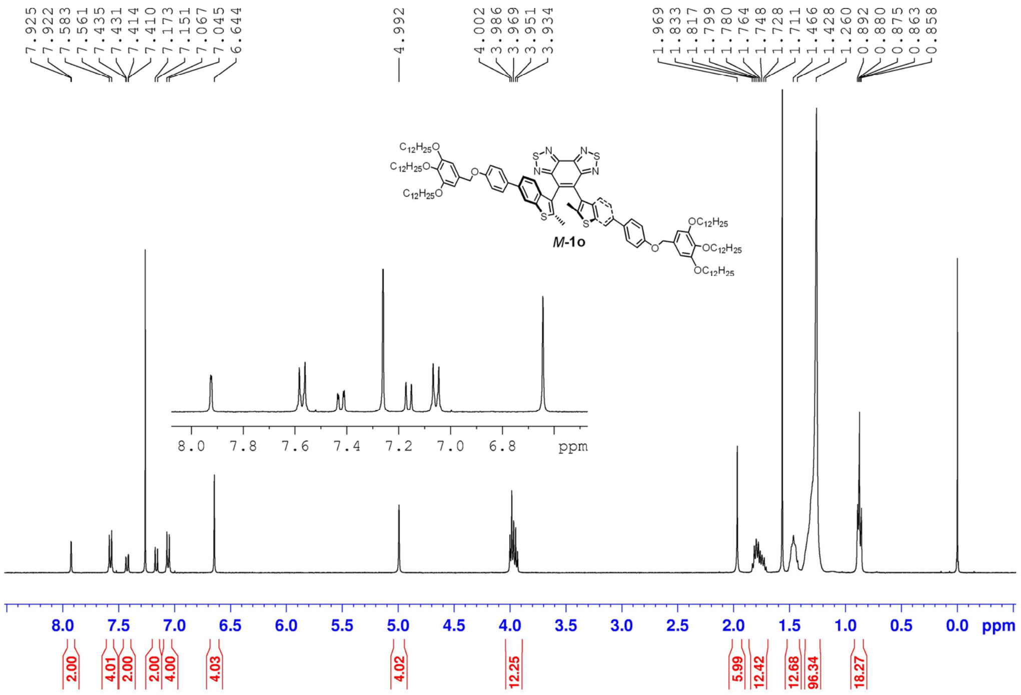

Figure S12. ^{1}H NMR spectrum of compound *M*-**1o** in $CDCl_3$.

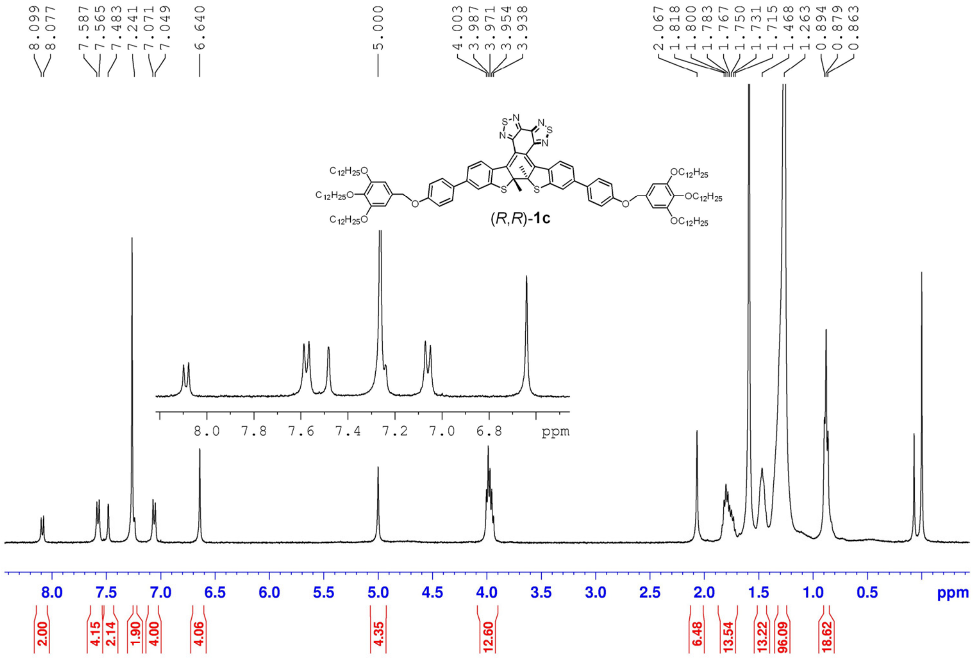

Figure S13. ^{1}H NMR spectrum of compound (*R*,*R*)-**1c** in $CDCl_3$.

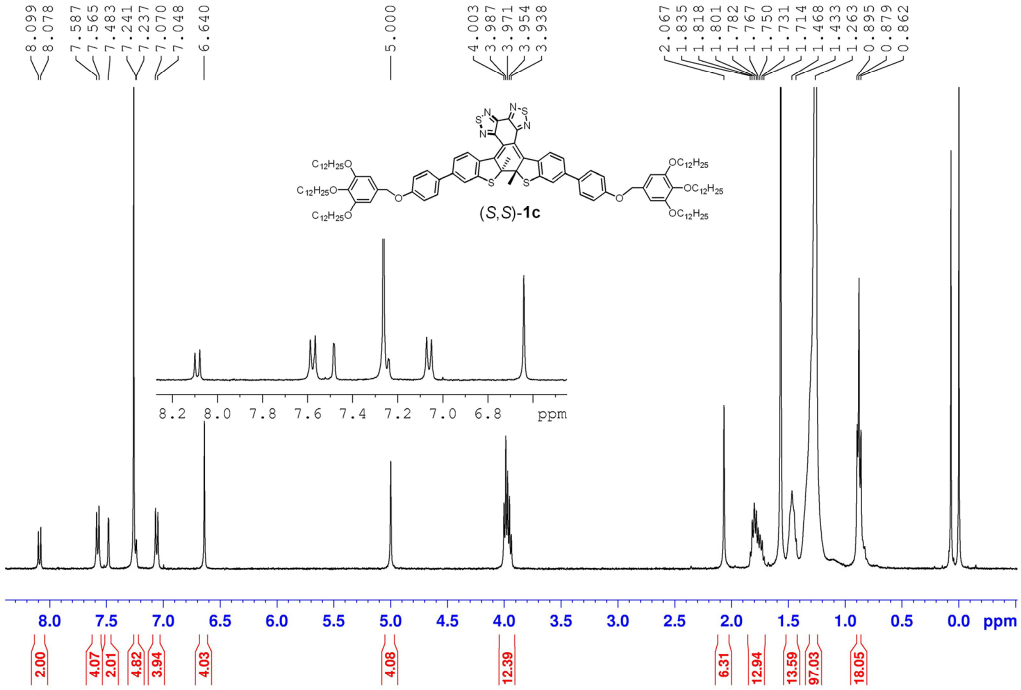

Figure S14. ^{1}H NMR spectrum of compound (*S*,*S*)-**1c** in $CDCl_3$.

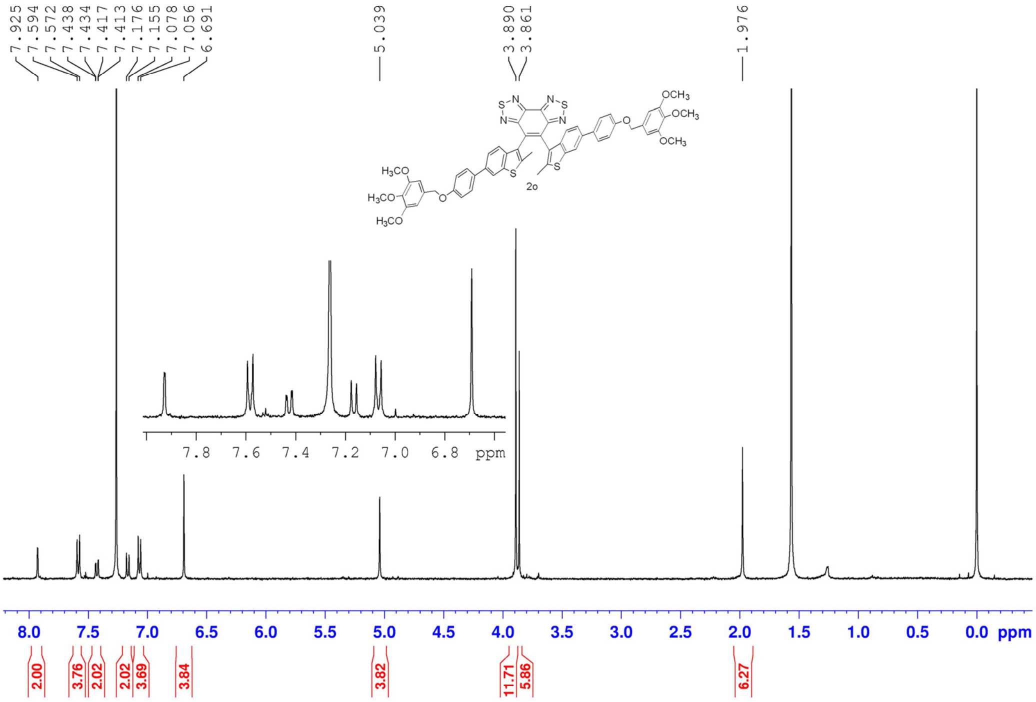

Figure S15. ^{1}H NMR spectrum of compound **2o** in $CDCl_3$.

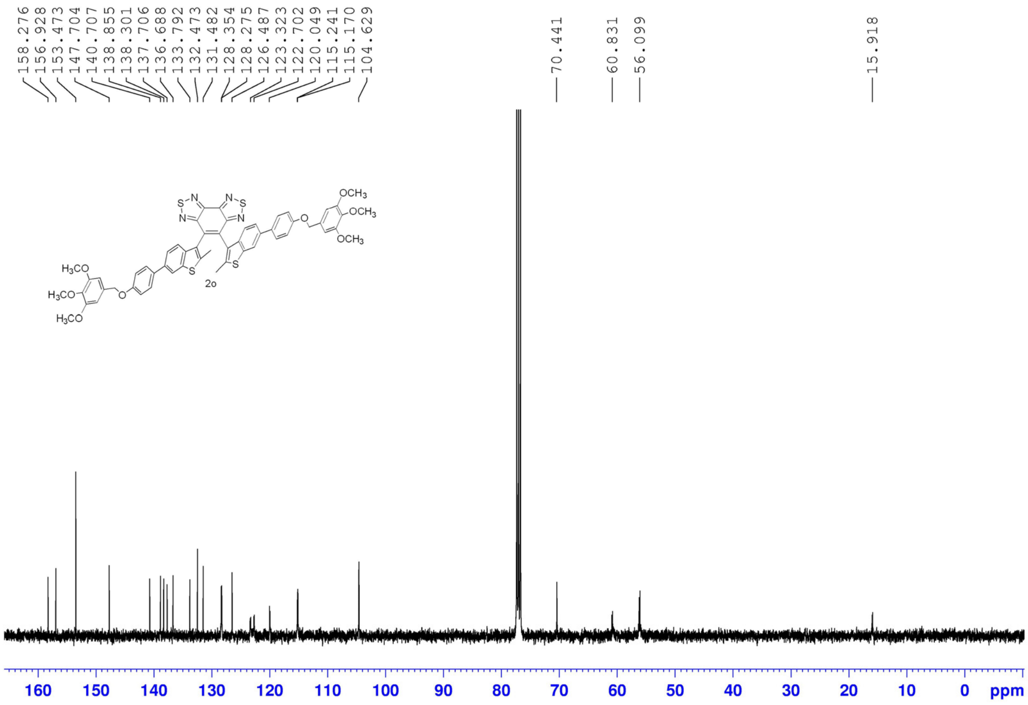

Figure S16. ^{13}C NMR spectrum of **2o** in $CDCl_3$.

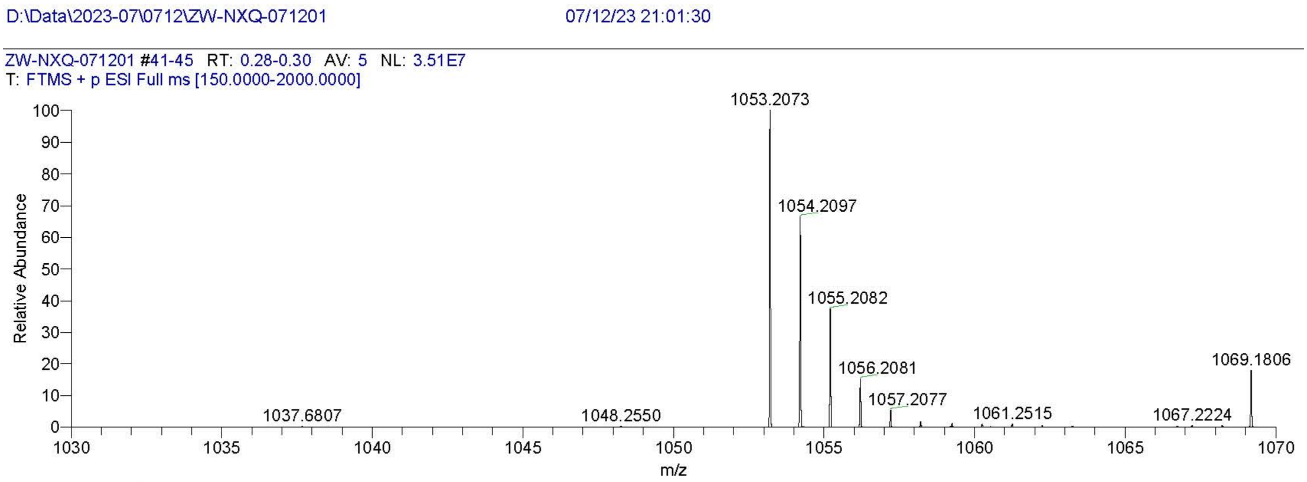

ZW-NXQ-071201#41-45 RT: 0.28-0.30 AV: 5
T: FTMS + p ESI Full ms [150.0000-2000.0000]
m/z= 1052.79-1053.41

m/z	Intensity	Relative	Theo. Mass	Delta (ppm)	Composition
1053.2073	35683820.0	100.00	1053.2091	-1.72	$C_{56}H_{46}O_8N_4NaS_4$

Figure S17. Mass spectrum of **2o**.

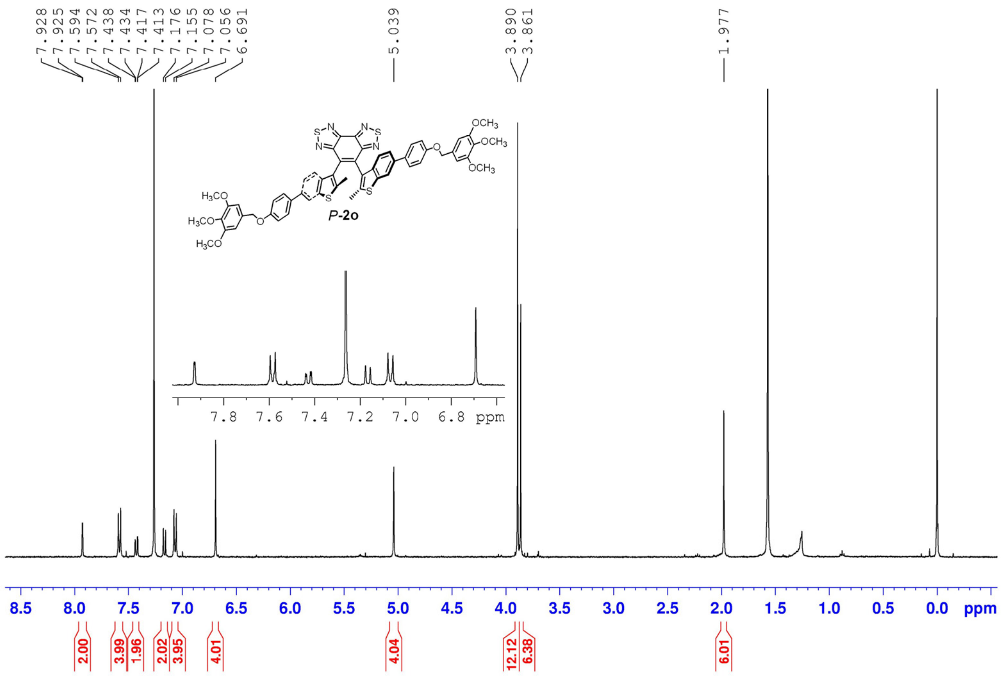

Figure S18 ^{1}H NMR spectrum of compound *P*-**2o** in $CDCl_3$.

D:\Data\2023-07\0712\ZW-NXQ-071201 07/12/23 21:01:30

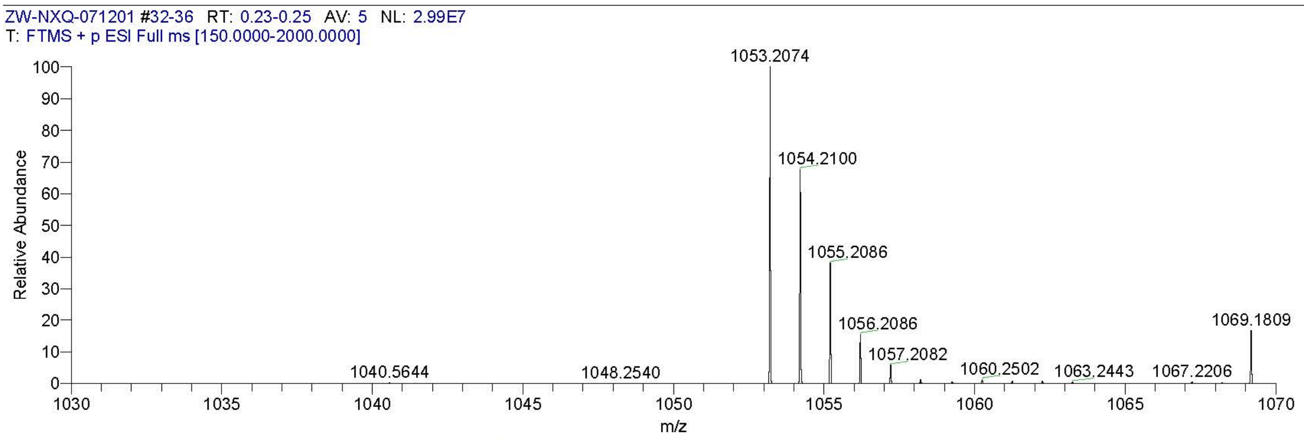

ZW-NXQ-071201#32-36 RT: 0.23-0.25 AV: 5
T: FTMS + p ESI Full ms [150.0000-2000.0000]
m/z= 1053.00-1053.36

m/z	Intensity	Relative	Theo. Mass	Delta (ppm)	Composition
1053.2074	30633998.0	100.00	1053.2091	-1.62	$C_{56}H_{46}O_8N_4NaS_4$

Figure S19. Mass spectrum of *P*-**2o**.

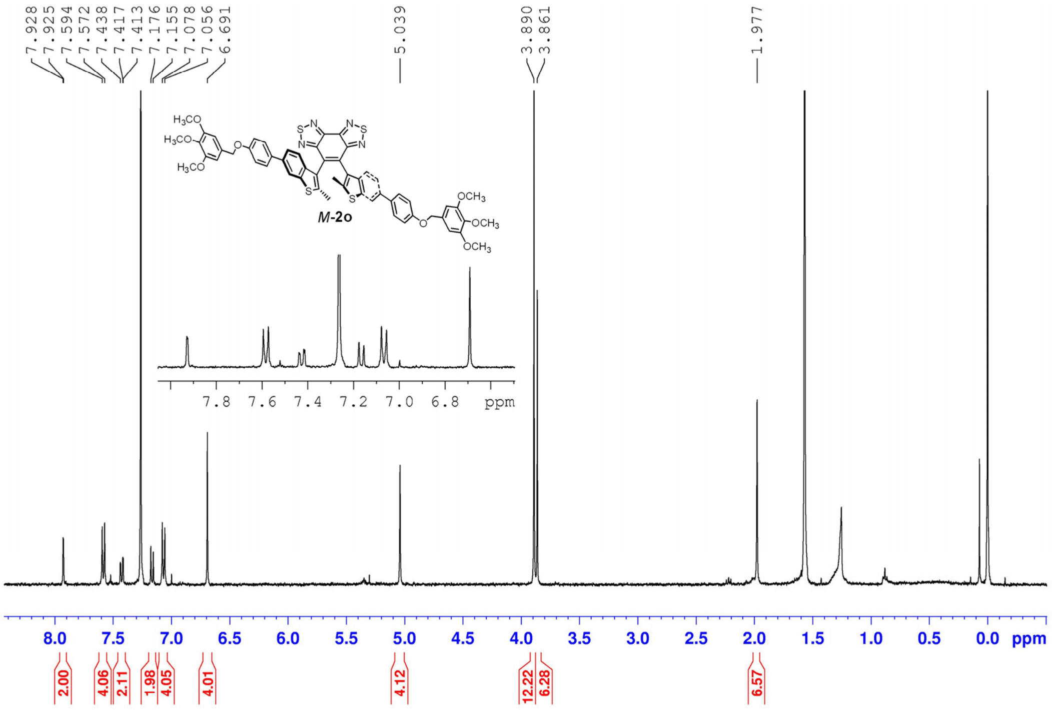

Fig. S20. ^{1}H NMR spectrum of compound *M*-**2o** in $CDCl_3$.

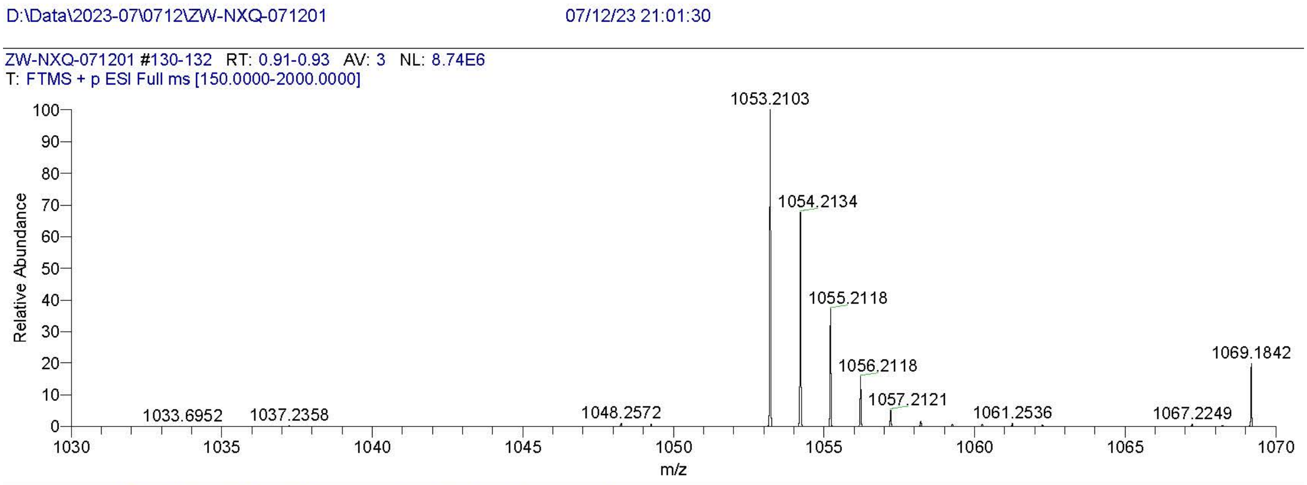

ZW-NXQ-071201#130-132 RT: 0.91-0.93 AV: 3
T: FTMS + p ESI Full ms [150.0000-2000.0000]
m/z= 1053.07-1053.41

m/z	Intensity	Relative	Theo. Mass	Delta (ppm)	Composition
1053.2103	8914533.0	100.00	1053.2091	1.23	$C_{56}H_{46}O_8N_4NaS_4$

Figure S21. Mass spectrum of *M*-**2o**.

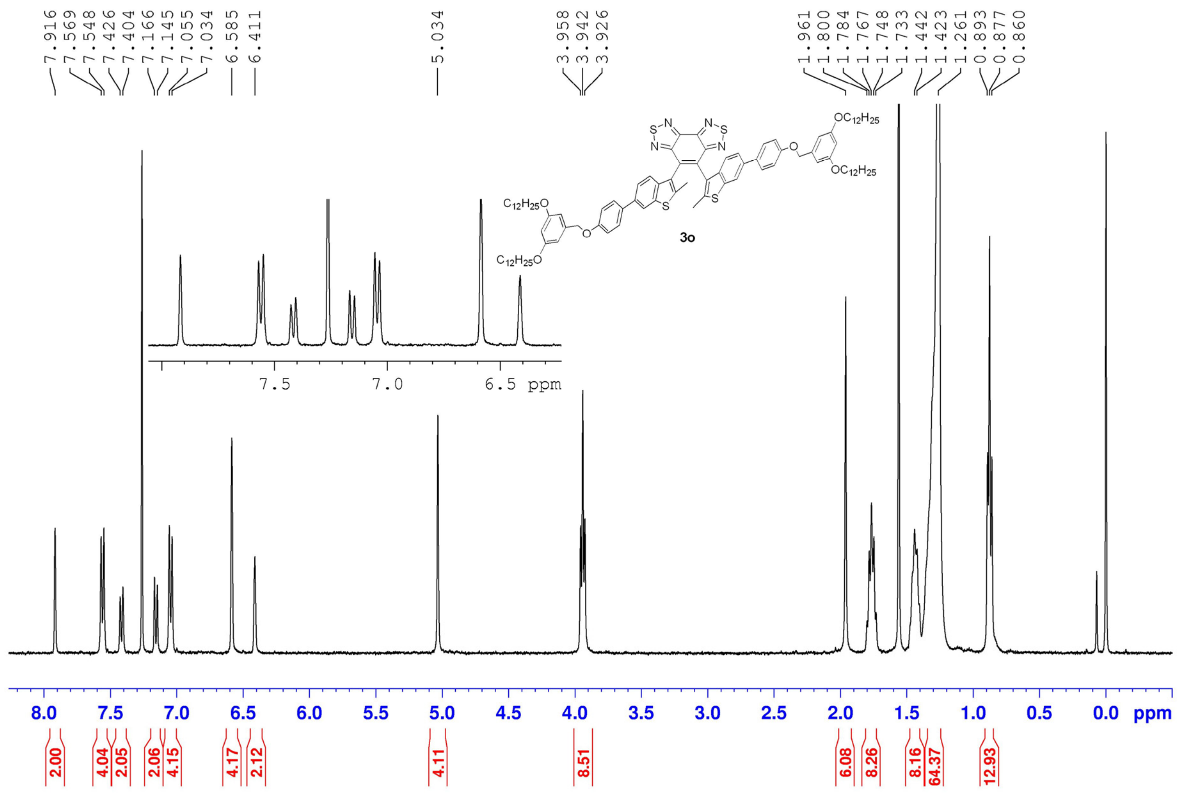

Figure S22. ^{1}H NMR spectrum of compound **3o** in $CDCl_3$.

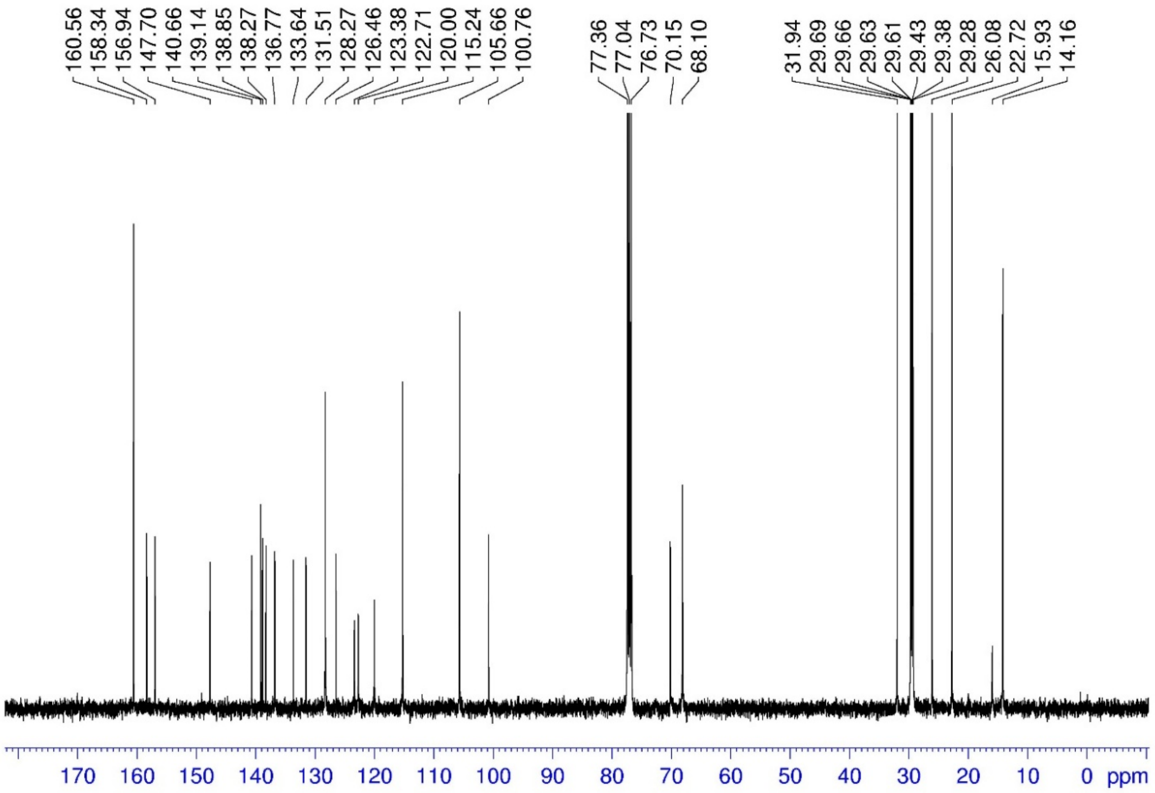

Figure S23. ^{13}C NMR spectrum of compound **3o** in $CDCl_3$.

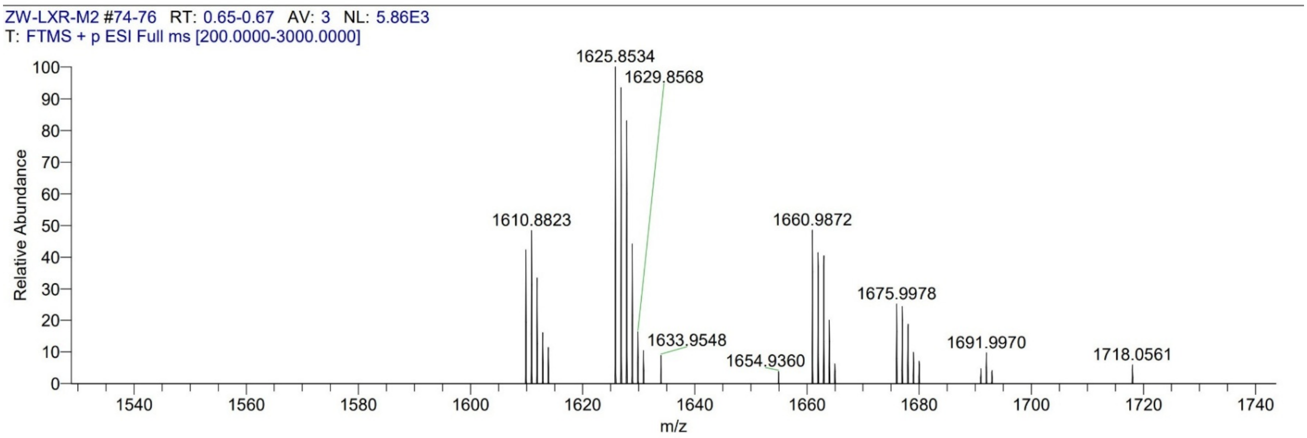

ZW-LXR-M2#74-78 RT: 0.65-0.68 AV: 5
T: FTMS + p ESI Full ms [200.0000-3000.0000]
m/z= 1624.82-1626.76

m/z	Intensity	Relative	Theo. Mass	Delta (ppm)	Composition
1625.8534	3530.8	100.00	1625.8505	2.88	$C_{98}H_{130}O_6N_4KS_4$

Figure S24. Mass spectrum of **3o**.

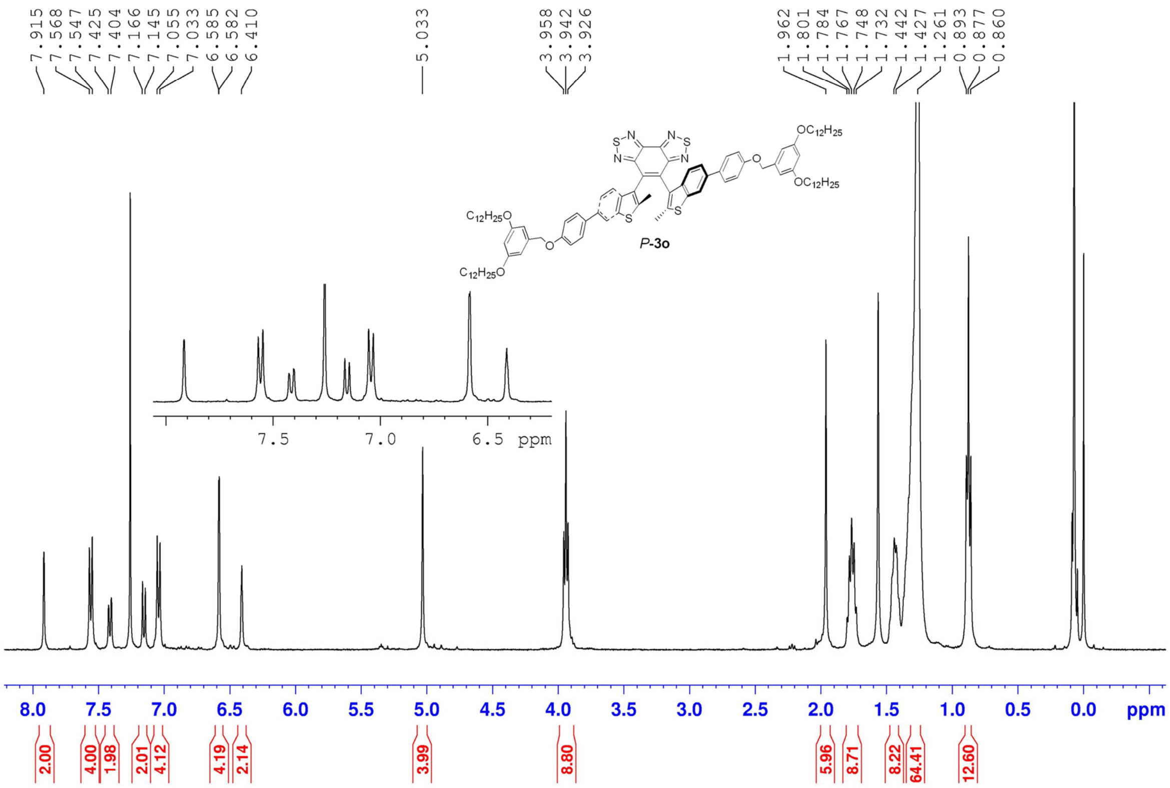

Figure S25. ^{1}H NMR spectrum of compound *P*-**3o** in $CDCl_3$.

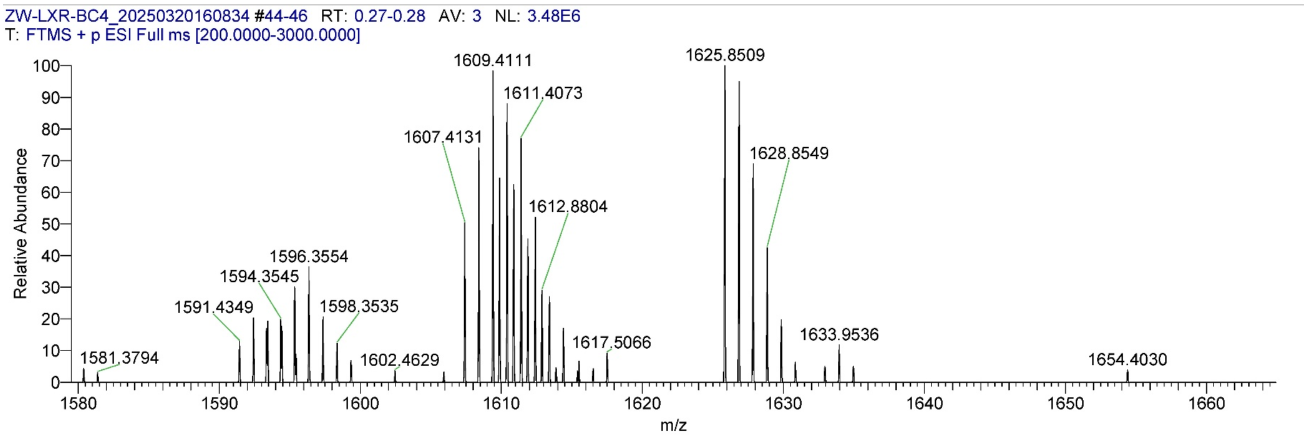

ZW-LXR-BC4_20250320160834#44-46 RT: 0.27-0.28 AV: 3
T: FTMS + p ESI Full ms [200.0000-3000.0000]
m/z= 1625.30-1626.24

m/z	Intensity	Relative	Theo. Mass	Delta (ppm)	Composition
1625.8509	3485516.0	100.00	1625.8505	0.44	$C_{98}H_{130}O_6N_4KS_4$

Figure S26. Mass spectrum of *P*-**3o**.

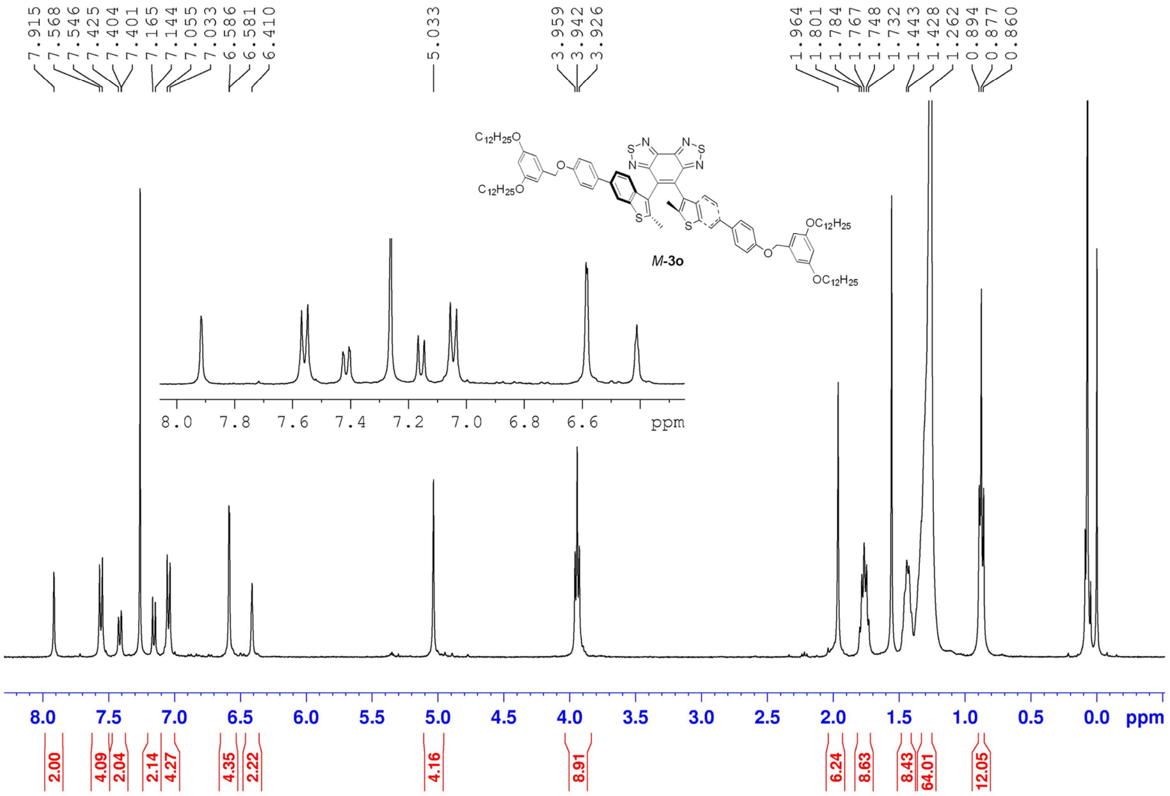

Figure S27. ^{1}H NMR spectrum of compound *M*-**3o** in $CDCl_3$.

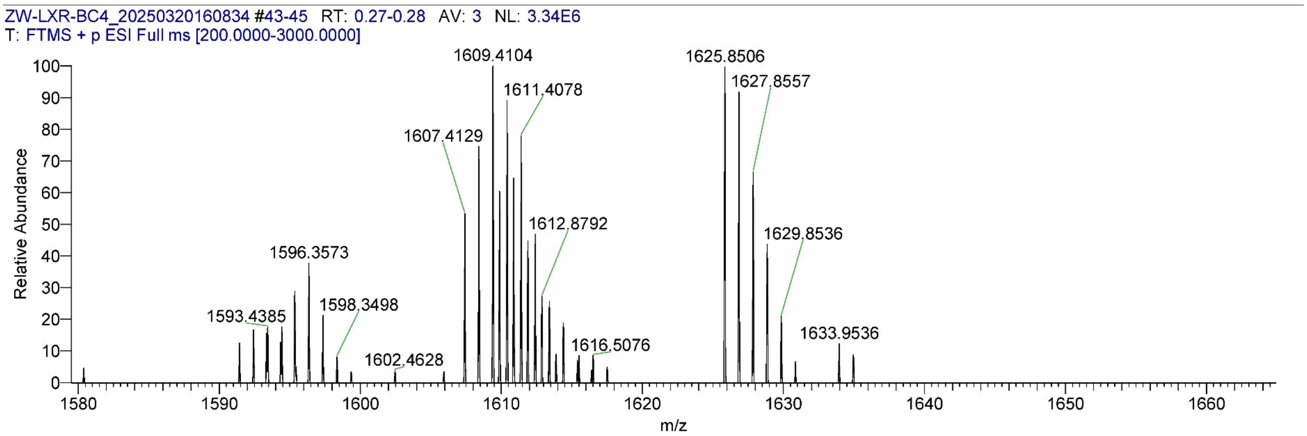

ZW-LXR-BC4_20250320160834#43-45 RT: 0.27-0.28 AV: 3
T: FTMS + p ESI Full ms [200.0000-3000.0000]
m/z= 1625.30-1626.24

m/z	Intensity	Relative	Theo. Mass	Delta (ppm)	Composition
1625.8506	3328163.5	100.00	1625.8505	0.10	$C_{98}H_{130}O_6N_4KS_4$

Figure S28. Mass spectrum of *M*-**3o**.

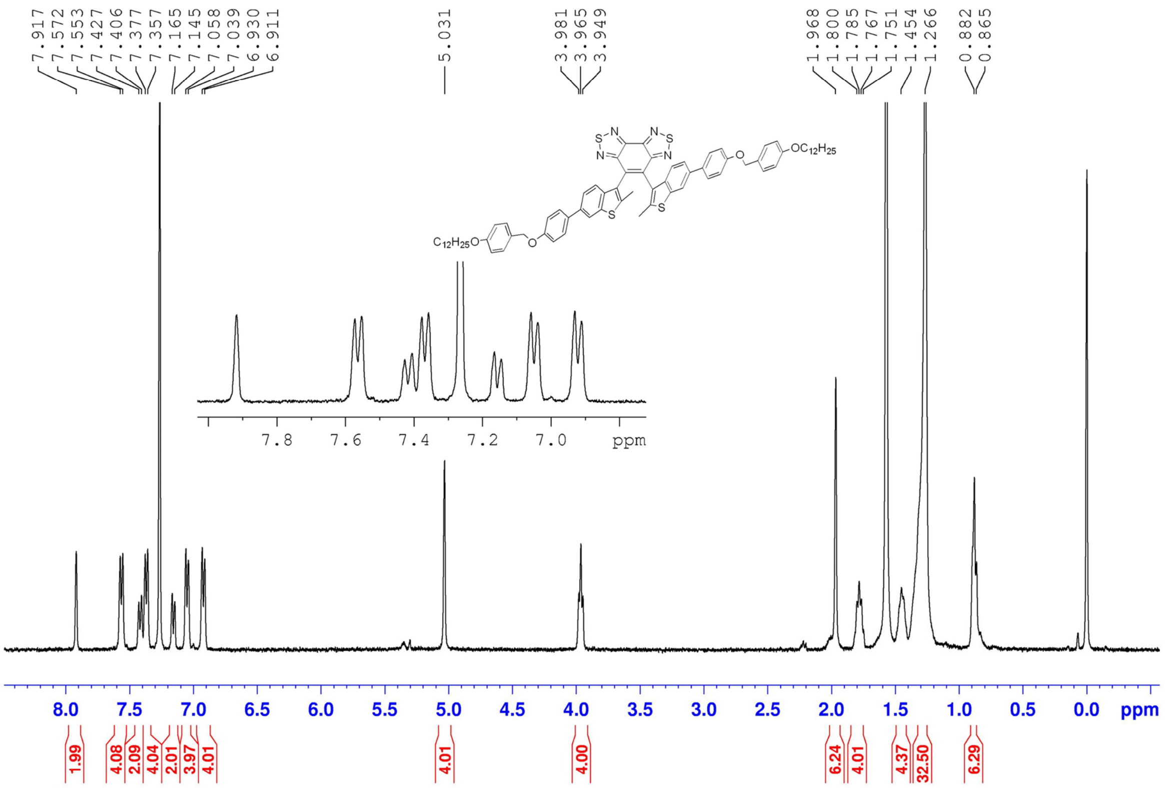

Figure S29. ^{1}H NMR spectrum of compound **4o** in $CDCl_3$

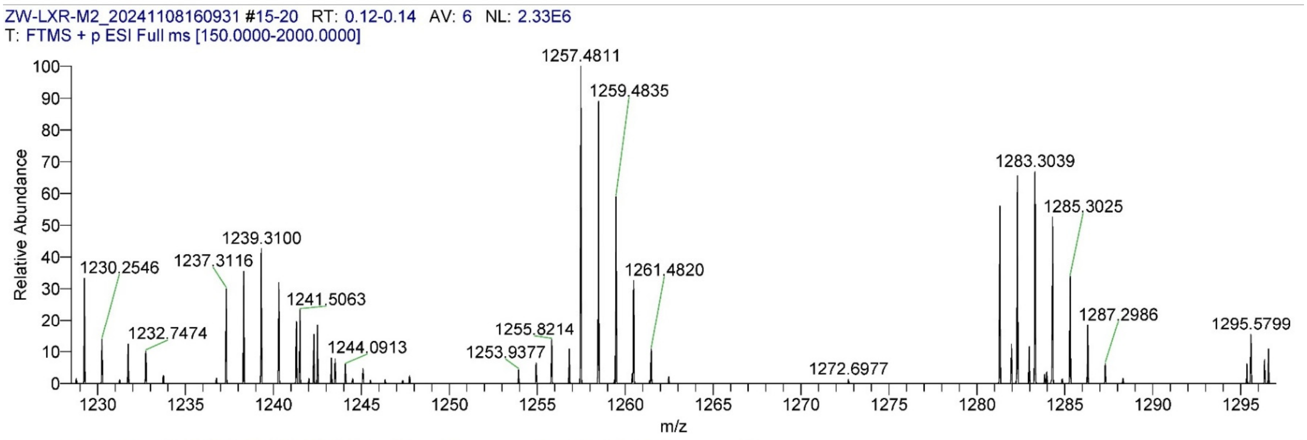

ZW-LXR-M2_20241108160931#15-20 RT: 0.12-0.14 AV: 6
T: FTMS + p ESI Full ms [150.0000-2000.0000]
m/z= 1257.22-1257.71

m/z	Intensity	Relative	Theo. Mass	Delta (ppm)	Composition
1257.4811	2358962.0	100.00	1257.4850	-3.93	$C_{74}H_{82}O_4N_4KS_4$

Figure S30. Mass spectrum of **4o**.

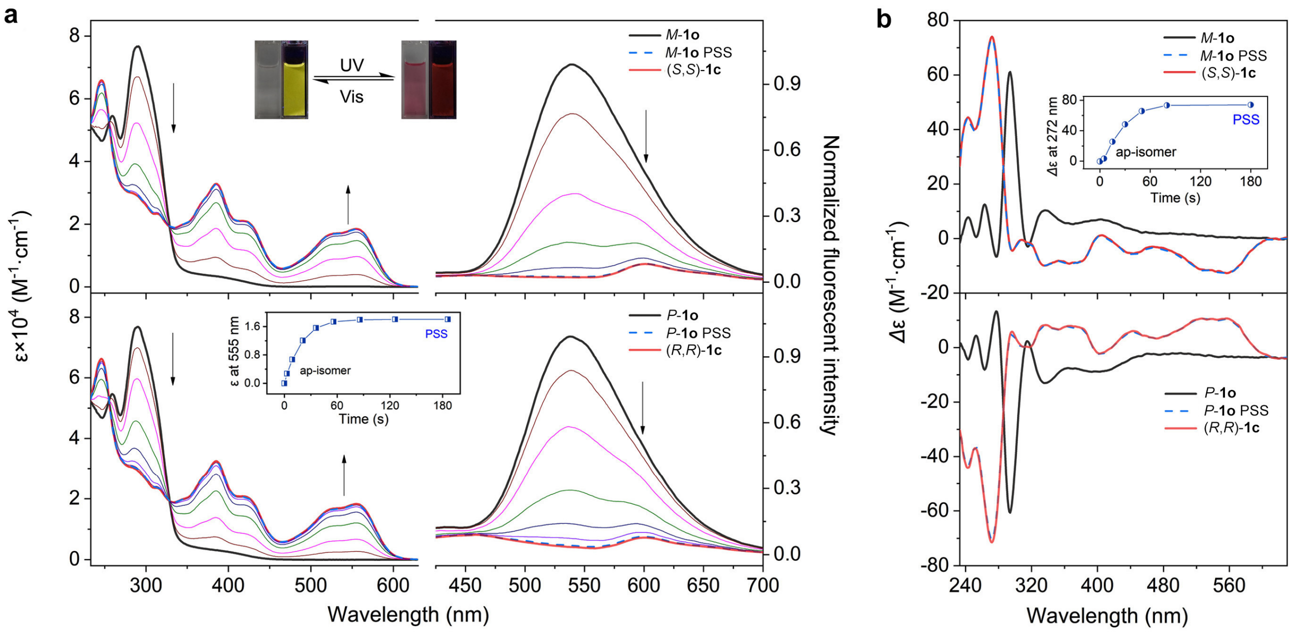

Figure S31. Photoresponsive properties and AIE effect of the intrinsic chiral photoswitch with long alkyl chains. **(a)** Absorption and fluorescent spectra changes of *P*-**1o** and *M*-**1o** upon irradiation with UV light. The inset images show the color and fluorescence changes between open form triggered by UV (λ = 313 ± 10 nm) and visible light (λ > 510 nm), respectively. **(b)** Circular dichroism spectra changes of *P*-**1o** and *M*-**1o** upon irradiation with UV light and corresponding closed form.

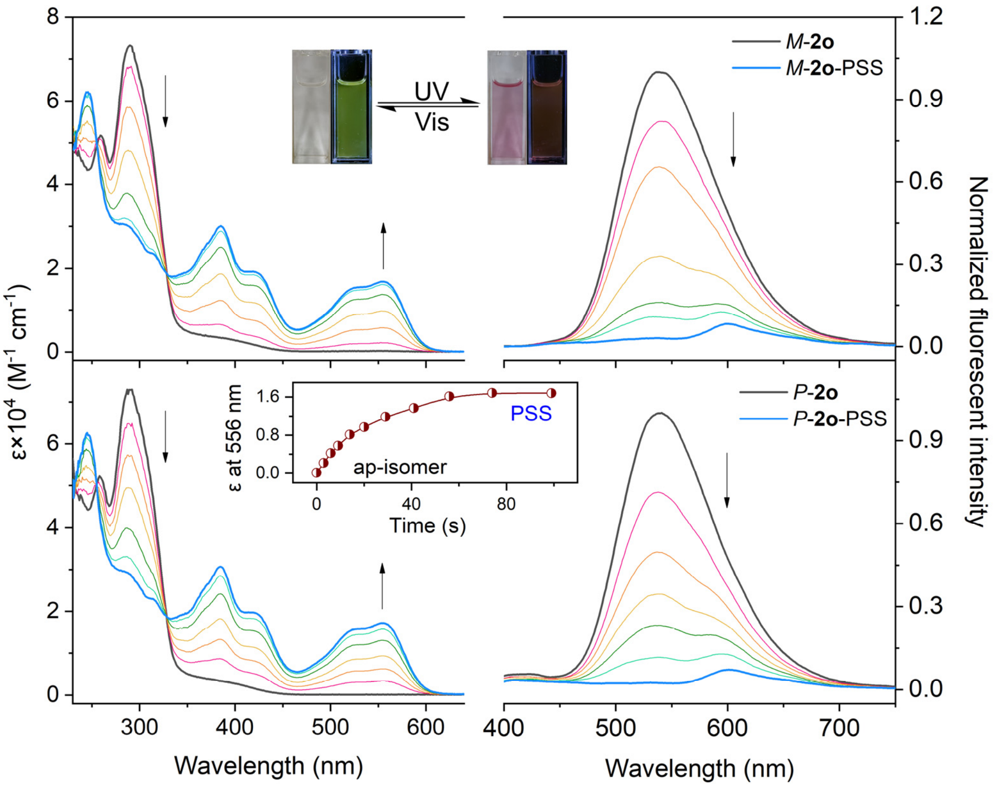

Figure S32. Absorption and fluorescent spectra changes of *P*-**2o** and *M*-**2o** upon irradiation with UV light. The inset images show the color and fluorescence changes between open form triggered by UV (λ = 313 ± 10 nm) and visible light (λ > 510 nm), respectively.

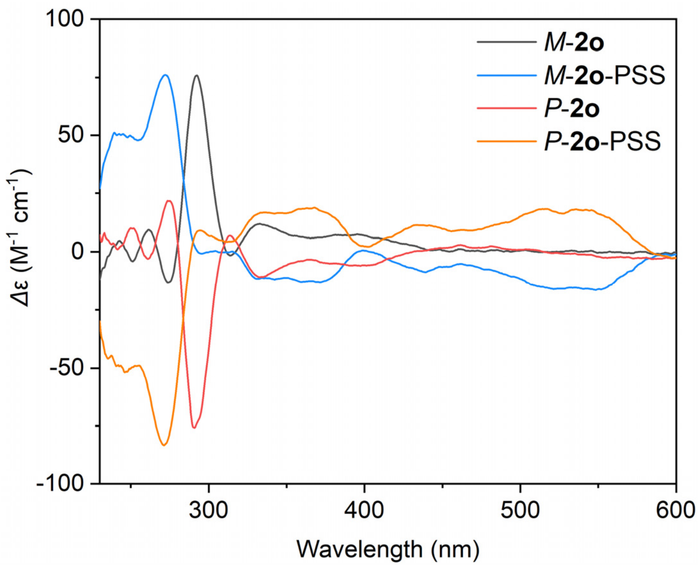

Figure S33. Circular dichroism spectra changes of **2o** upon irradiation with UV light (λ = 313 ± 10 nm).

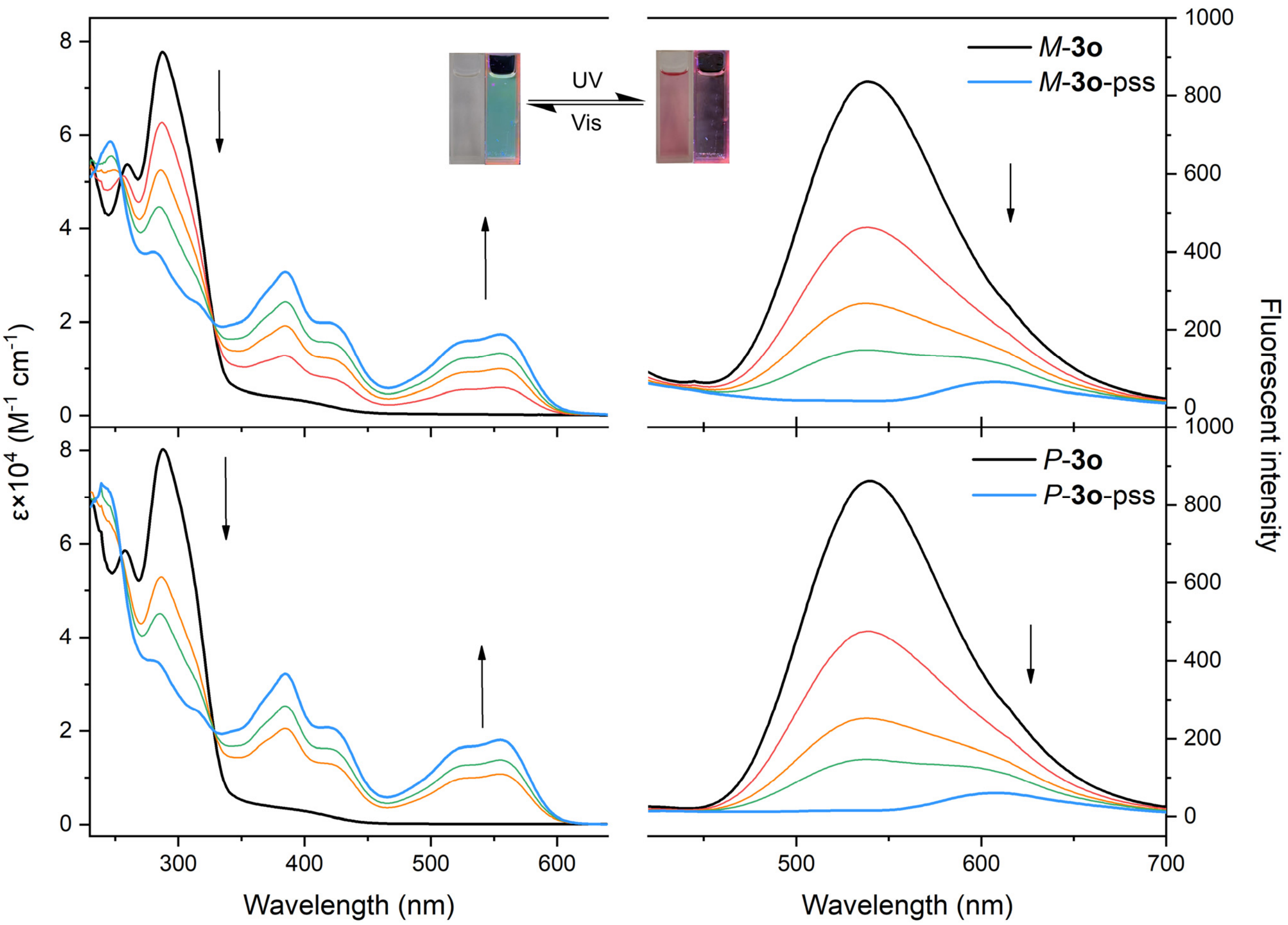

Figure S34. Absorption and fluorescent spectra changes of *P*-**3o** and *M*-**3o** upon irradiation with UV light. The inset images show the color and fluorescence changes between open form triggered by UV (λ = 313 ± 10 nm) and visible light (λ > 510 nm), respectively.

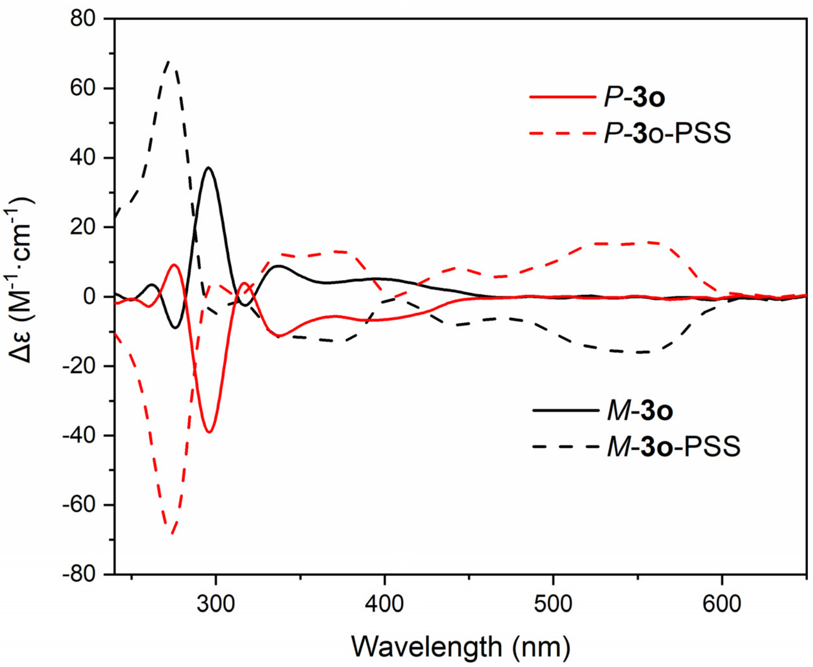

Figure S35. Circular dichroism spectra changes of **3o** upon irradiation with UV light (λ = 313 ± 10 nm).

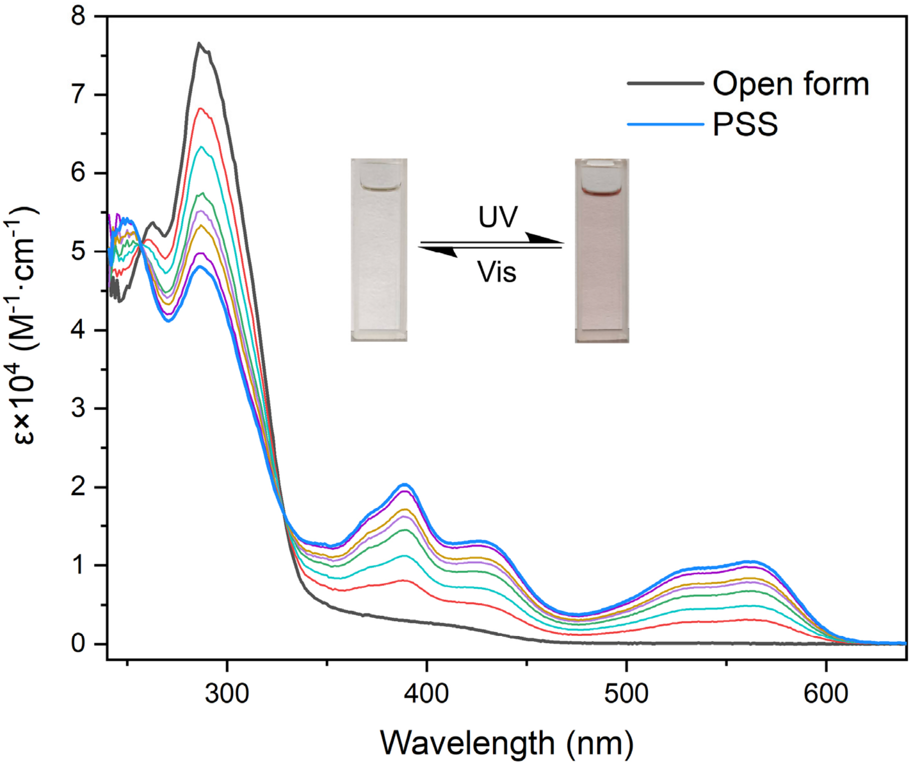

Figure S36. Absorption spectra changes of **4o** upon irradiation with UV light (λ = 313 ± 10 nm). The inset images show the color change between open form triggered by UV (λ = 313 ± 10 nm) and visible light (λ > 510 nm), respectively.

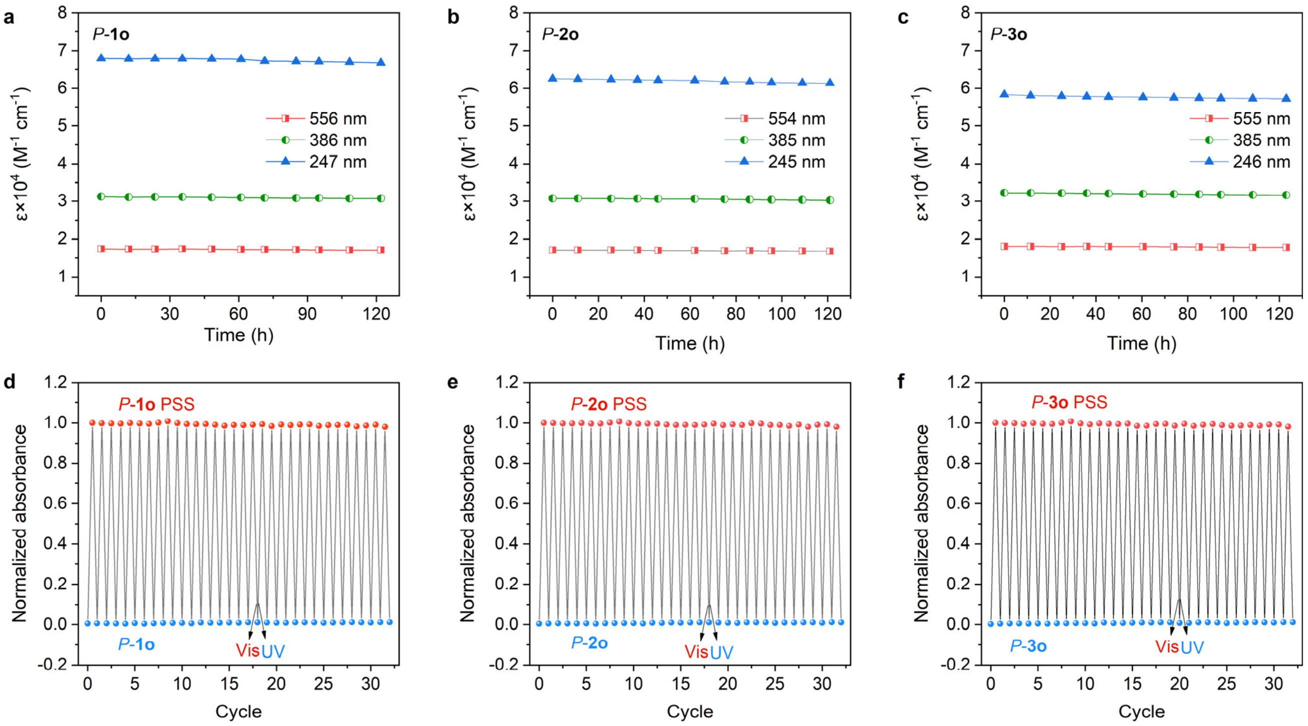

Figure S37. **(a-c)** Thermal stability of **(a)** *P*-**1o**; **(b)** *P*-**2o**; **(c)** *P*-**3o** at PSS in THF solution of 2.0×10^{-5} mol L^{-1}. **(d-f)**, Fatigue resistance of **(d)** *P*-**1o**; **(e)** *P*-**2o**; **(f)** *P*-**3o** irradiated by UV (λ = 313 ± 10 nm) and visible light (λ > 510 nm), respectively.

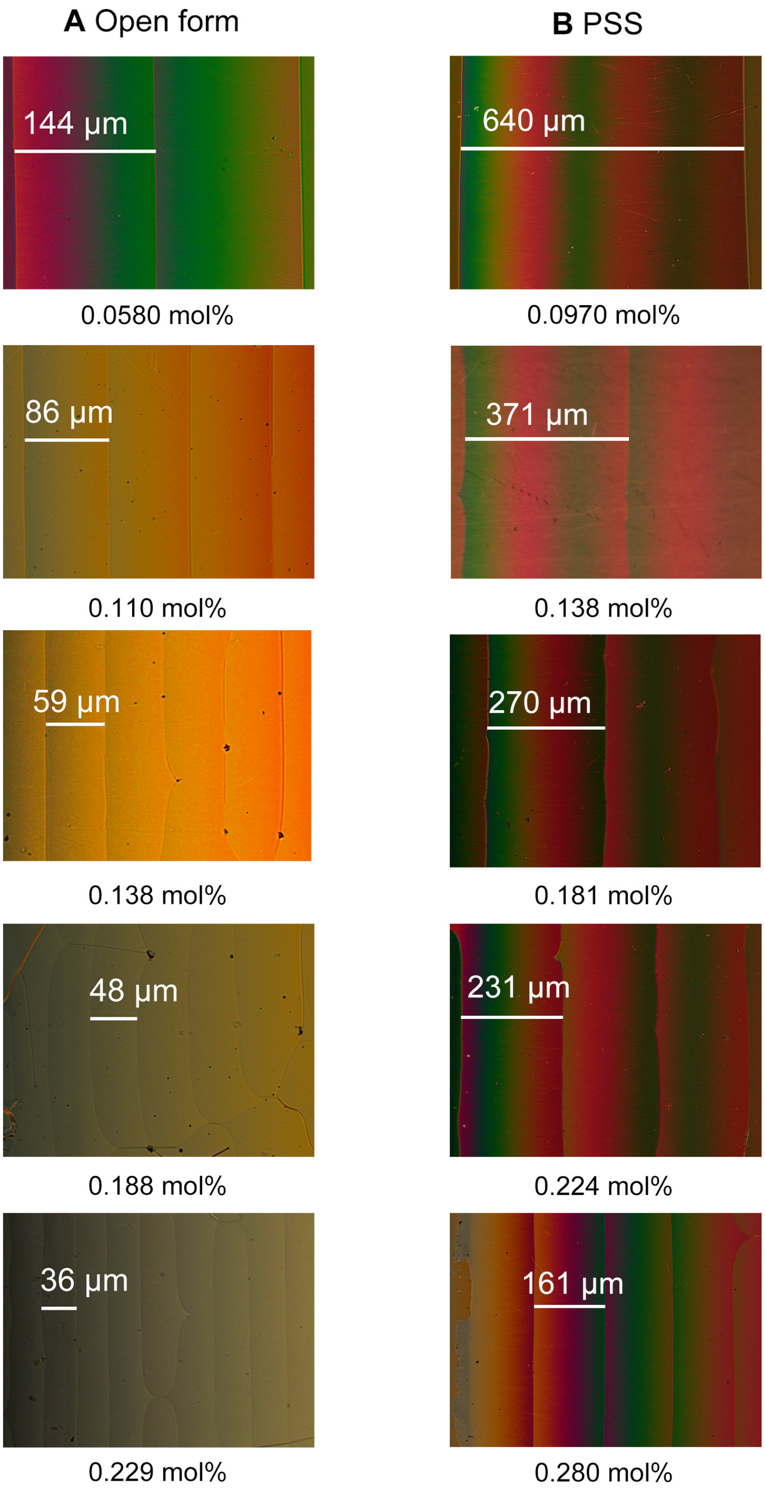

Figure S38. Stripe-wedge Grandjean−Cano cell containing different concentration of **(a)** *P*-**1o** and **(b)** corresponding PSS after UV light irradiation at 365 ± 20 nm.

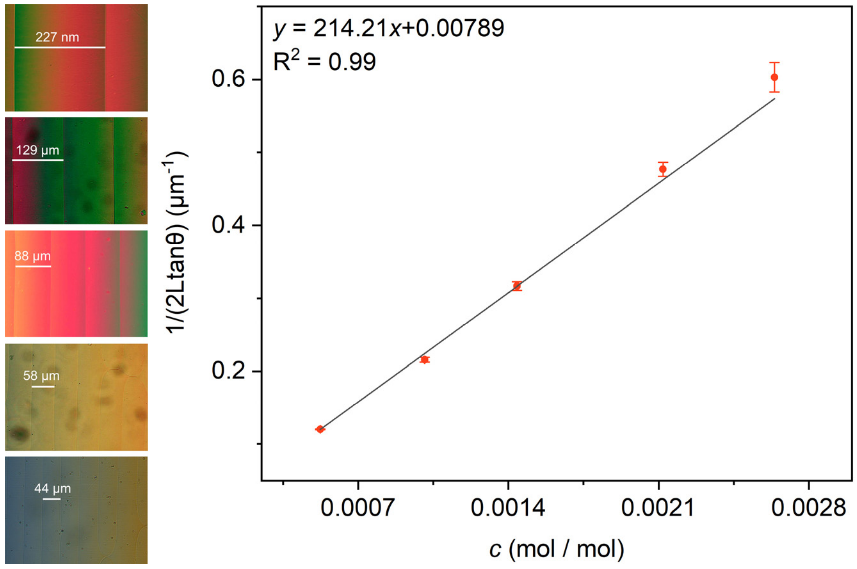

Figure S39. HTP of *P*-**2o** in TEB300

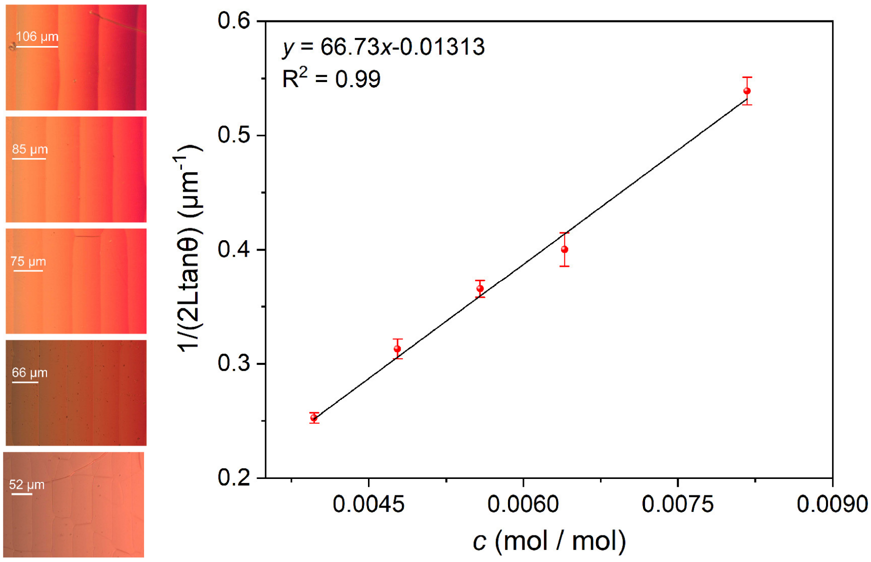

Figure S40. HTP of *P*-**2o** at PSS (irradiated by 365-nm light) in TEB300

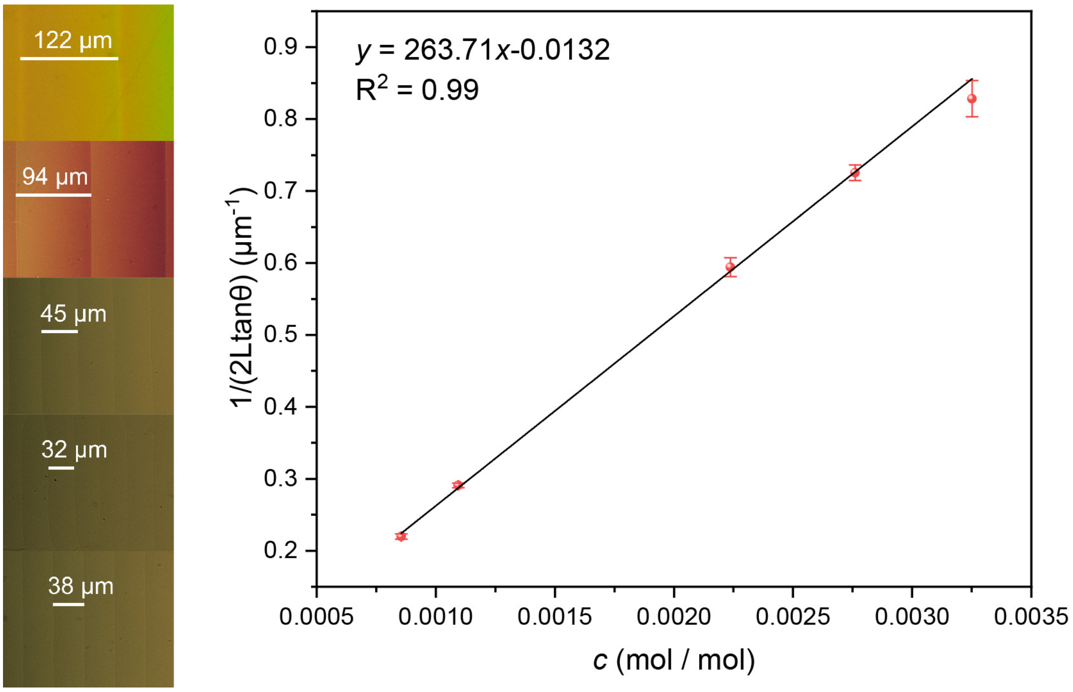

Figure S41. HTP of *P*-**3o** in TEB300

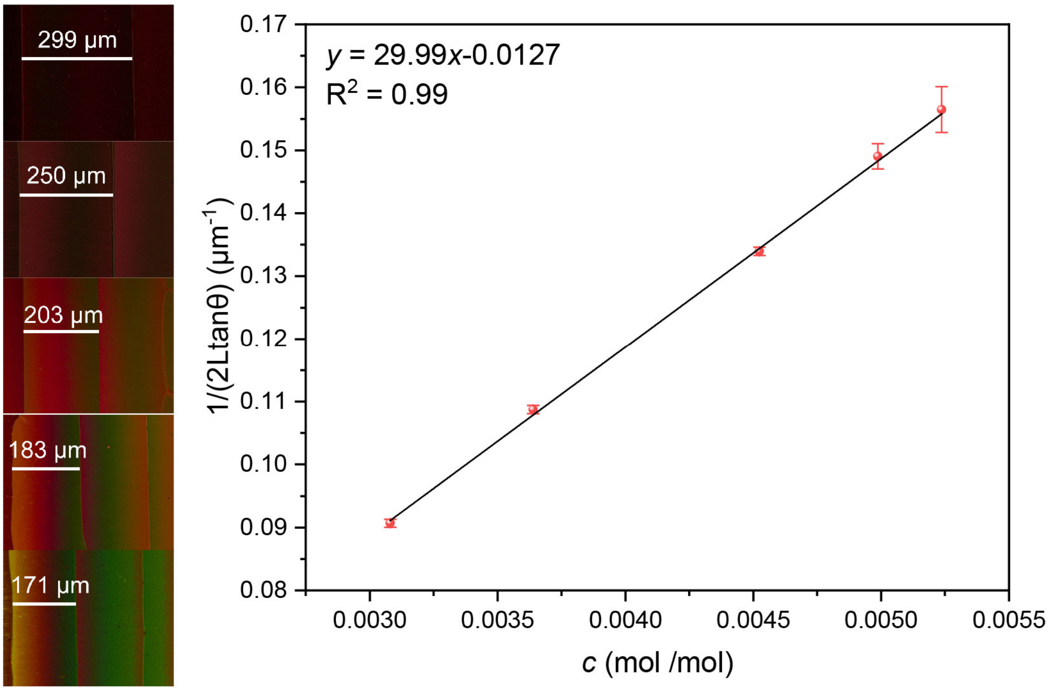

Figure S42. HTP of *P*-**3o** at PSS (irradiated by 365-nm light) in TEB300

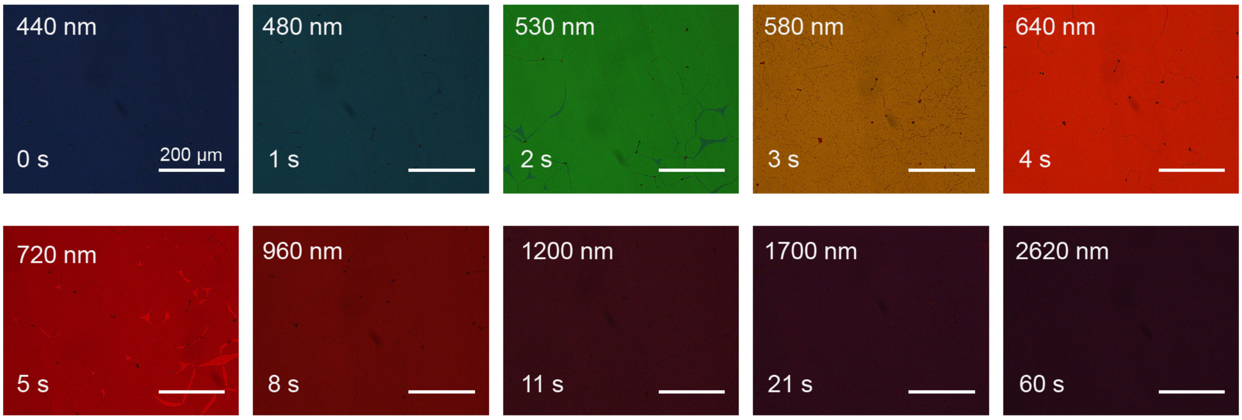

Figure S43. Reflection color change of photoresponsive helical LC containing 1.98 mol% *P*-**1o** in commercially available LC (E7) in a 4-μm thick planar cell upon exposure to UV light (365 ± 20 nm) with different time.

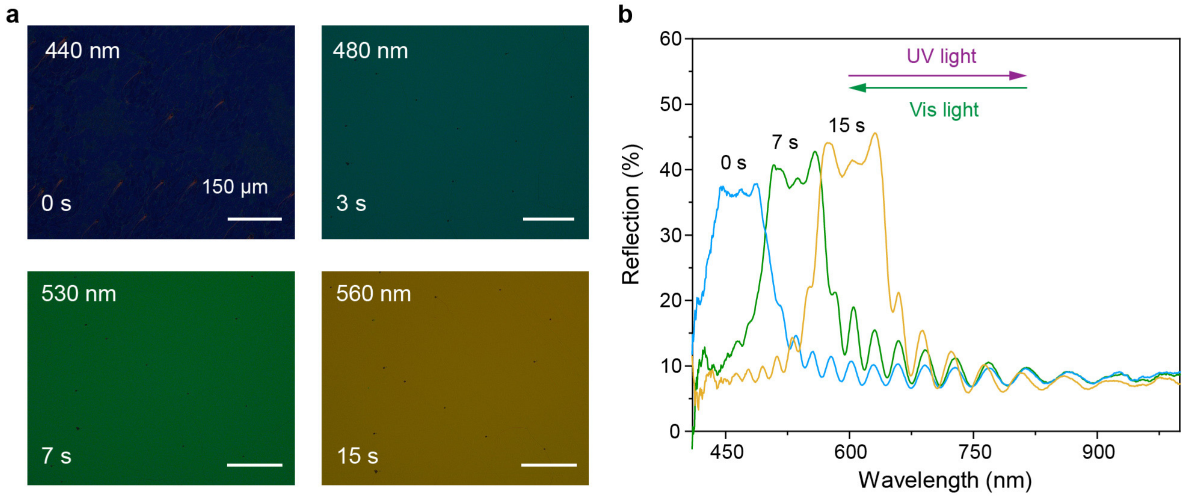

Figure S44. (a) Reflection color change of photoresponsive helical LC containing 0.47 mol% *P*-**2o** and 1.45 mol% R5011 in commercially available LC (E7) in a 4-μm thick planar cell upon exposure to UV light (365 ± 20 nm) with different time. **(b)** Reflection wavelength of LC film containing 0.47 mol% *P*-**2o** and 1.45 mol% R5011 in commercially available LCs in a 4-μm thick planar cell upon exposure to UV light (365 ± 20 nm) with different irradiation time. Due to poor solubility of *P*-**2o** in liquid crystal, only 0.47 mol% *P*-**2o** can be doped into liquid crystal with well texture and photoresponsive performance.

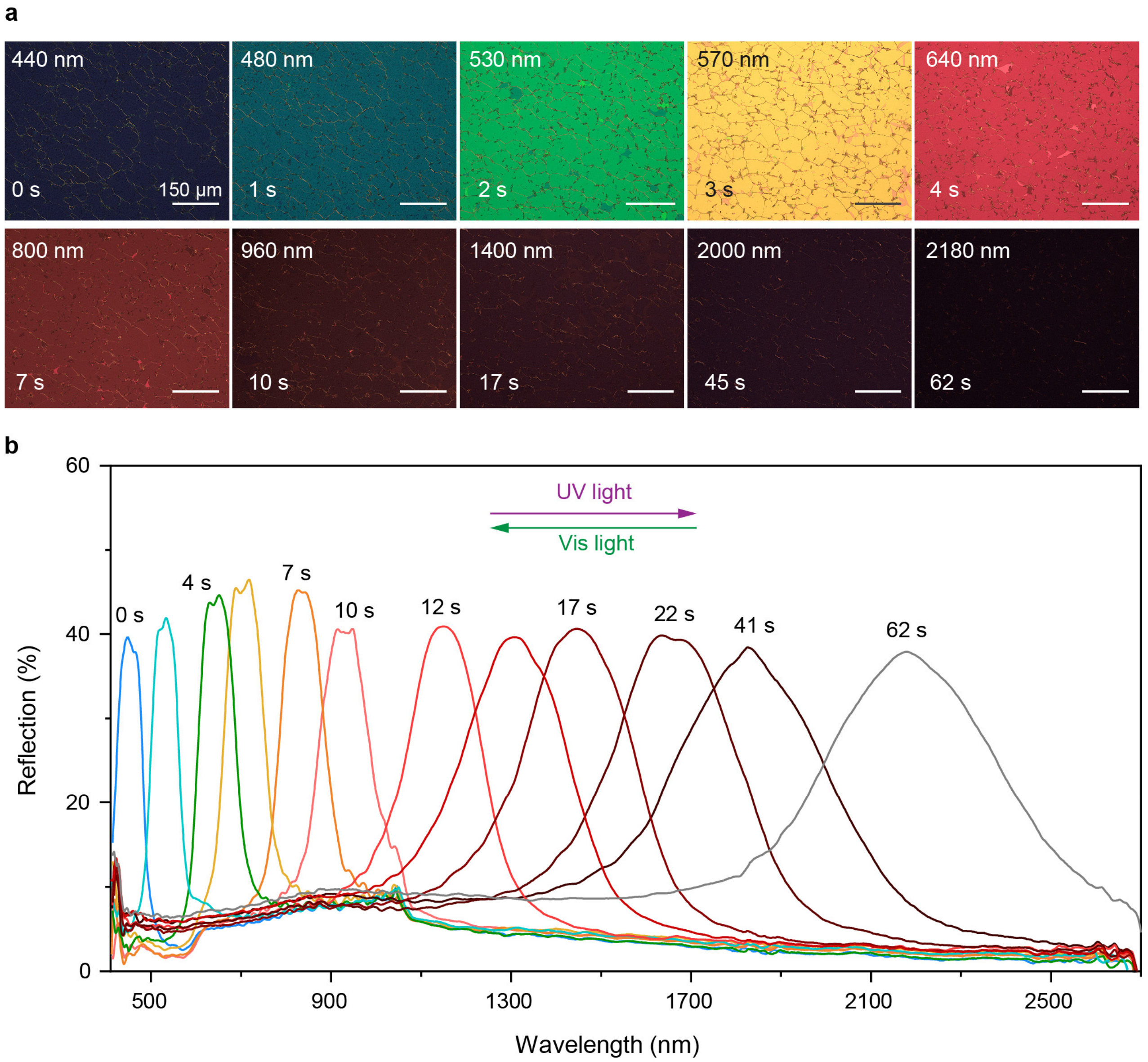

Figure S45 (a) Reflection color change of photoresponsive helical LC containing 2.27 mol% *P*-**3o** in commercially available LC (E7) in a 4-μm thick planar cell upon exposure to UV light (365 ± 20 nm) with different time. **(b)** Reflection wavelength of LC film containing 2.27 mol% *P*-**3o** in commercially available LCs in a 4-μm thick planar cell upon exposure to UV light (365 ± 20 nm) with different irradiation time.

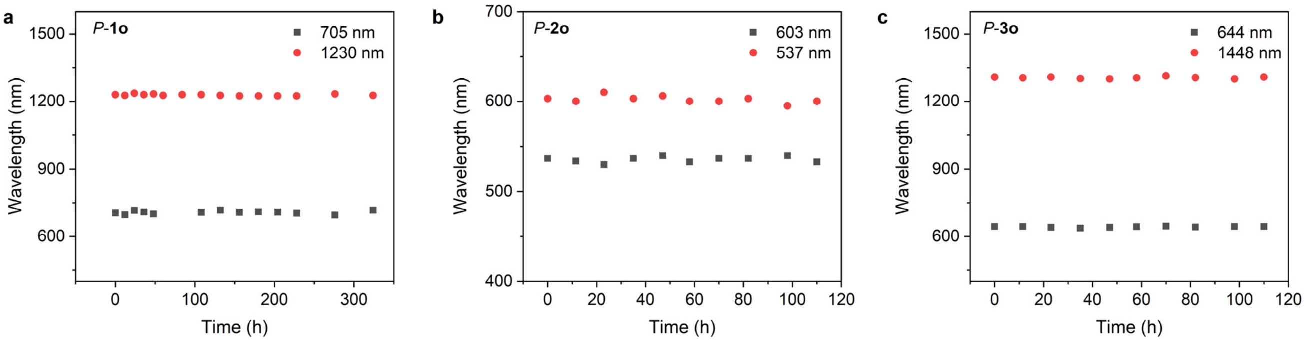

Figure S46. Thermal stability of photoresponsive LC with **(a)** *P*-**1o** (1.98 mol%); **(b)** *P*-**2o** (0.47 mol%) and R5011 (1.45 mol%); **(c)** *P*-**3o** (2.27 mol%) in E7, which shows excellent thermal stability.

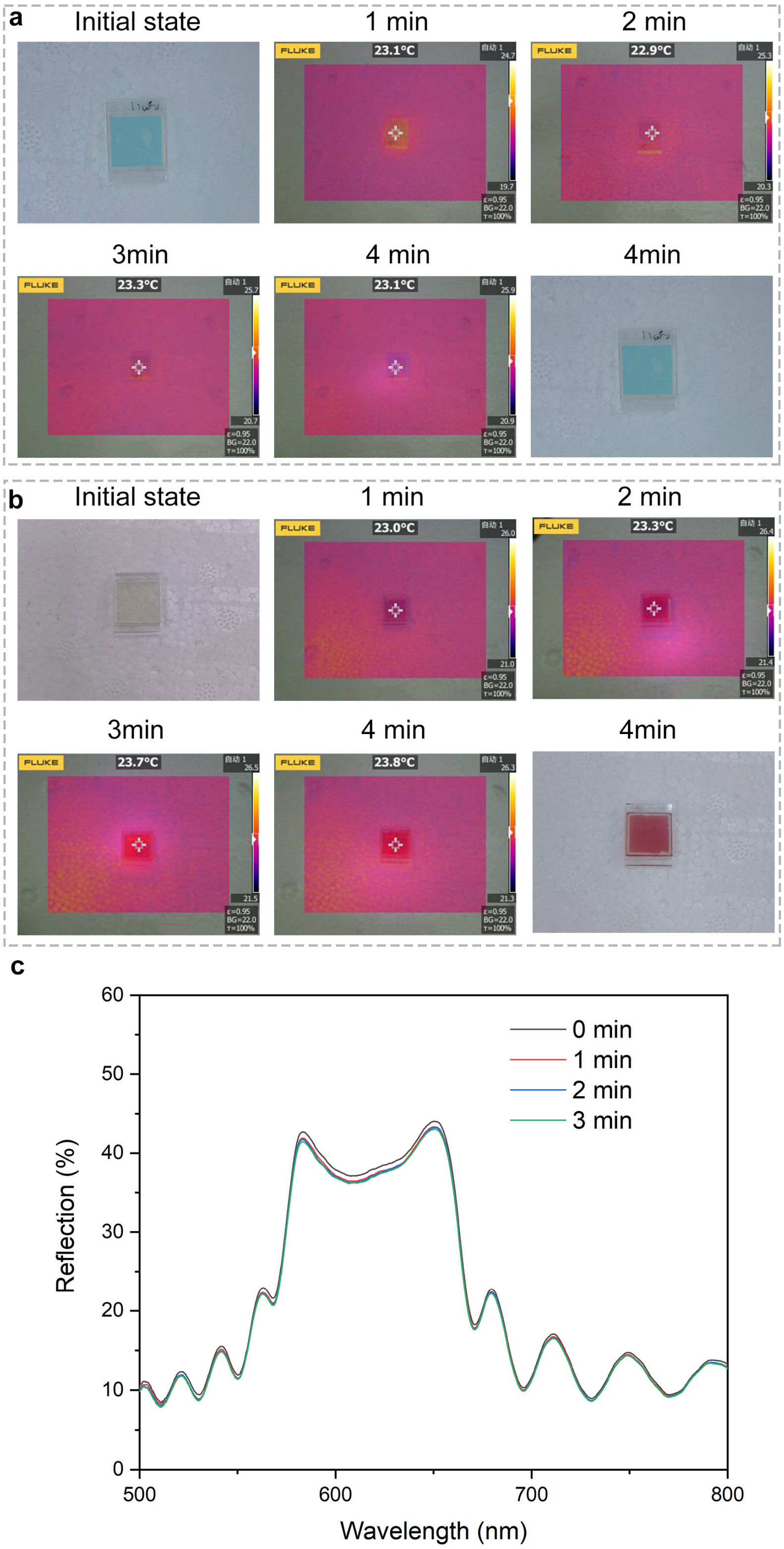

Figure S47. (a) Thermal effect of non photoresponsive helical LC (1.3 mol% R5011 in E7) irradiated with UV light (365 ± 20 nm) by infrared thermal imager. **(b)** Thermal effect of photoresponsive helical LC (*P*-**1o** (1.98 mol%) in E7) irradiated with UV light (365 ± 20 nm) by infrared thermal imager. **(c)** Changes of reflection spectra of non photoresponsive helical LC (1.3 mol% R5011 in E7) by UV light (365 ± 20 nm). The results clearly show that there is almost no thermal effect by UV irradiation and almost no effect on the reflection band

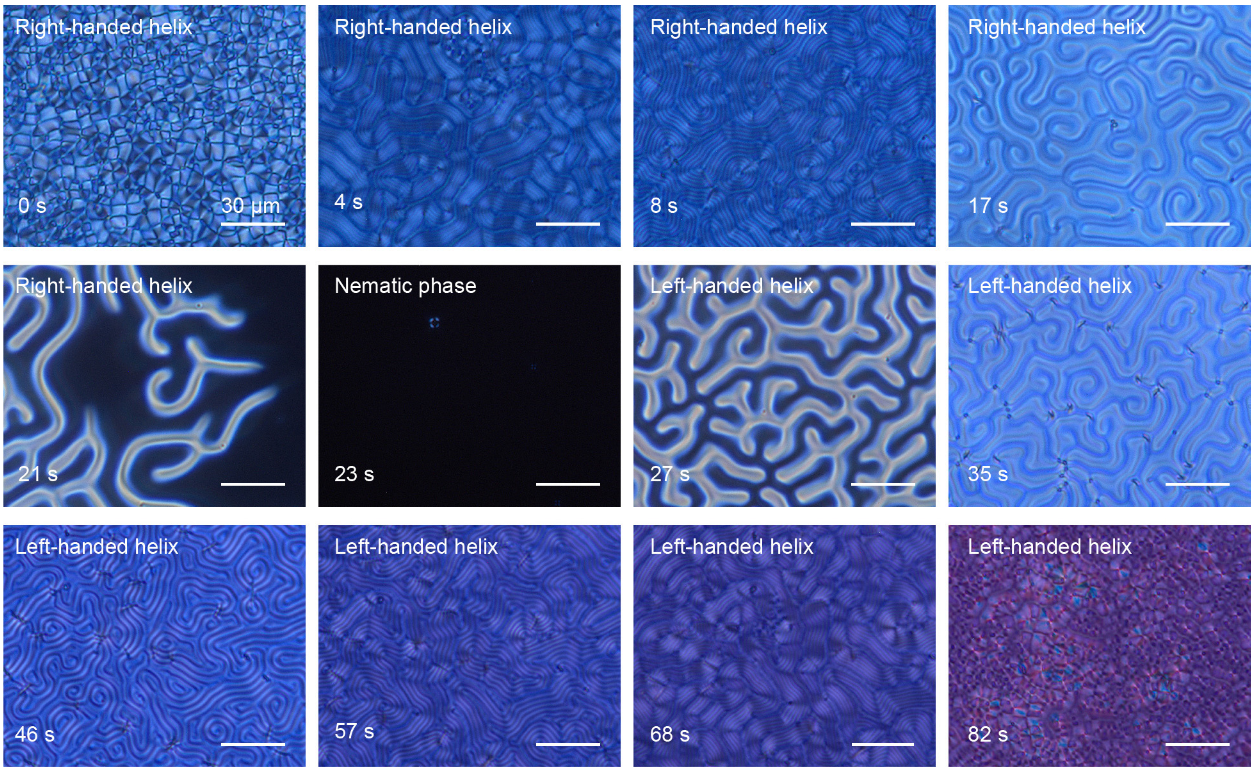

Figure S48 Polarized optical microscopy (POM) images of the chiral inversion of the photoresponsive LCs in E7 in a 4-μm thick homeotropic cell upon exposure to 365 nm at different time.

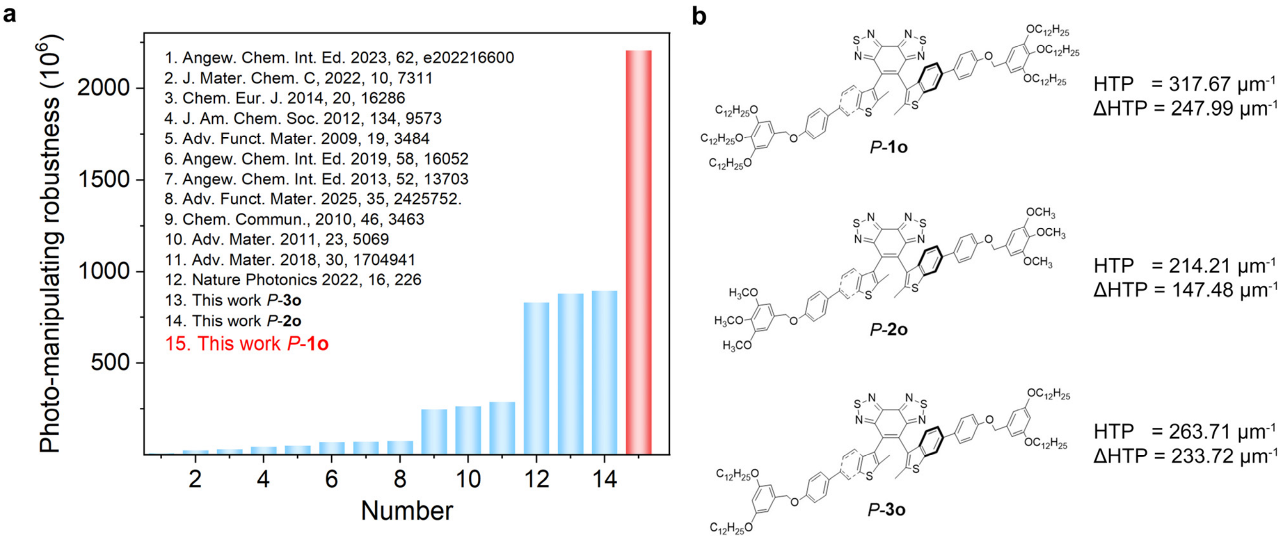

Figure S49 Photo-manipulating robustness of different chiral photoswitch. (a) Comparison of photo-manipulating robustness (R_P) of different chiral photoswitch. **(b)** Structure of *P*-**1o**, *P*-**2o**, *P*-**3o** and corresponding HTP value and variations.

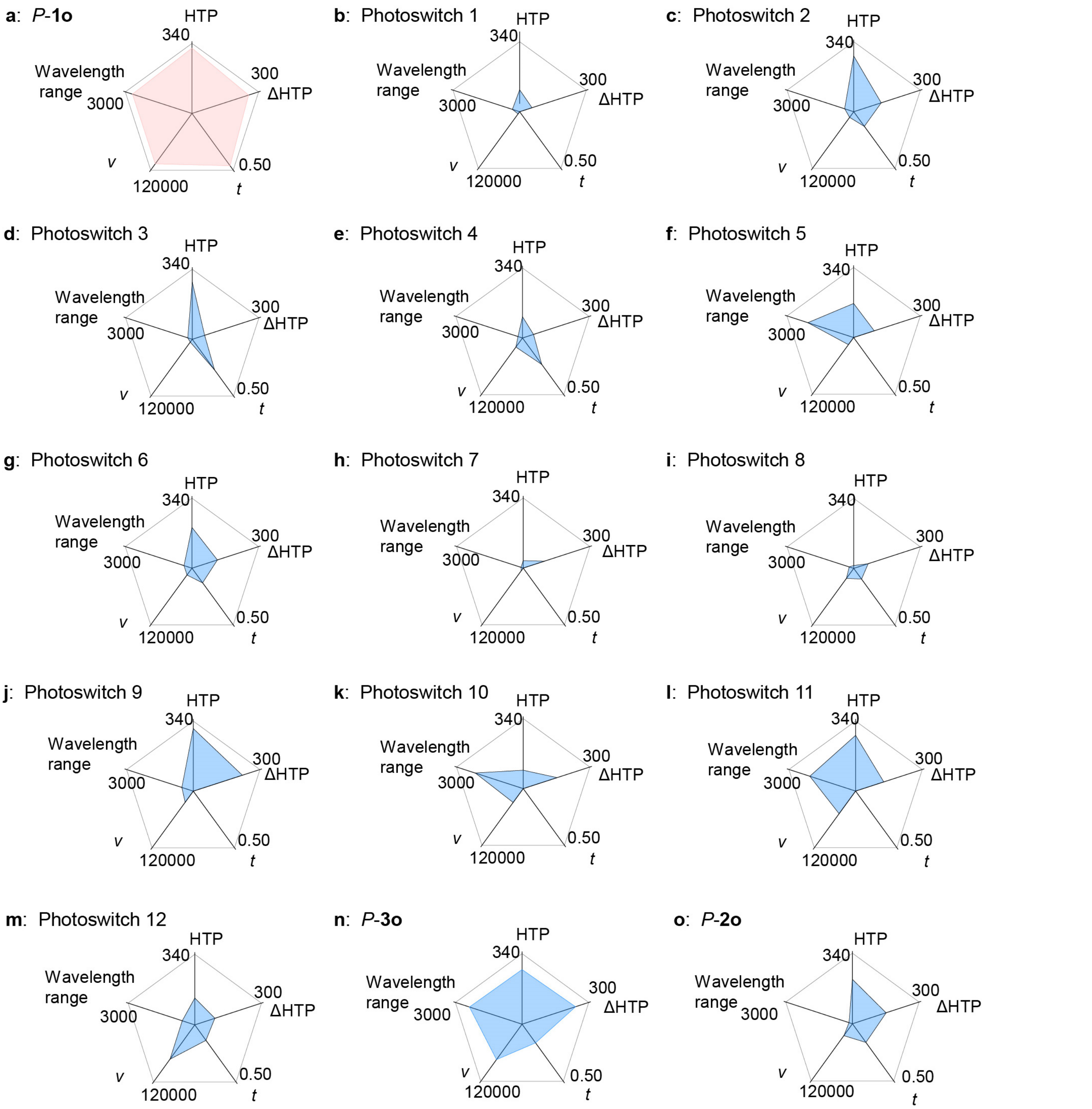

Figure S50 The comparison of HTP, ΔHTP, thermal stability time, response speed and reflection wavelength range of different chiral photoswitch: **(a)** *P*-**1o**, **(b)** photoswitch 1, **(c)** photoswitch 2, **(d)** photoswitch 3, **(e)** photoswitch 4, **(f)** photoswitch 5, **(g)** photoswitch 6, **(h)** photoswitch 7, **(i)** photoswitch 8, **(j)** photoswitch 9, **(k)** photoswitch 10, **(l)** photoswitch 11, **(m)** photoswitch 12, **(n)** *P*-**3o**, **(o)** *P*-**2o**. Notes: The "*t*" denotes the thermal stability time, with unit of "month". The "*v*" denotes the average shift rate of the reflection spectra of chiral photoswitch, with unit of "μm $month^{-1}$". Wavelength range means the manipulation range of reflection spectra, with unit of "nm". Detailed information sees in Table S2.

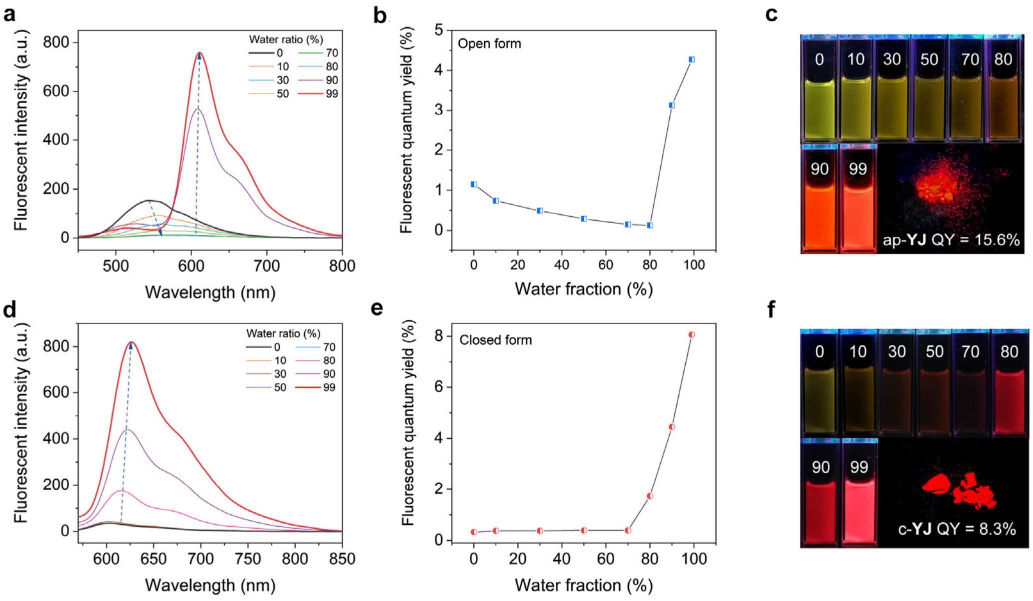

Figure S51. **(a-c)** Fluorescent spectra and relative fluorescent quantum yield of **1o** and **(d-f)**, **1c** in THF/H_2O mixtures with different water volume ratios. Photographs of fluorescence of **(c)** **1o** and **(f)** **1c** in different water ratio under UV light (365 ± 20 nm).

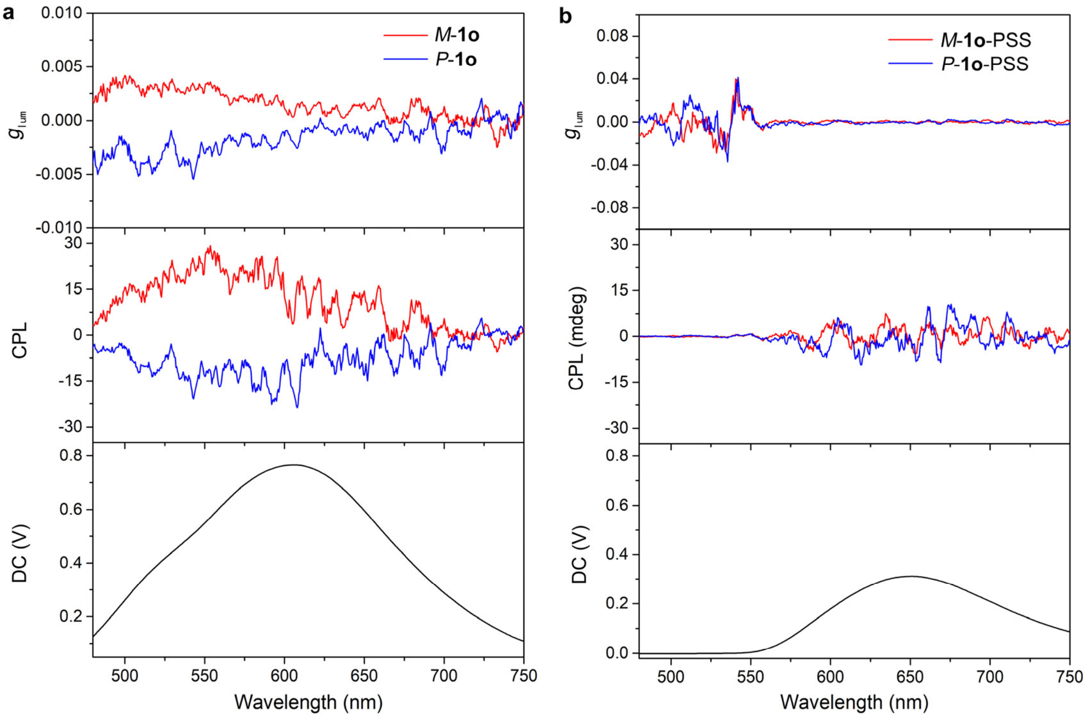

Figure S52. **(a)** The g_{lum}, CPL and DC spectra of *M*-**1o** and *P*-**1o**. **(b)** The g_{lum}, CPL and DC spectra of *M*-**1o** PSS and *P*-**1o** PSS upon excitation at 326 nm after UV light irradiation at 365 ± 20 nm in THF solutions. Note: The DC spectra are considered as the emission spectra of the luminescent materials collected by JASCO CPL spectrometer. The g_{lum} is calculated as: g_{lum}= CPL (mdeg) ÷ DC (V) × 0.000069813.

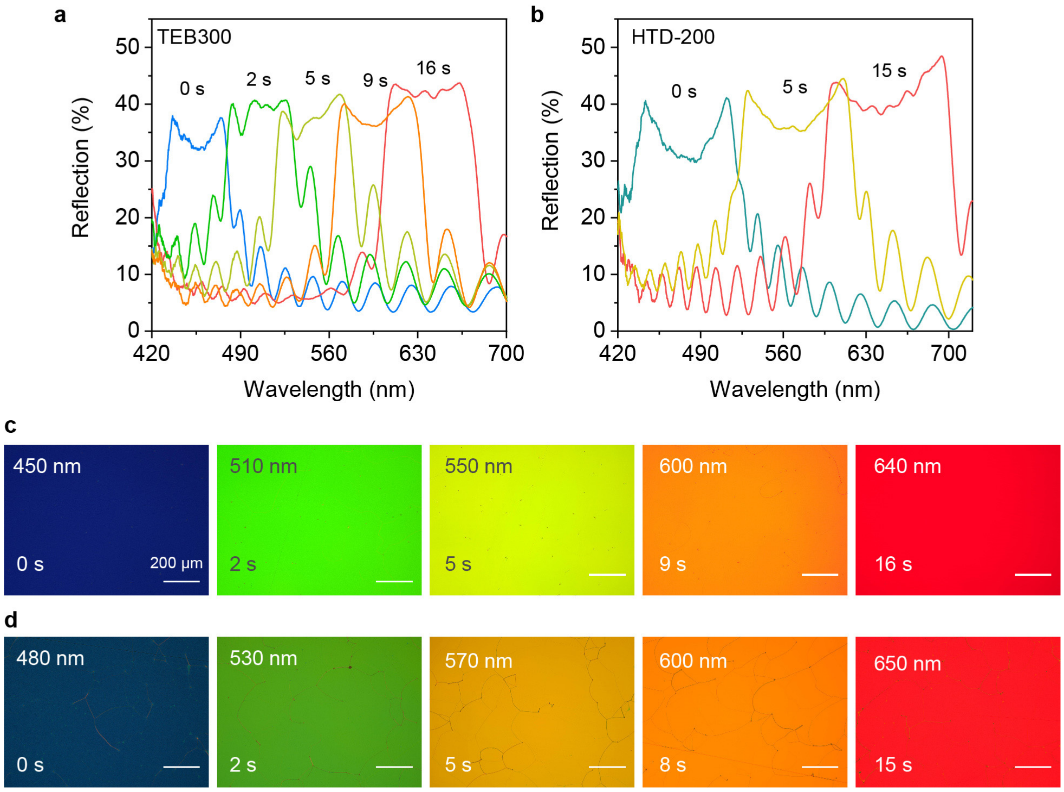

Figure S53. Reflection wavelength of photoresponsive helical LC containing **(a)** 0.31 mol% *P*-**1o** and 1.1 mol% R5011 in commercially available LC (TEB300) and **(b)** 0.39 mol% *P*-**1o** and 1.6 mol% R5011 in commercially available LC (HTD-200) in a 4-μm thick planar cell upon exposure to 365 ± 20 nm with different time. Reflection color change of photoresponsive helical LC containing **(c)** 0.31 mol% *P*-**1o** and 1.1 mol% R5011 in commercially available LC (TEB300) and **(d)** 0.39 mol% *P*-**1o** and 1.6 mol% R5011 in commercially available LC (HTD-200) in a 4-μm thick planar cell upon exposure to 365 ± 20 nm with different time.

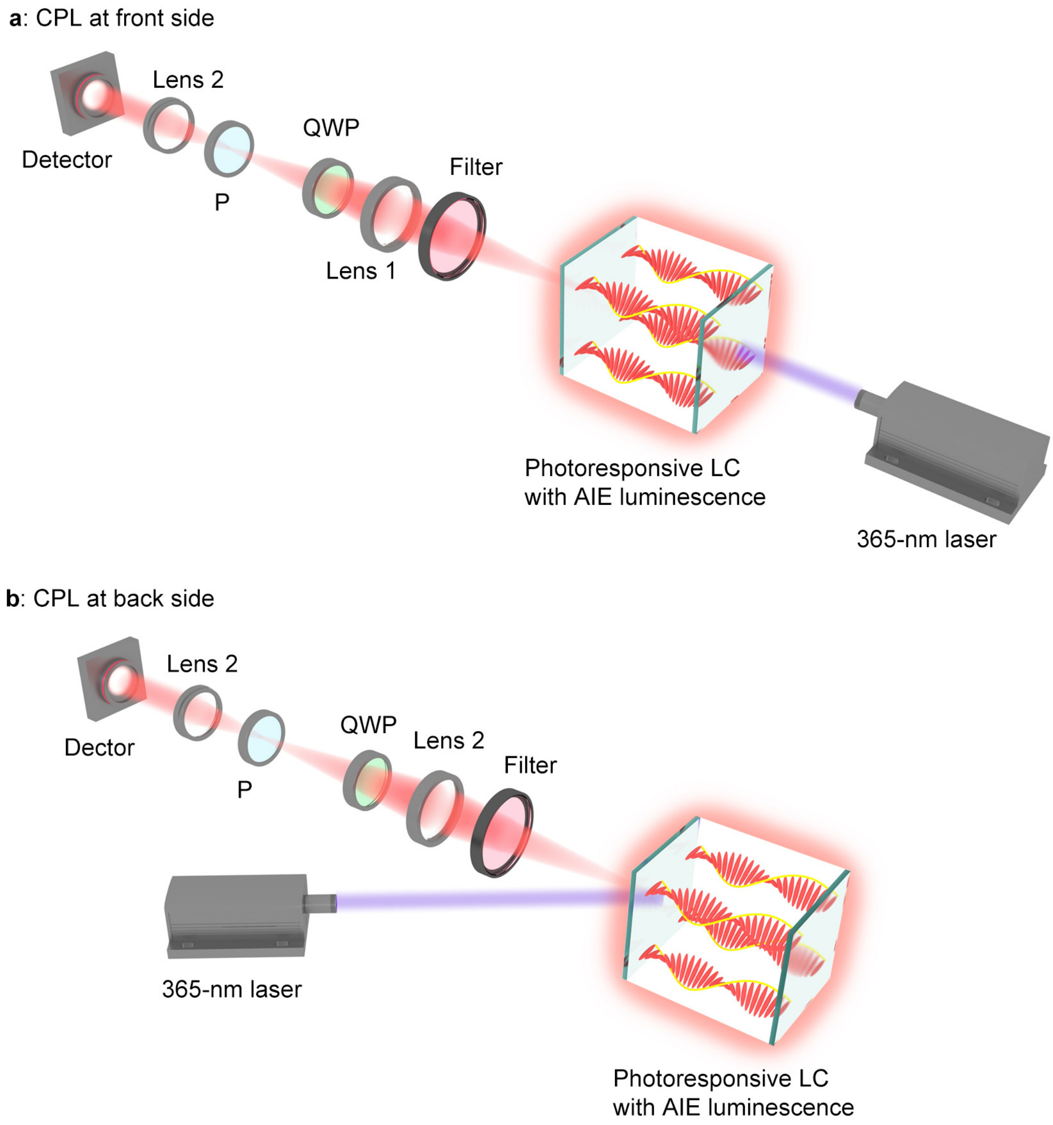

Figure S54 The detailed schematic illustration of the optical setup of the CPL polarization evaluation through azimuthal angle-dependent transmission **(a)** at the front side and **(b)** at the back side.

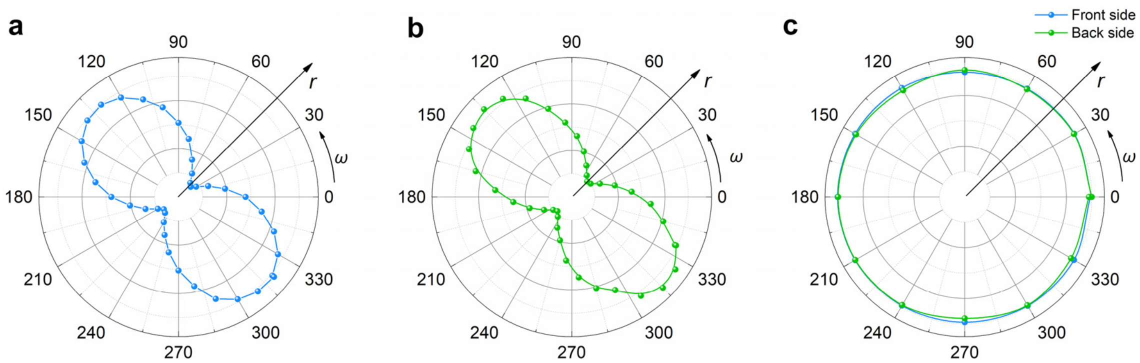

Figure S55. Emission intensity in HTD-200 of circularly polarized luminescence at 610 nm as a function of the polarization angle in the polar coordinate system: Light intensity of CPL of photoresponsive LC (0.39 mol% *M*-**1o** and 1.6 mol% S5011)

at **(a)** front side and **(b)** back side of LC cell after passing through a quarter waveplate and the polarizer; **(c)** Light intensity of CPL of photoresponsive LC (0.39 mol% *M*-**1o** and 1.6 mol% S5011) at front side and back side of the LC cell after passing through the polarizer. We convert the CPL into a linear polarization by passing it through a quarter waveplate, manifesting that both sides of the cell exhibited the same handed circular polarization, indicating the mechanism of the coupling between luminescence and photonic resonance in the LC helix structure.

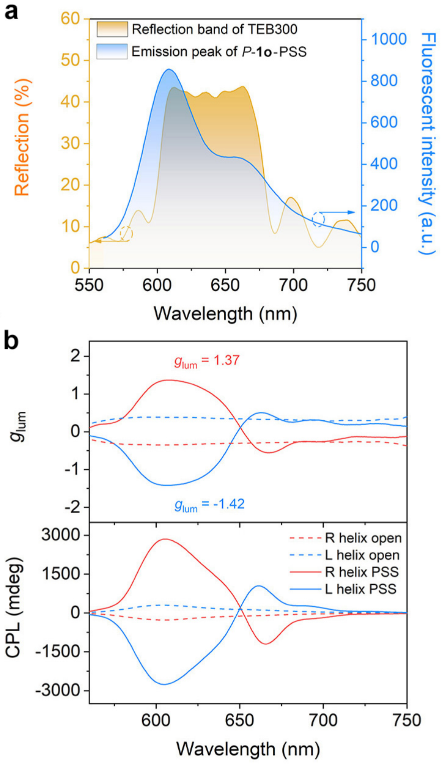

Figure S56. (a) Reflection spectra of the helical superstructure and emission spectrum showing incomplete overlap with the AIE peak of *P*-**1o** at PSS in the LCs. **(b)** The g_{lum} and CPL spectra upon excitation at 326 nm when the reflection spectra totally (solid lines) and marginally (dash lines) overlap with luminescence, respectively.

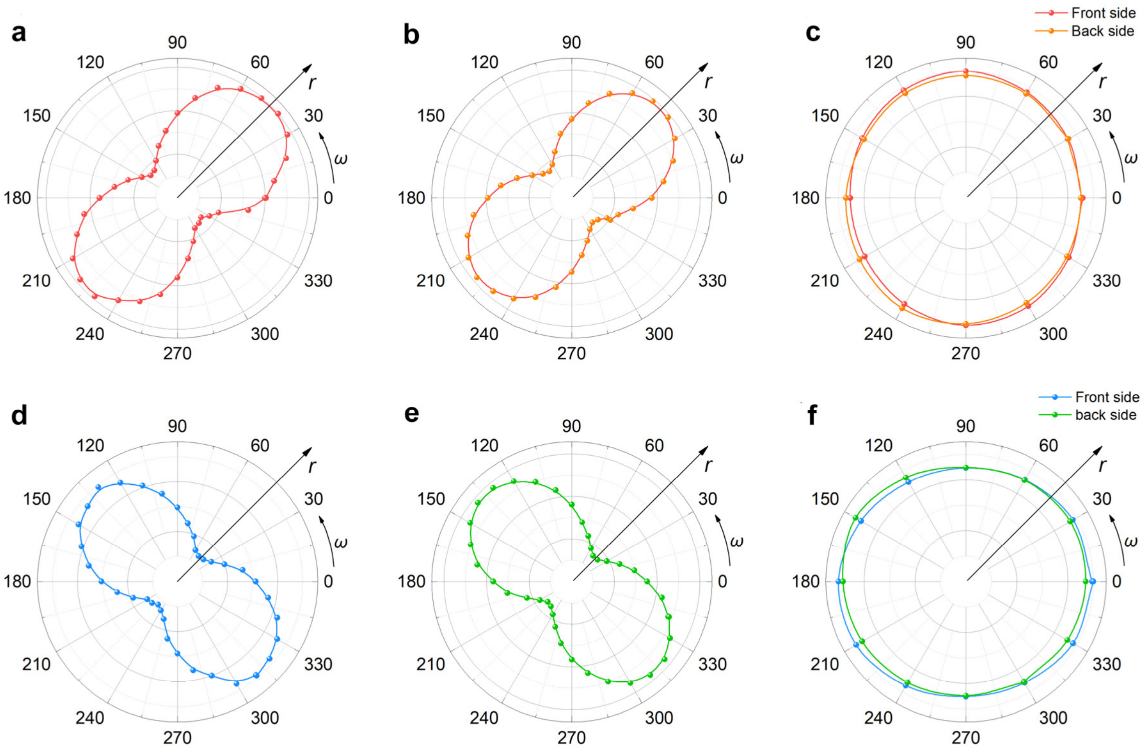

Figure S57. Emission intensity in TEB300 of circularly polarized luminescence at 610 nm as a function of the polarization angle in the polar coordinate system: Light intensity of CPL of photoresponsive LC (0.31 mol% *P*-**1o** and 1.1 mol% R5011) at **(a)** front side and **(b)** back side of LC cell after passing through a quarter waveplate and the polarizer; **(c)** Light intensity of CPL of photoresponsive LC (0.31 mol% *P*-**1o** and 1.1 mol% R5011) at front side and back side of LC cell after passing through the polarizer; Light intensity of CPL of photoresponsive LC (0.31 mol% *M*-**1o** and 1.1 mol% S5011) at **(d)** front side and **(e)** back side of LC cell after passing through a quarter waveplate and the polarizer; **(f)** Light intensity of CPL of photoresponsive LC (0.31 mol% *M*-**1o** and 1.1 mol% S5011) at front side and back side of LC cell after passing through the polarizer. The polar angle ω stands for the transmission angle of the polarizer and the radius r stands for the transmittance. We convert the CPL into a linear polarization by passing it through a quarter waveplate, manifesting that both sides of the cell exhibited the same handed circular polarization, indicating the mechanism of the coupling between luminescence and photonic resonance in the LC helix structure.

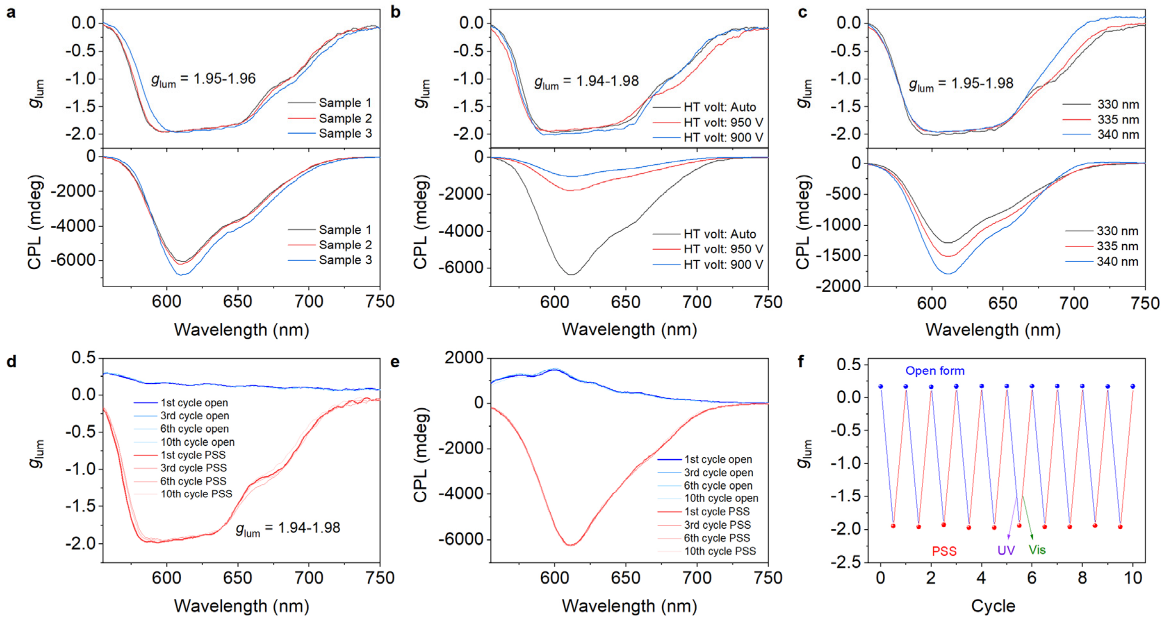

Figure S58 **(a)** The g_{lum} values and CPL spectra upon excitation at 326 nm of different batches samples when the reflection spectra totally overlap with luminescence; **(b)** The g_{lum} values and CPL spectra upon excitation at 326 nm by different excitation voltage when the reflection spectra totally overlap with luminescence; **(c)** The g_{lum} values and CPL spectra upon excitation at 326 nm by different excitation wavelength when the reflection spectra totally overlap with luminescence; **(d)** The g_{lum} spectra upon excitation at 326 nm and with auto excitation voltage in different cycles; **(e)** The CPL spectra upon excitation at 326 nm and with auto excitation voltage in different cycles; **(f)** Reversible change of the g_{lum} values upon alternate irradiations with UV (λ = 365 nm) and visible light (λ > 510 nm), respectively.

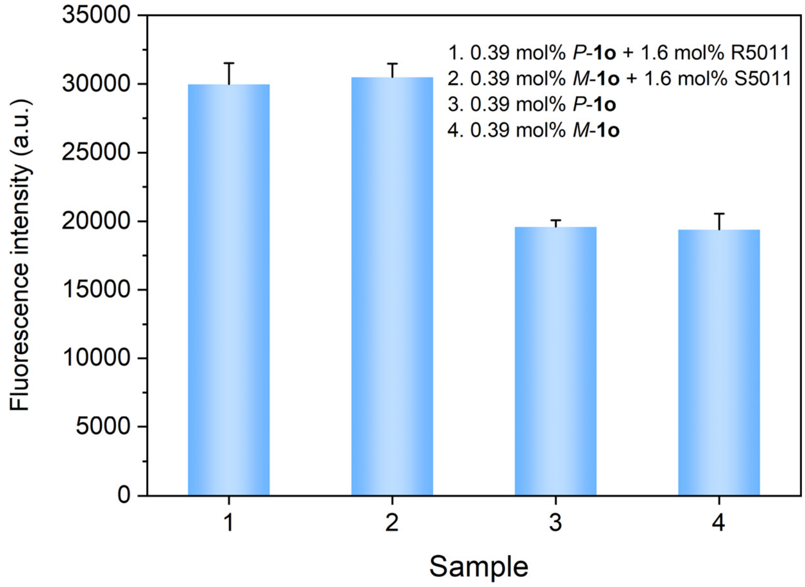

Figure S59. Different fluorescent intensity of the luminescent helical LC when the reflection spectra completely (sample 1 and 2) and marginally (sample 3 and 4) overlap with emission peak. When the reflection spectra totally overlap with the emission peak, the emission intensity of the CPL system showed stronger fluorescent intensity. In contrast, relatively weak

emission could be obtained when the reflection spectra marginally overlap with emission. Such phenomena are obviously distinguished from traditional CPL based on selective reflection with 50% loss in emission intensity, which may be ascribe to the band edge enhancement effect of photon resonance.

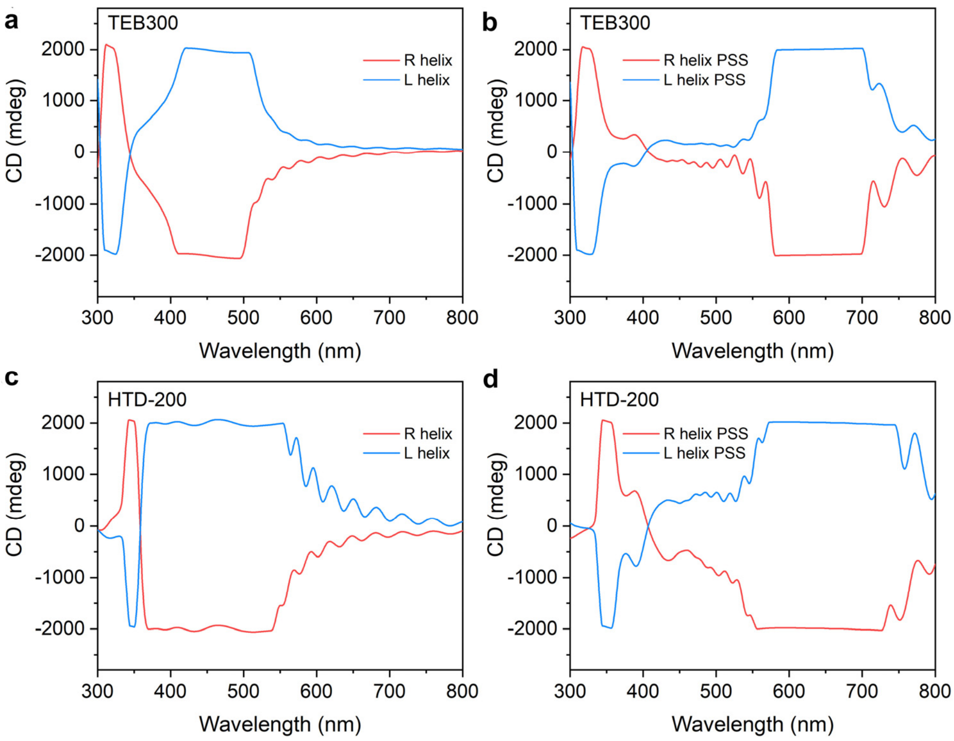

Figure S60. CD spectra of photoresponsive helical LC with 0.31 mol% *P*-**1o** and 1.1 mol% R5011 (red line) as well as 0.31 mol% *M*-**1o** and 1.1 mol% S5011 (blue line) in TEB300 **(a)** before and **(b)** after UV (365 ± 20 nm) irradiation, similar in HTD-200 (0.39 mol% *P*-**1o** and 1.6 mol% R5011, red line, as well as 0.39 mol% *M*-**1o** and 1.6 mol% S5011, blue line), **(c)** before and **(d)** after UV irradiation. Before irradiation, the reflection band marginally overlaps with the emission spectrum of the fluorescent dye. Thus, the chiral transfer effect from the chiral LC to the luminescent photoswitch predominates the CPL process. After irradiation, the broad reflection band totally overlaps with the emission spectrum. The CD spectra show strong signal at the reflection spectra, for example, an extremely strong negative cotton effect for right-handed helix. Thus, the LC only can emit the CPL with opposite handedness, illustrating chiral dependence of the helix structure on the CPL.

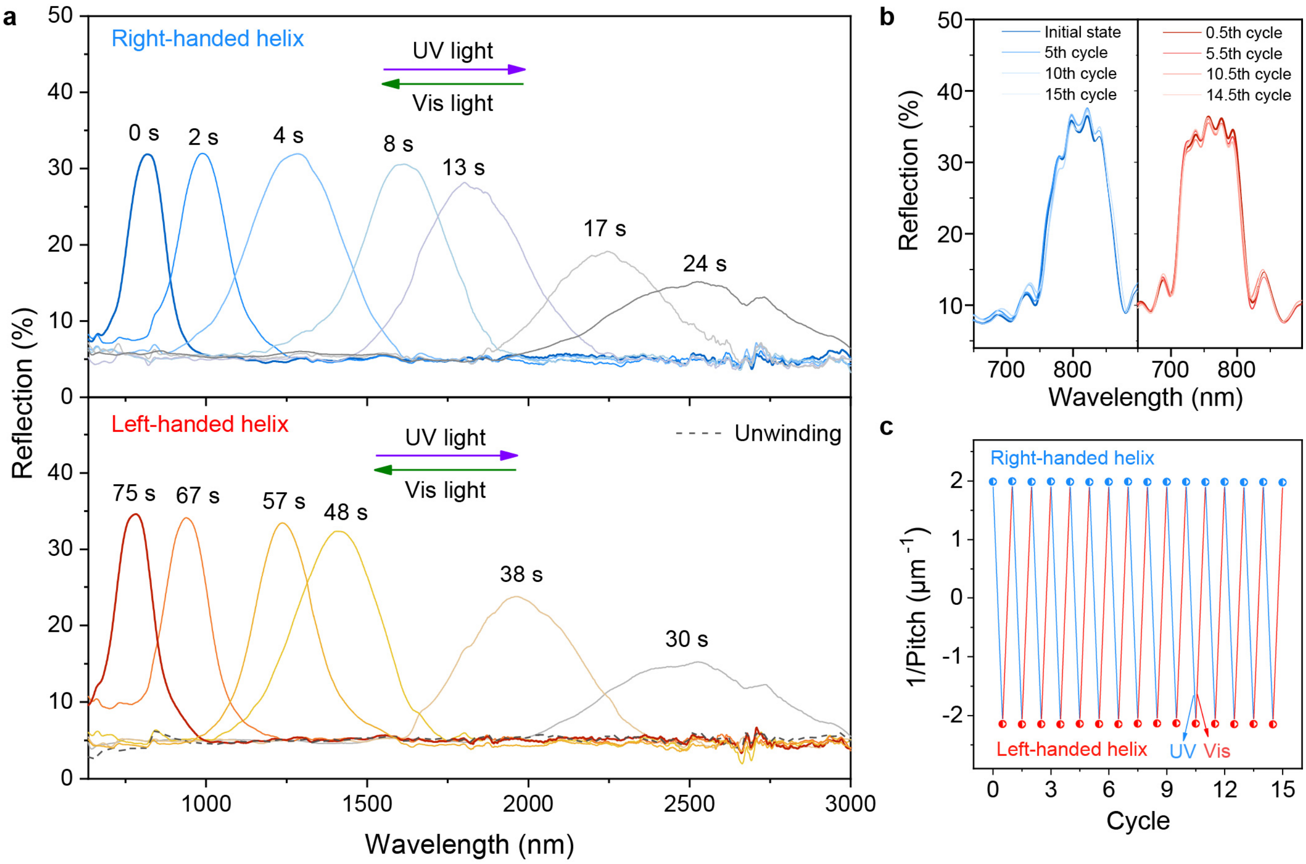

Figure S61. (a) Reflection wavelength of LC film containing 2.16 mol% *P*-**1o** and 1.54 mol% S5011 in commercially available LCs in a 4-μm thick planar cell upon exposure to 365 nm light with different irradiation time. **(b)** Reflection spectra of the photoresponsive helical LC in different cycles. **(c)** Reversible change of the helix pitch upon alternate irradiations with UV (λ = 365 ± 20 nm) and visible light (λ > 510 nm), respectively. The reciprocal of the pitch is to better express the reversible change of the helix inversion.

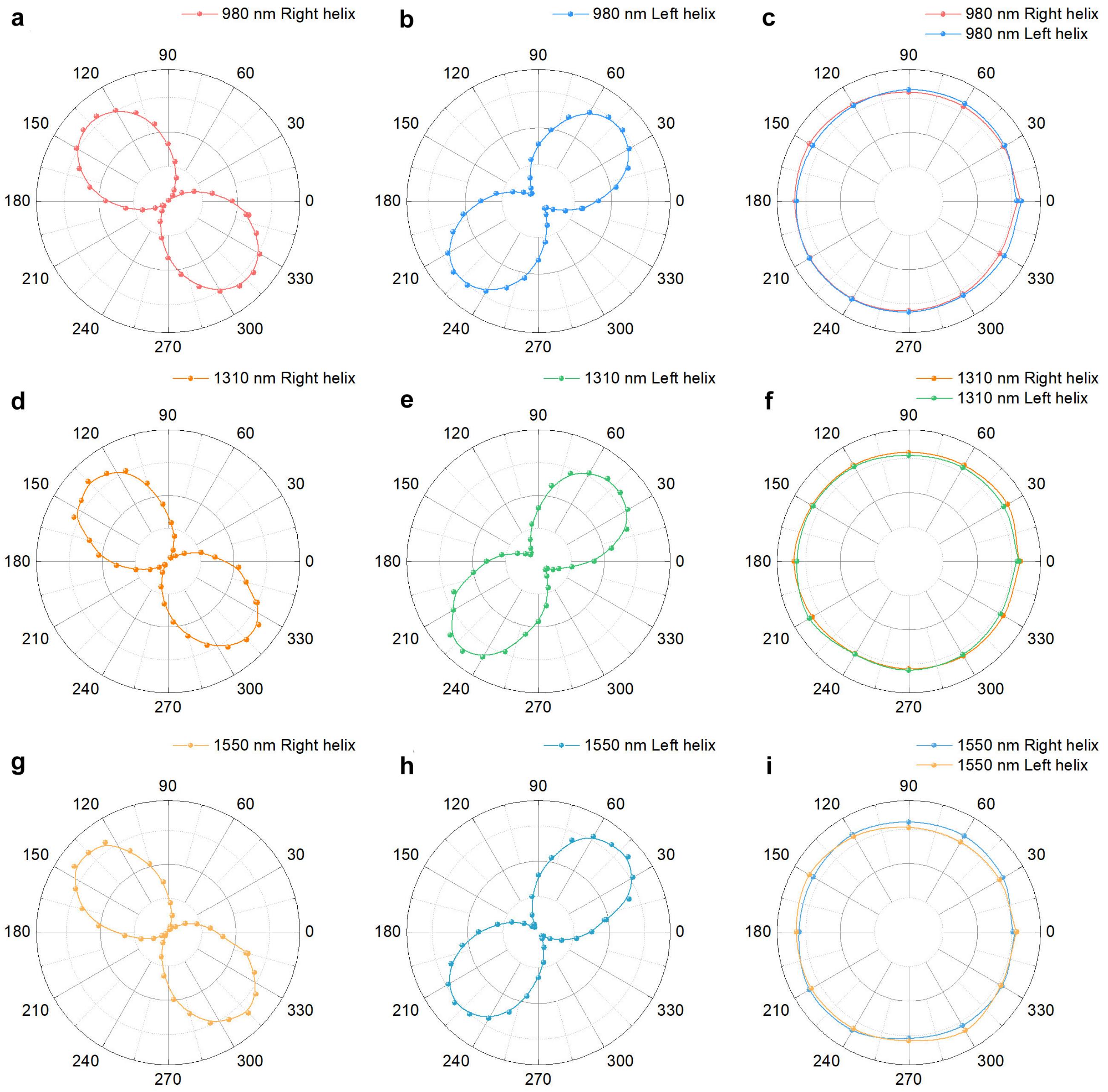

Figure S62. Characterization of photo-invertible helical LC by detecting polarization state of reflected light. Light intensity of 980 nm, 1310 nm and 1550 nm after reflected by photo-invertible helical LC as a function of the polarization angle in the polar coordinate system. Reflected light by right-handed helical LC at **(a)** 980 nm, **(d)** 1310 nm and **(g)** 1550 nm after passing through a quarter waveplate and the polarizer. Reflected light by left-handed helical LC at **(b)** 980 nm, **(e)** 1310 nm and **(h)** 1550 nm after passing through a quarter waveplate and the polarizer. Reflected light by right- and left-handed helical LC at **(c)** 980 nm, **(f)** 1310 nm and **(i)** 1550 nm after passing through the polarizer

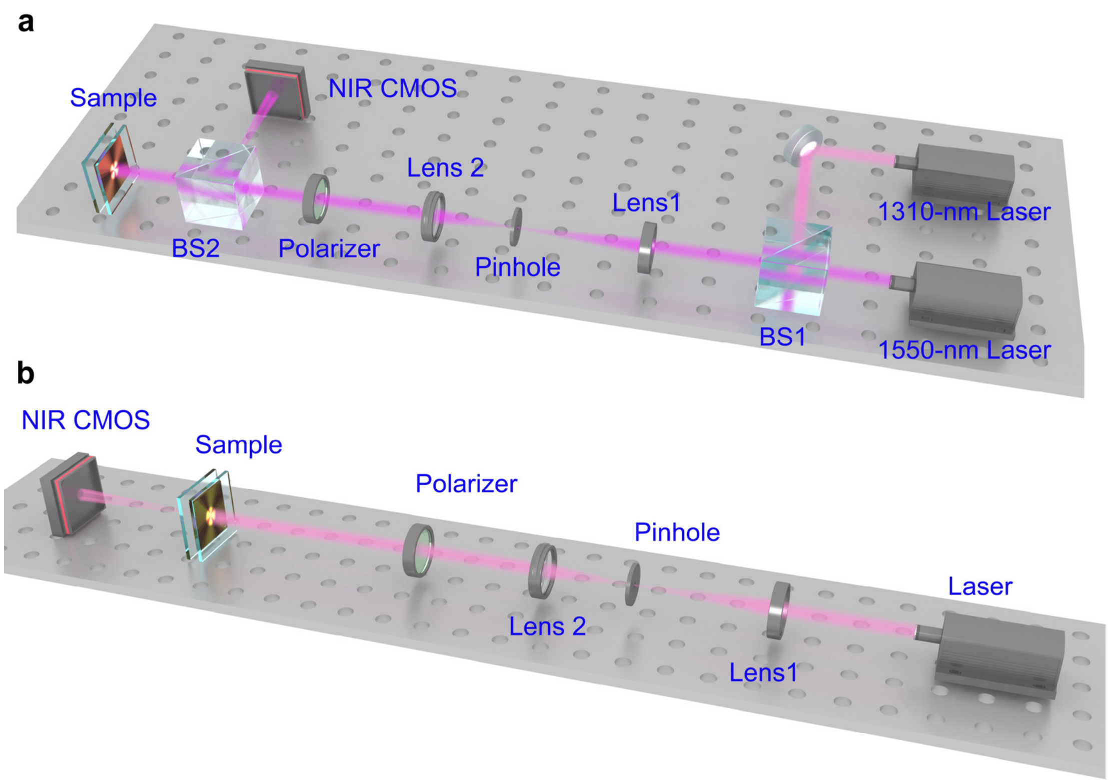

Figure S63. Optical setup of reflected **(a)** vortex and transmitted **(b)** vector beam. BS: beamsplitter.

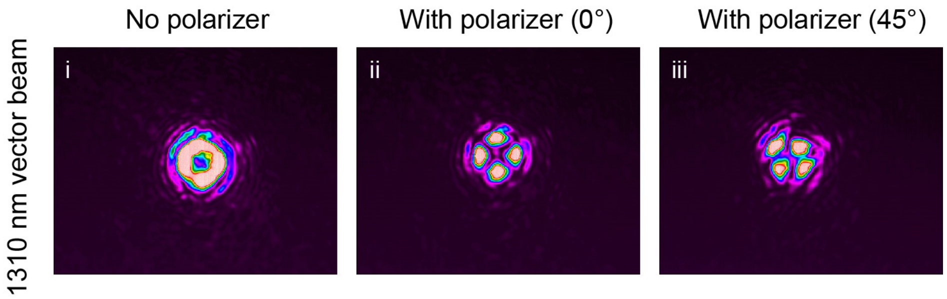

Figure S64. Vector beam at 1310 nm and corresponding transmission patterns a polarizer with different angle (0° or 45°).

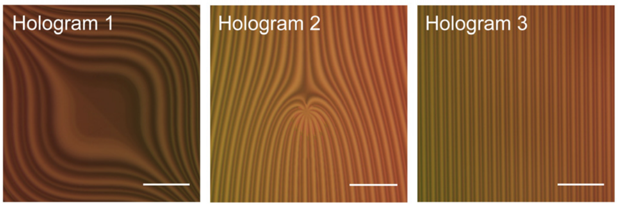

Figure S65. Three holograms to generate diffraction patterns. Hologram 1:Airy beam phase plate, Hologram 2: fork grating, and Hologram 3: polarization grating. The scale bar is 200 μm.

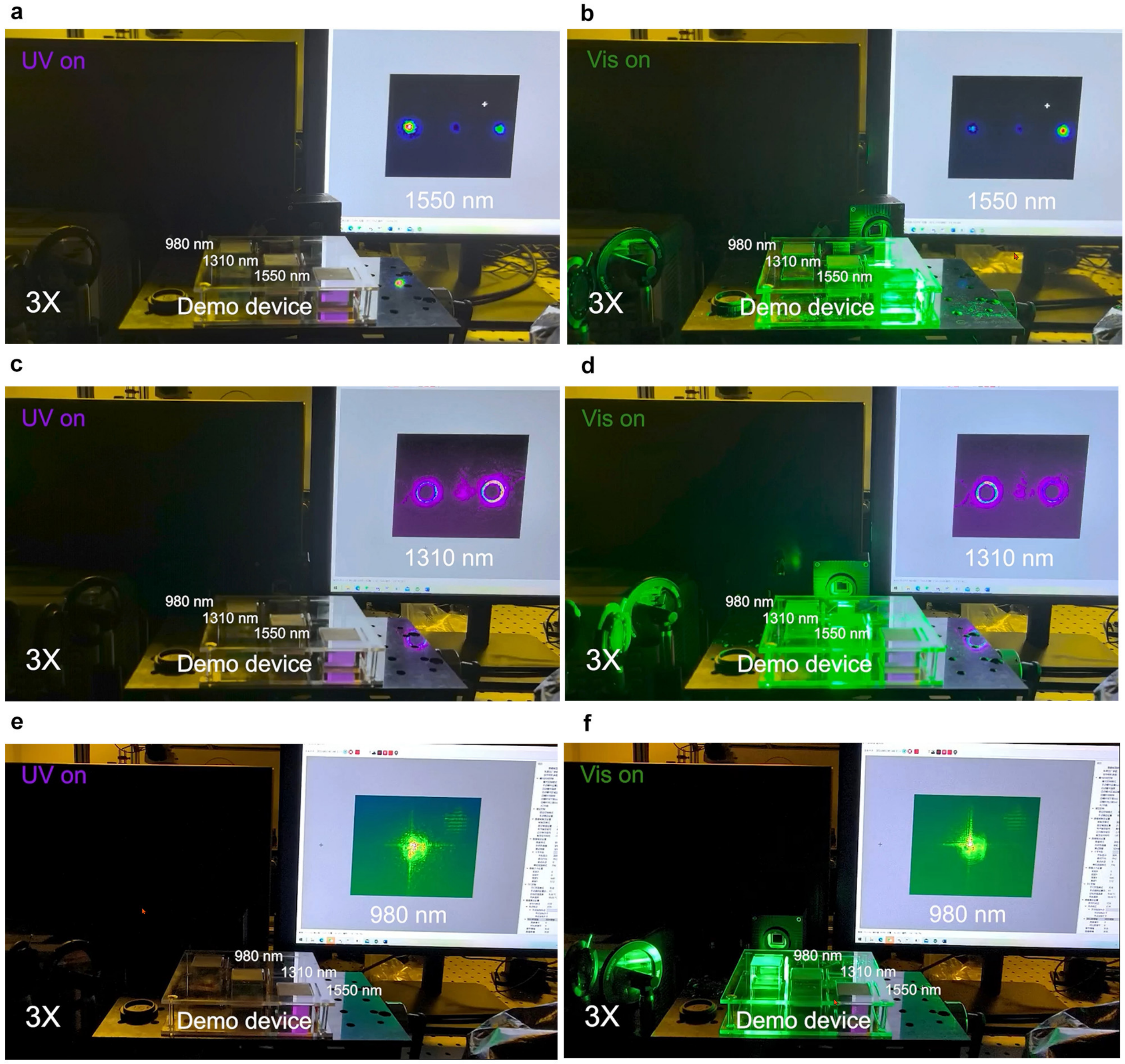

Figure S66 The actual effect of integrate device for ternary spatial information coding by UV and visible light irradation. (a), (b) Diffraction pattern of polarization grating detected 1550-nm light showed switch between right- and left-handed CP light upon irradiated by UV (365 nm) and visible light (532 nm) in demo device. **(c), (d)** Diffraction pattern of fork grating detected 1310-nm light showed switch between right- and left-handed CP light upon irradiated by UV (365 nm) and visible light (532 nm) in demo device. **(e), (f)** Diffraction pattern of Airy beam detected 980-nm light showed switch between right- and left-handed CP light upon irradiated by UV (365 nm) and visible light (532 nm) in demo device.

4. Supplementary table

Table S1 Helical twisting power (HTP) of *P*-**1o**, *P*-**2o** and *P*-**3o** at different states in LC host.

Photoswitches	HTP at open form (μm^{-1})	HTP at PSS (μm^{-1})	ΔHTP (μm^{-1})
P-**1o**	317.67	69.68	247.99
P-**2o**	214.21	66.73	147.48
P-**3o**	263.71	29.99	233.72

Table S2 Photo-manipulating robustness comparison of different chiral photoswitches

No.	Photoswitches	*t* (month)	ΔHTP (μm^{-1})	*c* (mol/mol)	*v*[b] (μm month^{-1})	$R_P \times 10^6$	Ref.
**15**	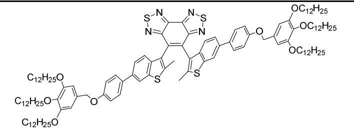	**0.450**	**247.99**	**0.0198**	**112320**	**2206**	**This work *P*-1o**
1	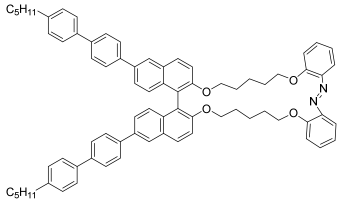	0[a]	66.7	0.031	4406.4	9.48	[5]
2	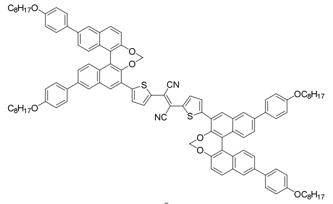	0.133	121	0.062	11664	26.0	[6]
3	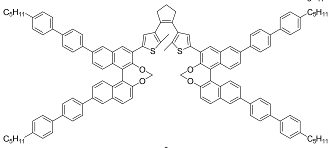	0.267	55	0.013	5702.4	31.49	[7]
4	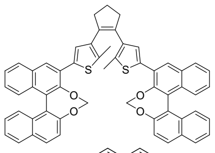	0.233	49	0.026	18662.4	44.41	[8]
5	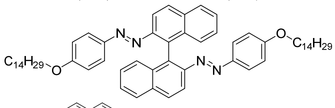	0[a]	91	0.026	14774.4	51.71	[9]
6	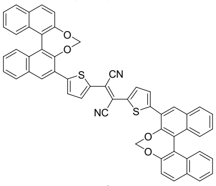	0.133	112	0.025	13996.8	71.64	[10]
7	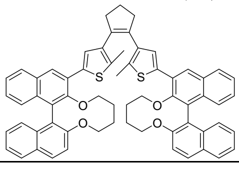	0[a]	94	0.0077	5961.6	72.78	[11, 12]

8	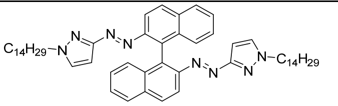	0	64	0.017	20736	78.06	[13]
9	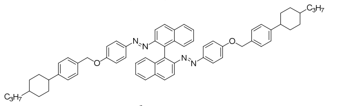	0[a]	215	0.02	23068.8	247.9	[14]
10	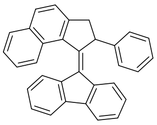	0[a]	149	0.016	28512	265.5	[15]
11	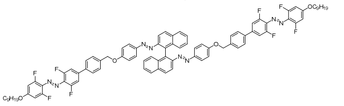	0[a]	124	0.02	46656	289.2	[16]
12	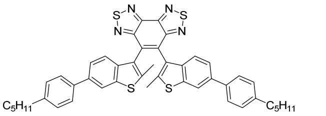	0.139	88	0.0085	69984	832.4	[12]
13	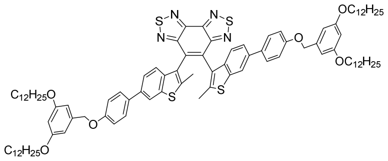	0.166	233.72	0.0227	72450	880	This work *P*-**3o**
14	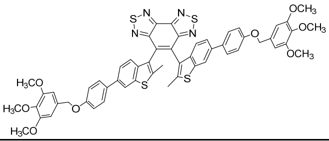	0.166	147.48	0.0047	24192	896	This work *P*-**2o**

[a]: Non-stable photoswitch don't show any stability time.
[b]: Here, *v* represents the average shift speed of the reflection band until reach photostationary state. And the huge value has been carried out due to unit conversion.

Table S3 Fluorescent quantum yield of **1o** in different state and different LC hosts

	Fluorescent quantum yield (%) in TEB300		Fluorescent quantum yield (%) in HTD-200	
	R helix	S helix	R helix	S helix
Open form	19.19	18.20	18.31	20.96
PSS	21.92	24.79	21.99	21.68

5. References and Notes:

1. Yang M, Mao D, Chen S *et al.* Design, synthesis and thermotropic self-organization of dendronized polystyrenes with different length alkyl tails. *Polym Chem* 2016; **7**: 5445-55.

2. Li M, Zhu W-H. Sterically hindered diarylethenes with a benzobis(thiadiazole) bridge: Enantiospecific transformation and reversible photosuperstructures. *Acc Chem Res* 2022; **55**: 3136-49.

3. Shao C, Grüne M, Stolte M *et al.* Perylene bisimide dimer aggregates: Fundamental insights into self-assembly by nmr and uv/vis spectroscopy. *Chem Eur J* 2012; **18**: 13665-77.

4. Yao Y, Xu D, Zhu Y *et al.* Dandelion flower-like micelles. *Chem Sci* 2020; **11**: 757-62.

5. Wang H, Tang Y, Bisoyi HK *et al.* Reversible handedness inversion and circularly polarized light reflection tuning in self‐organized helical superstructures using visible‐light‐driven macrocyclic chiral switches. *Angew Chem Int Ed* 2023; **62**: e202216600.

6. Qiao J, He Y, Lin S *et al.* A photoswitchable circularly polarized luminescent cholesteric superstructure: Direct visualization and dynamic modulation of the amplified luminescence dissymmetry factor. *J Mater Chem C* 2022; **10**: 7311-8.

7. Li Y, Wang M, Wang H *et al.* Rationally designed axially chiral diarylethene switches with high helical twisting power. *Chem Eur J* 2014; **20**: 16286-92.

8. Li Y, Urbas A, Li Q. Reversible light-directed red, green, and blue reflection with thermal stability enabled by a self-organized helical superstructure. *J Am Chem Soc* 2012; **134**: 9573-6.

9. White TJ, Bricker RL, Natarajan LV *et al.* Phototunable azobenzene cholesteric liquid crystals with 2000 nm range. *Adv Funct Mater* 2009; **19**: 3484-8.

10. Li J, Bisoyi HK, Lin S *et al.* 1,2-dithienyldicyanoethene-based, visible-light-driven, chiral fluorescent molecular switch: Rewritable multimodal photonic devices. *Angew Chem Int Ed* 2019; **58**: 16052-6.

11. Li Y, Xue C, Wang M *et al.* Photodynamic chiral molecular switches with thermal stability: From reflection wavelength tuning to handedness inversion of self-organized helical superstructures. *Angew Chem Int Ed* 2013; **52**: 13703-7.

12. Zheng Z, Hu H, Zhang Z *et al.* Digital photoprogramming of liquid-crystal superstructures featuring intrinsic chiral photoswitches. *Nat Photon* 2022; **16**: 226-34.

13. Zhang X, Tang Y, Ma Y *et al.* Azopyrazole-based axially chiral dopants with high thermal stability in cholesteric liquid crystals. *Adv Funct Mater* 2025; **35**: 2425752.

14. Ma J, Li Y, White T *et al.* Light-driven nanoscale chiral molecular switch: Reversible dynamic full range color phototuning. *Chem Commun* 2010; **46**: 3463-5.

15. White TJ, Cazzell SA, Freer AS *et al.* Widely tunable, photoinvertible cholesteric liquid crystals. *Adv Mater* 2011; **23**: 1389-92.

16. Qin L, Gu W, Wei J *et al.* Piecewise phototuning of self-organized helical superstructures. *Adv Mater* 2018; **30**: 1704941.
